# In Vitro Evaluation of the Potential Pharmacological Activity and Molecular Targets of New Benzimidazole-Based Schiff Base Metal Complexes

**DOI:** 10.3390/antibiotics10060728

**Published:** 2021-06-16

**Authors:** Alberto Aragón-Muriel, Yamil Liscano, Yulieth Upegui, Sara M. Robledo, María Teresa Ramírez-Apan, David Morales-Morales, Jose Oñate-Garzón, Dorian Polo-Cerón

**Affiliations:** 1 Laboratorio de Investigación en Catálisis y Procesos (LICAP), Departamento de Química, Facultad de Ciencias Naturales y Exactas, Universidad del Valle, Cali 760001, Colombia; alberto.aragon@correounivalle.edu.co; 2 Grupo de Investigación en Química y Biotecnología (QUIBIO), Facultad de Ciencias Básicas, Universidad Santiago de Cali, Cali 760031, Colombia; yamil.liscano00@usc.edu.co (Y.L.); jose.onate00@usc.edu.co (J.O.-G.); 3 PECET, Facultad de Medicina, Universidad de Antioquia, Medellín 050010, Colombia; yulieth.upegui@udea.edu.co (Y.U.); sara.robledo@udea.edu.co (S.M.R.); 4 Instituto de Química, Universidad Nacional Autónoma de México, Cd. Universitaria, Circuito Exterior, Coyoacán, México 04510, Mexico; mtrapan@unam.mx (M.T.R.-A.); damor@unam.mx (D.M.-M.)

**Keywords:** metal-based drugs, antiproliferative activity, antibacterial activity, antiparasitic activity, model membranes, molecular docking, DNA interaction, lanthanide complexes, Schiff bases

## Abstract

Metal-based drugs, including lanthanide complexes, have been extremely effective in clinical treatments against various diseases and have raised major interest in recent decades. Hence, in this work, a series of lanthanum (III) and cerium (III) complexes, including Schiff base ligands derived from (1*H*-benzimidazol-2-yl)aniline, salicylaldehyde, and 2,4-dihydroxybenzaldehyde were synthesized and characterized using different spectroscopic methods. Besides their cytotoxic activities, they were examined in human U-937 cells, primate kidney non-cancerous COS-7, and six other, different human tumor cell lines: U251, PC-3, K562, HCT-15, MCF-7, and SK-LU-1. In addition, the synthesized compounds were screened for in vitro antiparasitic activity against *Leishmania braziliensis*, *Plasmodium falciparum,* and *Trypanosoma cruzi*. Additionally, antibacterial activities were examined against two Gram-positive strains (*S. aureus* ATCC^®^ 25923, *L. monocytogenes* ATCC^®^ 19115) and two Gram-negative strains (*E. coli* ATCC^®^ 25922, *P. aeruginosa* ATCC^®^ 27583) using the microdilution method. The lanthanide complexes generally exhibited increased biological activity compared with the free Schiff base ligands. Interactions between the tested compounds and model membranes were examined using differential scanning calorimetry (DSC), and interactions with calf thymus DNA (CT-DNA) were investigated by ultraviolet (UV) absorption. Molecular docking studies were performed using leishmanin (1LML), cruzain (4PI3), *P. falciparum* alpha-tubulin (GenBank sequence CAA34101 [453 aa]), and *S.* *aureus* penicillin-binding protein 2a (PBP2A; 5M18) as the protein receptors. The results lead to the conclusion that the synthesized compounds exhibited a notable effect on model membranes imitating mammalian and bacterial membranes and rolled along DNA strands through groove interactions. Interactions between the compounds and studied receptors depended primarily on ligand structures in the molecular docking study.

## 1. Introduction

In medicinal chemistry, a large number of studied coordination compounds have demonstrated biological properties. One advantage of the unique electronic structures presented by transition metals is the versatility and ability to adjust the properties of a given molecule, which is not always possible for organic drugs [1]. The application of a compound as a therapeutic or diagnostic agent depends on coordination geometries, reactivity, and physicochemical properties [2,3,4]. When choosing organic ligands for coordination with metallic centers, the compounds should present different coordination points that may exhibit biological activity [5]. Schiff bases are known as privileged ligands because they are easy to obtain and can form multidentate complexes with various metallic centers due to the presence of donor heteroatoms, which generally appear in their structures as nitrogen and oxygen molecules [6,7].

Schiff bases derived from benzimidazoles are quite attractive for medicinal chemistry applications due to the broad biological activity of the benzimidazole ring [8], which has been characterized with antitumor (bendamustine), antimicrobial (albendazole), anti-ulcerative (omeprazole), anti-inflammatory (benoxaprofen analog), antihypertensive (candesartan), and antiviral (enviradine) activities [9,10,11]. The benzimidazole moiety is considered to be a structural isostere of purine and, therefore, inhibits the biosynthesis of nucleic acids and proteins [12]. In addition, benzimidazole can cause the selective degeneration of tubulin microtubules [13] and induce apoptosis and DNA fragmentation [14,15]. Molecular coupling studies have demonstrated interactions between benzimidazole ring derivatives and the topoisomerase IA complex in both Gram-positive [16] and Gram-negative [17] bacteria through hydrogen bonding and electrostatic interactions. In addition, in silico tests performed on the FPK protein showed that benzimidazole derivatives are likely to inhibit the glycolysis cycle in *Trypanosoma cruzi*, resulting in antiparasitic activity [18].

Schiff base ligands and their metal complexes based on benzimidazole intercalate with DNA, binding to the minor groove of DNA [19,20,21,22]. Some Schiff bases coordinated with lanthanide ions have been examined in-depth due to their low toxicity profiles and high levels of biological activities after ligand binding [23]. Recently, the research community has shown great interest in the synthesis of lanthanide-based complexes due to their broad biological activities as antimicrobials, chemotherapeutics, and contrast agents [24,25].

Metal complexes containing Schiff base ligands can display synergistic effects due to the presence of the benzimidazole derivatives. Hence, following our continuous interest in the design, production, and biological studies of lanthanide species [26,27,28], this paper includes results on the synthesis and characterization of La(III) and Ce(III) compounds, including Schiff base ligands obtained from (1*H*-benzimidazol-2-yl)aniline, salicylaldehyde, and 2,4-dihydroxybenzaldehyde. Cytotoxic activities were evaluated in U-937 human cells (ATCC^®^ CRL1593.2), non-cancerous monkey kidney cell line COS-7, and six different human tumor cell lines (U251, human glioblastoma; PC-3, prostate cancer (ATCC® CRL-1435); K562, myelogenous leukemia (ATCC^®^ CCL-243); HCT-15, colorectal carcinoma (ATCC^®^ CCL-225); MCF-7, breast epithelial adenocarcinoma (ATCC^®^ CRL-3435); SKLU-1, lung carcinoma). Besides, in vitro antiparasitic activity was studied against three parasitic protozoa: *Leishmania braziliensis* (MHOM/88/COL/UA301), *Plasmodium falciparum,* and *Trypanosoma cruzi* (Tulahuen strain). Additionally, the antibacterial activity of these complexes was explored with Gram-positive strains (*Staphylococcus aureus* ATCC^®^ 25923 and *Lysteria monocytogenes* ATCC® 19115) and Gram-negative strains (*Escherichia coli* ATCC^®^ 25922 and *Pseudomonas aeruginosa* ATCC^®^ 27583) with the microdilution method. Furthermore, interactions between the compounds and membrane models were evaluated employing differential scanning calorimetry. The interaction with calf thymus DNA (CT-DNA) was examined by ultraviolet (UV) absorption. Finally, a molecular docking study was performed using leishmanin (1LML), cruzain (4PI3), and *P. falciparum* alpha-tubulin as the ligand receptors.

## 2. Results and Discussion

### 2.1. Chemistry

**Scheme 1:**
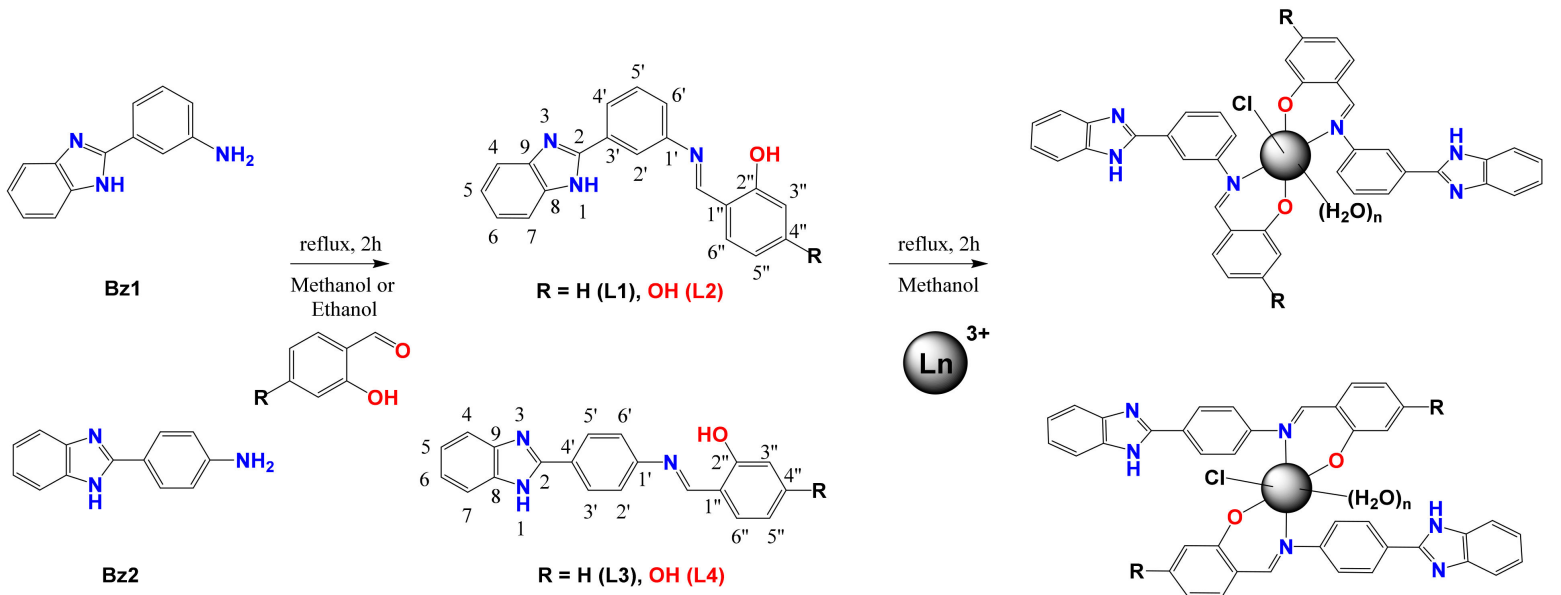
Synthesis of Schiff bases (ligands) and lanthanide complexes (Ln = La(III), Ce(III)).

The synthesis of the Schiff bases was carried out in two steps. Initially, aminophenyl benzimidazoles (Bz1 and Bz2) were obtained following the condensation of *o*-phenylenediamine with the corresponding carboxylic acid. Subsequently, imine formation was performed using salicylaldehyde or 2,4-dihydroxybenzaldehyde in ethanol or methanol (Scheme 1). These compounds were obtained as yellow powders, with moderate-to-high yields (>70%), and were used to obtain lanthanide the complexes (La-L1, Ce-L1, La-L2, Ce-L2, La-L3, Ce-L3, La-L4, Ce-L4) in methanol from their LnCl_3_·*n*H_2_O (Ln = La(III), Ce(III)) salts. The lanthanide complexes were also obtained in good yields (>65%). All compounds were characterized by mass spectrometry (MS), elemental analyses (C, H, and N), infrared (IR) spectroscopy, and nuclear magnetic resonance (NMR) spectroscopy. Additionally, the metal complexes were characterized by ethylenediaminetetraacetic acid (EDTA) complexometric titration, thermogravimetric analysis (TGA), and molar conductivity, which suggested the formation of structures with a metal:ligand ratio of 1:2 [21,29] and confirming the non-electrolytic nature of the resulting complexes [30].

#### Spectral Characteristics

The IR spectra of the synthesized compounds confirmed the formation of imines and the metal ion-binding sites. The formation of imines was confirmed by the absence of υ(N-H_2_) in Bz1 and Bz2 in the range of 3500–3300 cm^−1^, and the formation of a characteristic band for the azomethine group υ(N = CH) at approximately 1600 cm^−1^ [31]. In the IR spectra for the metal complexes, the υ(N = CH; 15–35 cm^−1^) vibration frequencies were shifted to higher wavenumbers, and a shift in the band for the azomethine group suggested the coordination of this group with the metal ion [32]. The phenolic C–OH stretching vibrations in the Schiff bases were unequivocally identified by the observation of intense bands in the range of 1280–1260 cm^−1^, and a signal present at 1570 cm^−1^ due to the υ(N = C) of the imidazole ring. For the lanthanide complexes, the phenolic C–O band shifted to a higher frequency, in the range of 1350–1300 cm^−1^, which demonstrated the coordination of the oxygen atom with the lanthanide ion, agreeing well with other reports on the coordination of these Schiff bases with transition metals [29,33].

^1^H NMR spectra show the characteristic signals of the different systems present in these Schiff bases [34,35,36]. At a low field, the signals corresponding to the protons of the imidazole and the -OH present in the salicylidene ring were observed. A signal that appeared near 9.0 ppm confirmed the formation of the imines, demonstrating the presence of the proton in -CH = N-. The remaining signals were observed in the typical region for aromatic protons (see Table S2). Characterization by ^13^C{^1^H} NMR afforded spectra where the signals for the carbons from -CH = N- and OH-C appeared between 165–160 ppm, which is typical for imines, whereas the imidazole CH = N signal was observed at approximately 151 ppm.

Both ^1^H and ^13^C{^1^H} NMR spectroscopy confirmed the binding of the ligands to the metal center. The ^1^H NΜR spectra of the La complexes did not exhibit the presence of the signal due to the -OH corresponding to position 2’’ of the salicylidene ring and the shifting of some signals in the aminophenyl and salicylidene rings, such as the down-field shifts of the azomethine proton from 9.10 ppm (L1) to 9.18 ppm (La-L1) and from 8.94 ppm (L2) to 9.01 ppm (La-L2). The ^13^C NMR spectra of the La complexes exhibited a small variation in chemical shifts for most signals; however, the azomethine carbon shifts from 163.69 ppm (L2) to 165.79 ppm (La-L2) and from 163.64 ppm (L4) to 165.83 ppm (La-L4) being these values similar to those observed for the analogous transition metal complexes [21].

### 2.2. Biological Studies

Due to the various pharmacological applications that have been reported for various Schiff bases derived from benzimidazoles [29,37,38,39], the preliminary in vitro evaluation of the above-mentioned species was studied by examining the antiproliferative, antiparasitic, and antibacterial properties of both ligands and their complexes.

#### 2.2.1. Antiproliferative Properties

**Figure 1:**
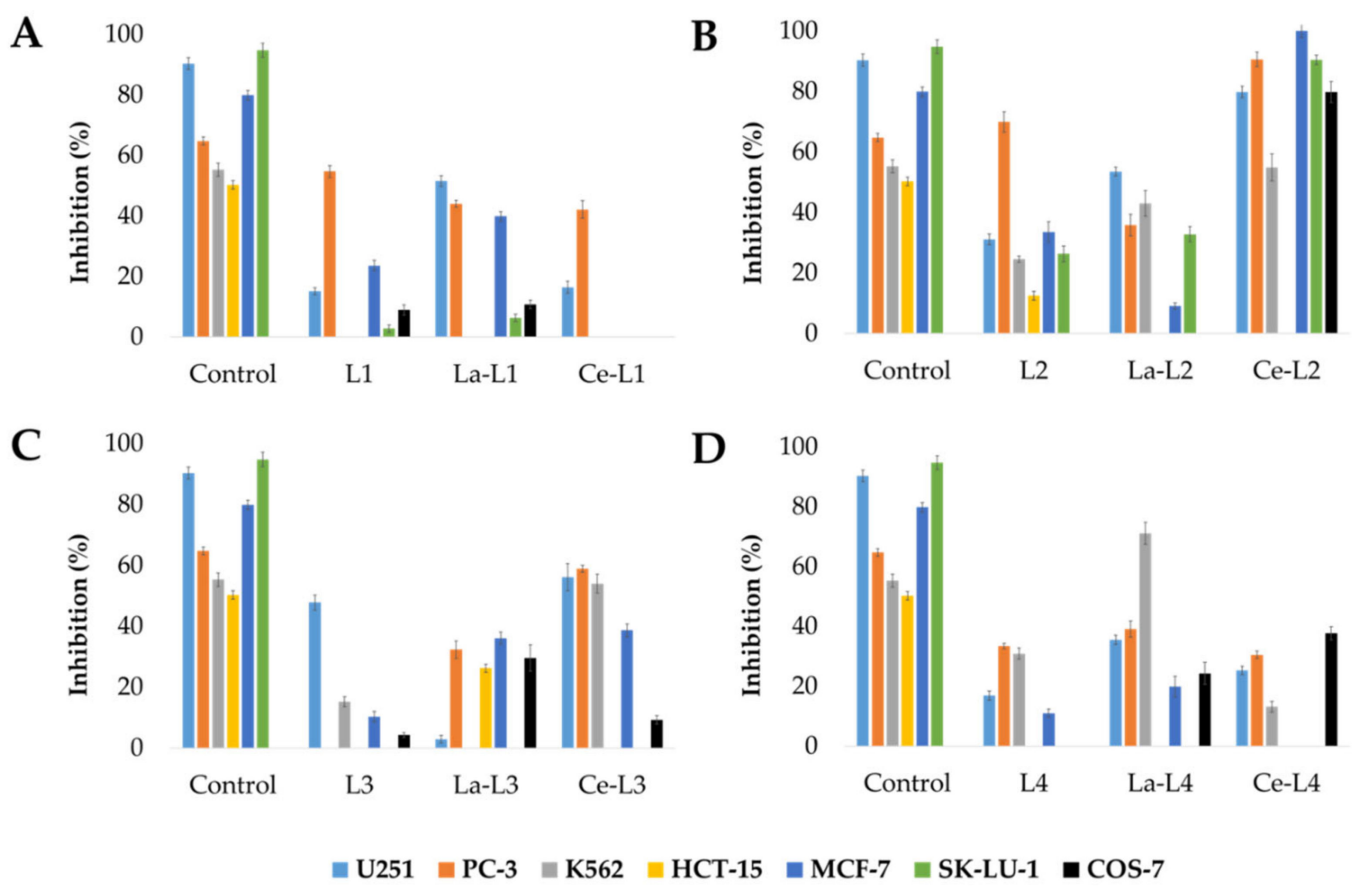
Inhibition growth (%) of different human cancer cell lines at 25 μM for (**A**) L1 series, (**B**) L2 series, (**C**) L3 series, and (**D**) L4 series; control = cisplatin. (Missing bars mean no activity was observed.).

The in vitro antiproliferative activity of both ligands and their corresponding complexes was determined by employing a series of human cancer cell lines (U251, PC-3, K562, HCT-15, MCF-7, and SK-LU-1) and one non-cancerous monkey cell line (COS-7). The results presented in Figure 1 show that these species inhibit the cell growth of U251, PC-3, and K562 cancer cell lines. In addition, the lanthanum and cerium complexes were generally more cytotoxic than the corresponding Schiff base ligands, proving the collaborative effect caused by the presence of the metallic centers.

When comparing the results of the L1 and L2 series, a greater inhibitory effect was observed for the Schiff bases prepared from 2,4-dihydroxybenzaldehyde, which indicates that having an -OH functional group at the *para-* position of the salicyldene ring is very relevant for the biological behavior of this compound. The outcome of the substitution in the aminophenyl ring was also observed when comparing the results of the L2 series with those of the L4 series, revealing increased inhibitory activity for the 1,3-position compared with the 1,4-position, similar to the effect observed for the position of the amino group in N,O-chelate ligands derived from benzimidazolyl-phenol [40].

The La-L4 complex was the only compound to exhibit a greater inhibitory effect on the examined cancerous cell lineK562 when compared with the reference drug (55.3 ± 2.2), whereas the Ce-L2 complex produced the highest inhibition results against U251 (79.8 ± 1.9), PC-3 (90.6 ± 2.4), MCF-7 (100.0 ± 2.2), and SK-LU- 1 (90.4 ± 1.5). Higher activities were observed for the Ce-L2 complexes in PC-3 and MCF-7 cells than those observed with the reference drug (64.8 ± 1.3 and 79.9 ± 1.6, respectively). However, a high percentage of inhibition observed against the non-cancerous cell line (COS-7, 79.8 ± 3.4) indicates that this compound has non-specific effects and should not be recommended for further studies.

Some potential targets that may be targeted by benzimidazole compounds can be involved in the underlying mechanism for this behavior, including the inhibition of Aurora A kinase, cyclin-dependent kinase (CDK), and dihydrofolate reductase [29]; however, the inhibition of heat shock proteins (Hsps) represents the most promising proposed mechanism for the future development of anticancer agents using the generated Schiff bases in the present study. L4 is known to be a potent and selective inhibitor of the molecular chaperone Hsp90, with important cytotoxic consequences versus carcinoma cells (PC-3) [36]. Additionally, the molecular docking studies discussed below indicated that the ligands could interact with molecular targets through hydrophilic bonds and conventional hydrogen bonds, in addition to pi-alkyl interactions with the three aromatic rings, which may also be the mechanisms involved in the inhibition of human DNA topoisomerase I [41]. Another selective target might be phosphatidylserine (anionic phospholipid), which is found on the outer membrane of cancerous cells and may electrostatically attract the positively charged ligand when in keto-enolic equilibria [40], as occurs for salen-type Schiff bases.

#### 2.2.2. Antiparasitic Properties

The in vitro antiparasitic activities of the series of ligands of complexes were examined against *L. braziliensis*, *P. falciparum,* and *T. cruzi* (Table 1). Cytotoxicity was initially assessed using the human promonocytic cellular line (U-937), exhibiting moderate to high cytotoxicity for all the series of compounds [42]. It is worth mentioning that cytotoxic studies were performed on erythrocytes, before the evaluation against anti-*P. falciparum*, obtaining LC_50_ values >200 μg/mL in all cases (compounds and control).

The in vitro leishmanicidal activity results showed that, in most cases, leishmanicidal activity was similar to cytotoxicity, and the La-L2 complex displayed a high level of activity against *L. braziliensis*, thus implying the activity is dependent on both the type of the ligand and metallic center. This result for La-L2 was further supported by the molecular docking results performed with the leishmanin receptor, for which hydrophobic interactions were primarily observed. The results of trypanocidal activity clearly show that the Schiff bases have moderate to high activity [42]. For antiplasmodial activity, all the examined compounds altered the parasites’ respiratory process, effectively reducing the infection rate by at least 50% at the studied concentration. As the effective concentration was less than 20 µg/mL, these can be considered highly active compounds [42]. The antiparasitic activities observed could be related to the abilities of these compounds to form hydrophobic interactions through the benzene rings, as demonstrated in the molecular docking studies for cruzain and alpha-tubulin. The high hydrophobic nature of Schiff bases, containing various benzene rings, has been shown to increase their lipophilic properties and facilitate their movements across the membrane bilayer [43]. Other possible routes of action for the biological activities observed for Schiff bases containing benzimidazole include interactions with DNA [15]. Studies have shown that DNA-binding ability is possible through the lone pair of electrons of the nitrogen of the imine group, found at the *sp*^2^ orbital [44].

#### 2.2.3. Antibacterial Properties

**Figure 2:**
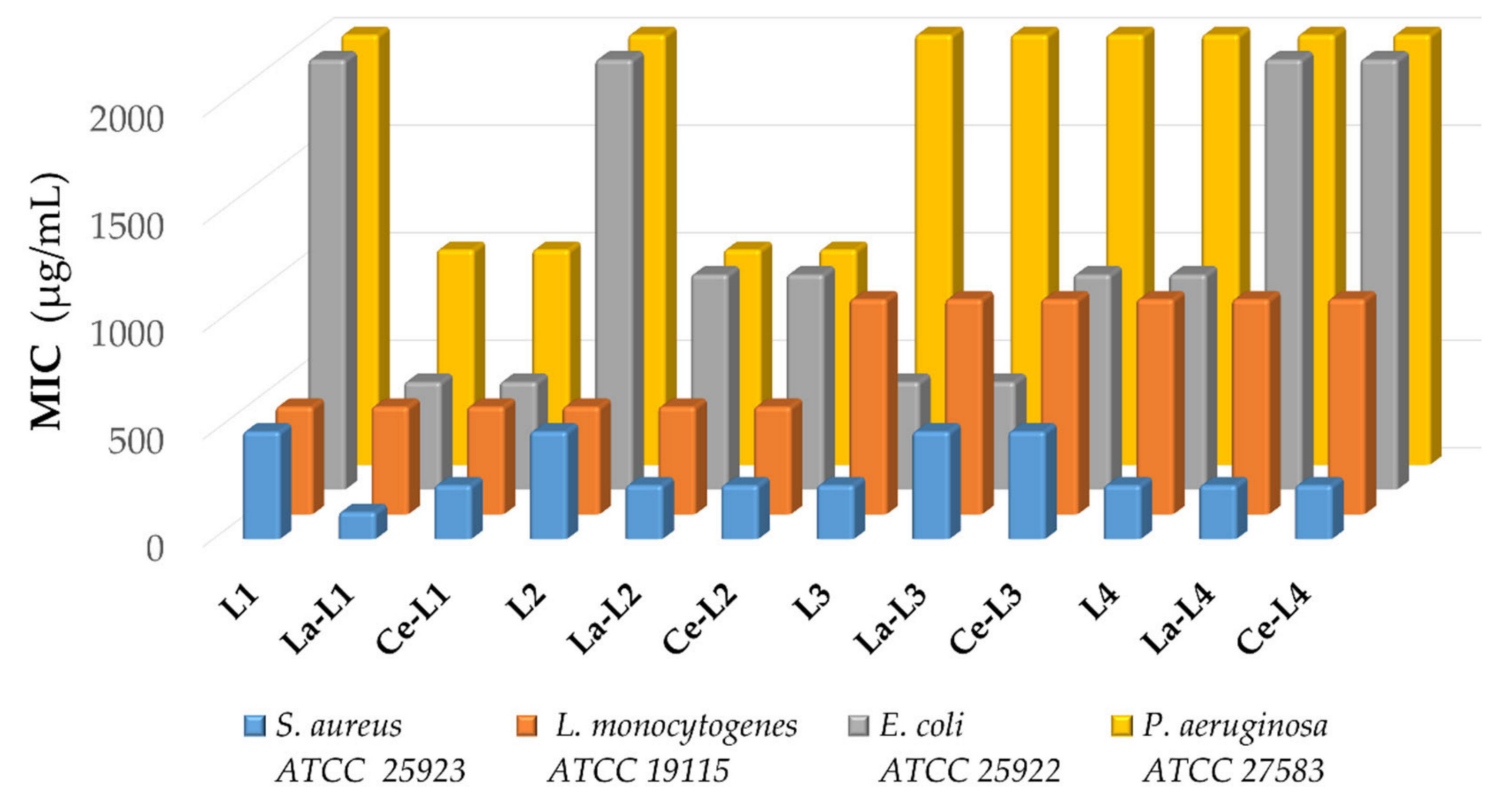
Minimal inhibitory concentrations (MICs) determined for the series of compounds on the antibacterial assays (µg/mL); controls: ciprofloxacin and silver nitrate.

The in vitro antibacterial activities of both the ligands and complexes were assessed against Gram-positive (*S. aureus* ATCC^®^ 25923, *L. monocytogenes* ATCC^®^ 19115) and Gram-negative bacterial strains (*E. coli* ATCC^®^ 25922, *P. aeruginosa* ATCC^®^ 27583) employing broth microdilution assays. Figure 2 and Table 2 show results to be dependent on the bacterial type evaluated, with all tested compounds generally showing better effects versus Gram-positive than versus Gram-negative bacteria because the outer membrane plays the role of a permeability barrier, preventing some antibiotics from reaching their targets [45]. Additionally, these results suggested that the compounds may exert their antibacterial activities through interactions with phospholipids found on the bacterial cell membranes [39], as confirmed by the studies performed on membrane models, which will be discussed in a later section.

The low levels of antibacterial activity observed for the evaluated compounds against *P. aeruginosa* were attributed to the high level of resistance that this strain has demonstrated previously against various antibiotics [46]; however, the antibacterial performances of both ligands L1 and L2 improved when complexed with the metal ions. A similar effect was observed against the *S.* *aureus* strain, for which the lanthanum and cerium complexes coordinated with L1 and L2 showed greater activity than the free ligands. In general, L1 and L2 showed lower minimum inhibitory concentrations (MICs), in both the free and complexed forms, than L3 and L4, which suggest an effect caused by the aminophenyl ring substitution that is favored by the 1,3-position. This outcome is also supported by the molecular docking results for the methicillin-resistant *S. aureus* PBP2A protein, for which the binding energies suggest a better affinity for L1 and L2 than for L3 and L4 (see Table S6). Interestingly, for the analysis of antibacterial activity against the *S. aureus* strain, the presence of the metal appeared to decrease the bacteriostatic effect compared with the free L3 ligand, and the complex La-L1 was the only tested compound to exhibit a lower value (125 µg/mL). La-L1 also presented the best binding energy with the *S. aureus* PBP2A protein, mediated by the formation of multiple hydrophobic interactions according to molecular docking studies. Other lanthanide complexes formed with Schiff bases have also shown better antibacterial performances versus *S. aureus* [24], and the potential mechanism of such action might involve interactions with the bacterial membrane or interactions with DNA [21].

### 2.3. Interaction with Potential Molecular Targets

In the development of small-molecule probes and drug discovery, the identification of the target and potential action mechanism are of fundamental relevance [47]. The identification of molecular targets primarily involves three complementary approaches that involve direct biochemical methods, genetic interaction methods, and computational inference methods. Therefore, in this work, we examined (1) physical interactions using direct methods, such as interactions with synthetic membrane models; (2) interactions between the compounds and DNA; and (3) computational interaction studies, including molecular docking and molecular dynamics, using receptor proteins that are commonly found in the organisms that were examined in the in vitro activity tests.

#### 2.3.1. Interaction with Synthetic Model Membranes

The membrane models included mammalian-like membranes consisting of the phospholipid 1,2-dimyristoyl-sn-glycero-3-phosphocholine (DMPC) and bacterial-like membranes consisting of a 3:1 ratio of DMPC: dimyristoyl phosphatidylglycerol (DMPG). Each membrane model exhibited two endothermic peaks, one pre-transition peak, and one main transition peak. The pre-transition peaks occurred at 12.94 and 12.71 °C for DMPC and the DMPC:DMPG mixture, respectively, whereas the transition temperature when the mixture transformed from a gel to a crystalline liquid (*Tm*) for both lipid systems was 23.02 °C (Table 3).

**Figure 3:**
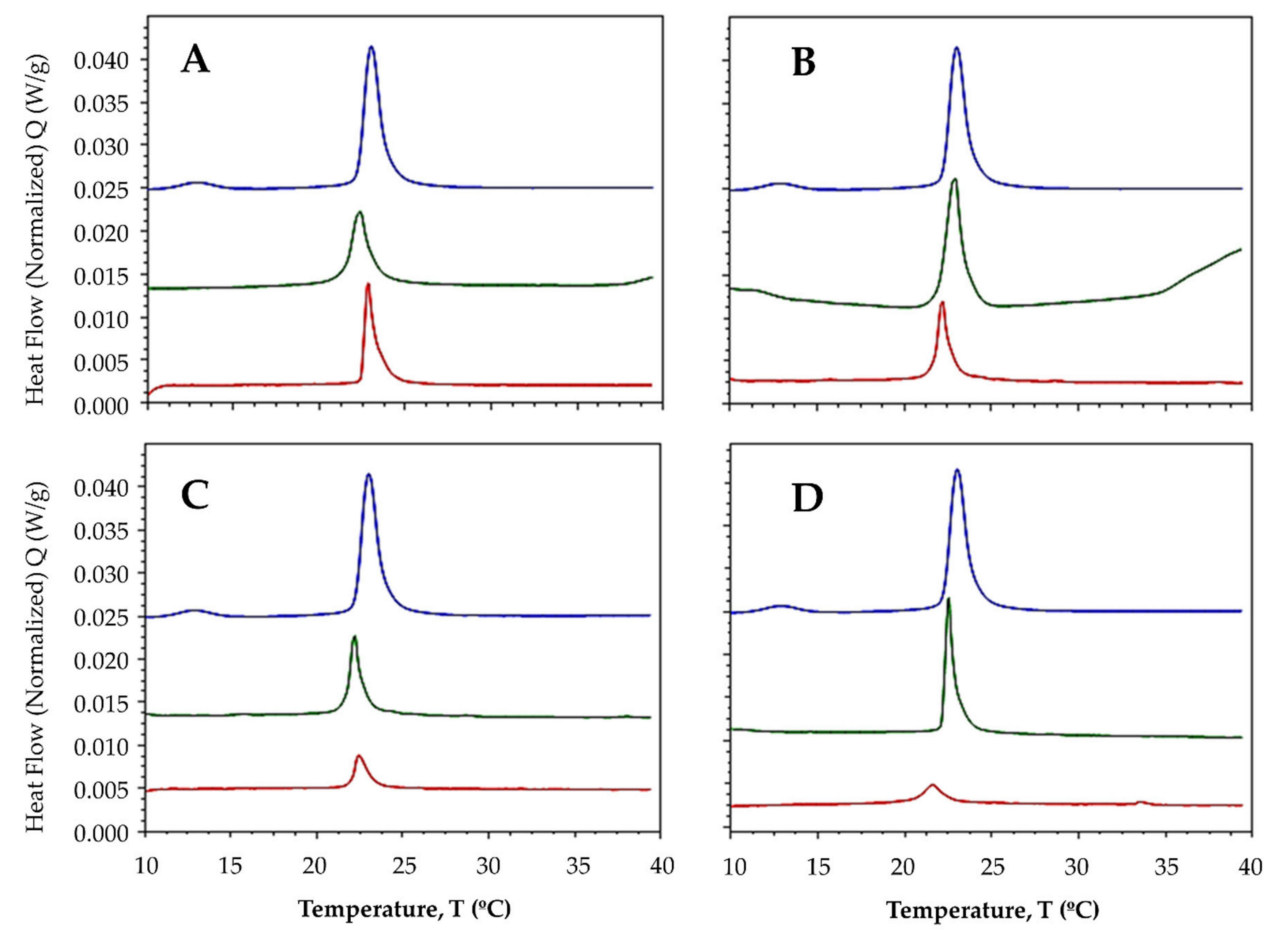
Thermotropic profile of MLVs made up of DMPC before (**----**) and after adding the ligand (**----**) and the La complex (**----**) in a 1:50 compound-lipid molar ratio. (**A**) L1; (**B**) L2; (**C**) L3; and (**D**) L4.

After the addition of ligands and complexes to both lipid systems at a 1:50 compound:lipid molar ratio, the pre-transition peak was completely abolished, suggesting that both free ligands and metal-bound complexes have the ability to disrupt the organization of membranes [48]. In multilamellar vesicles (MLVs) made up of DMPC lipids, the *Tm* was modestly reduced after ligands and complexes were added, shifting the main transition peaks to the left (Figure 3) and suggesting that the compounds may increase the fluidity of the membrane in response to the alteration of the hydrophobic bilayer core. Fluidity is known to increase in response to an increase in the lateral diffusion rates of lipid molecules [49].

In a previous study, we demonstrated that compounds derived from benzimidazoles have the ability to slightly fluidize membrane models [40]. The increased fluidity may be accompanied by an increase in permeability [50], which could be responsible for the antiproliferative effects exerted by these compounds. Some studies have shown correlations between different antitumor agents and membrane fluidity [51,52]. To understand the reactivity of ligands with DMPC, phosphatidylcholine must be considered to act as a hydrogen bond acceptor. Additionally, the hydroxyl groups in phenols could interact with the water molecules embedded within the membrane, whereas the hydrophobic moiety of the aromatic rings could be oriented toward the hydrophobic bilayer core. For ligands L1 and L3, the *Tm* increased slightly after the addition of the lanthanide ion (Figure 3A,C), whereas for ligands L2 and L4, the *Tm* decreased (Figure 3B,D).

Zschornig et al. [53] reported that the interactions of metals with the polar heads of phospholipids affect the membrane packing. Le et al. [54] reported that the headgroup zone of zwitterionic phosphatidylcholine could be penetrated by cations, coordinating with Lewis bases in line with their hardness, rather than via Coulombic interactions, where the contribution to the binding energy of lipid-lipid interactions is observed as a change in the phase transition temperature. By contrast, the transition enthalpy decreased considerably when ligands and lanthanide were added (Table 3), indicating an alteration of the interactions between the acyl chains of the lipids due to disrupted intra- and intermolecular van der Waals interactions and trans-gauche isomerization [55].

**Figure 4:**
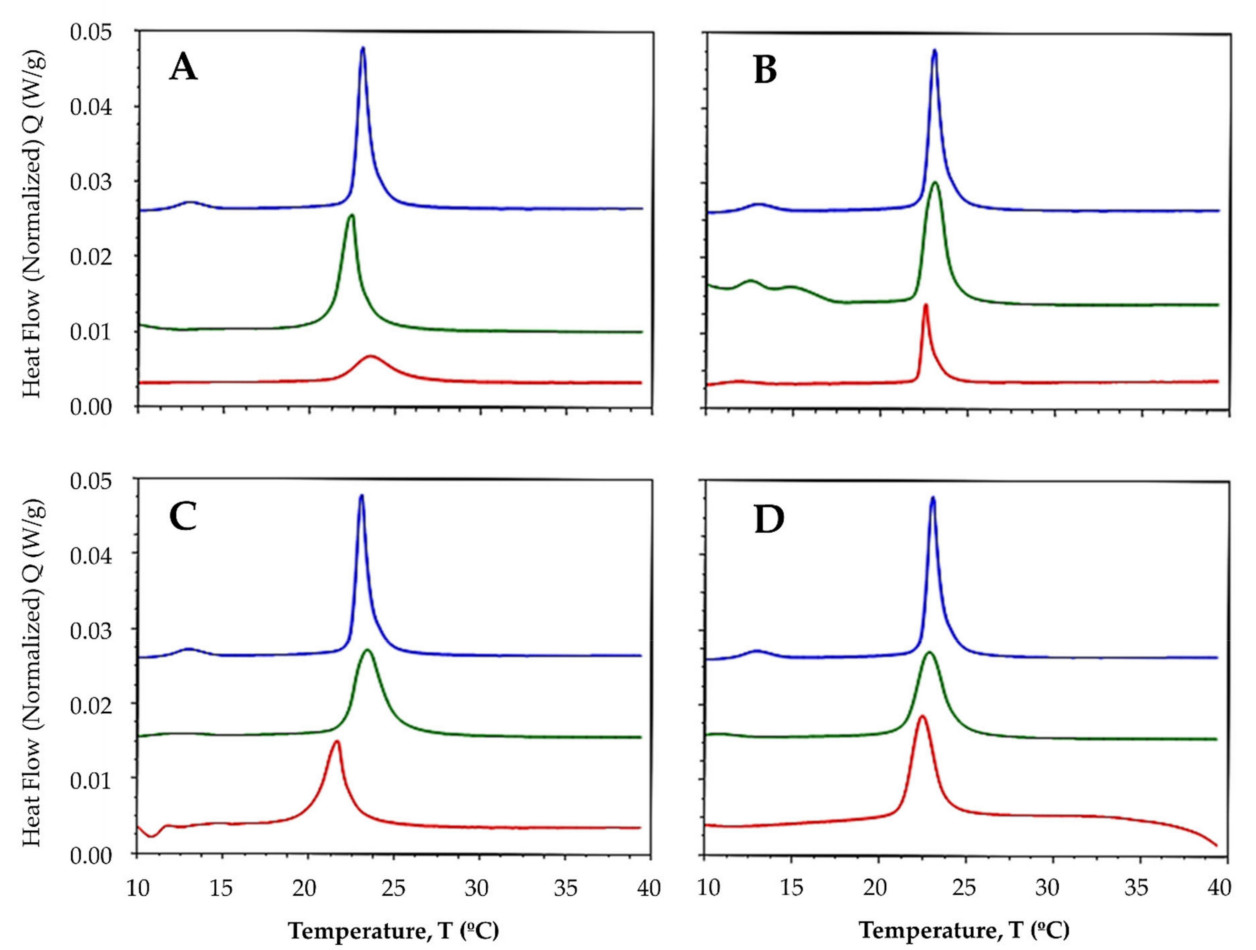
DCS thermograms from MLVs made up of DMPC/DMPG mixture before (**----**) and after adding the ligand (**----**) and the La complex (**----**) in a 1:50 compound-lipid molar ratio. (**A**) L1; (**B**) L2; (**C**) L3; and (**D**) L4.

In the membranes consisting of the DMPC:DMPG mixture, the *Tm* was modestly reduced after the addition of ligands and lanthanide (Figure 4). Interestingly, enthalpy decreased considerably only after the addition of the La-L1 and La-L2 complexes (Table 3), suggesting that the addition of anionic phospholipids alters the effects of ligands and complexes. These results could explain the moderate antibacterial activity shown by these compounds against Gram-negative bacteria and the aminophenyl ring-dependent structure/activity relationship. However, the effects of La-L3 and of La-L4 on the transition enthalpy were clearly stronger on DMPC membranes than on DMPC:DMPG membranes, which could be explained by the double-layer model, which describes the association of ions using the Stern equation [56], in which the ions of the compounds respond to the total anionic charge of the membrane; thus, the compounds do not penetrate the membrane but remain on the surface [53], preventing their insertions into the anionic bilayer where they can achieve a stronger effect, such as that which occurs for DMPC membranes.

#### 2.3.2. DNA Interaction by Electronic Absorption Monitoring

**Figure 5:**
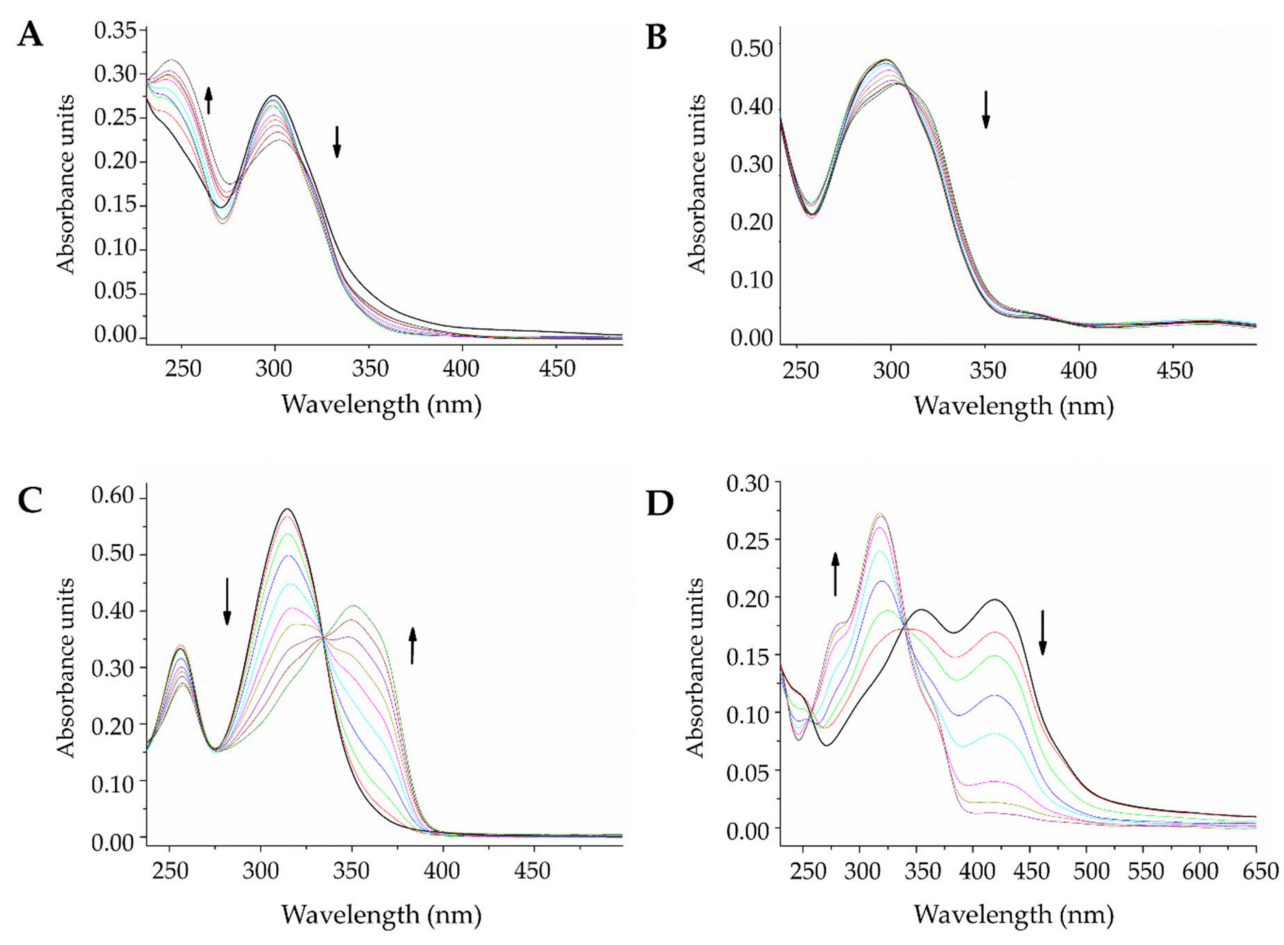
Photometric study at 60 µM of (**A**) L1, (**B**) L2, (**C**) L3, and (**D**) L4, with CT-DNA at concentrations between 0 and 96 µM (solid black line = 0 µM).

The interactions between small molecules and DNA strands represent an important potential mechanism of action for chemotherapeutic agents, which can affect genomic processes. UV/V spectrophotometry can be used to provide a general sense of the types of interactions that occur between nucleic acids and new compounds [57,58]. The electronic spectra of DNA, ligands, and complexes were followed through photo-titration, and the changes were observed as more titrant was added. Figure 5 shows the effects of DNA addition on the electronic spectra when the ligands are maintained at a constant concentration. In all spectra, hypochromism and bathochromism were observed in the lower energy bands (type π → π*), highlighting that a more considerable shift occurred for L3 and L4 than for the other tested compounds. The existing isosbestic points denoted a single mode of interaction between the double-stranded DNA and the free ligands.

**Scheme 2:**
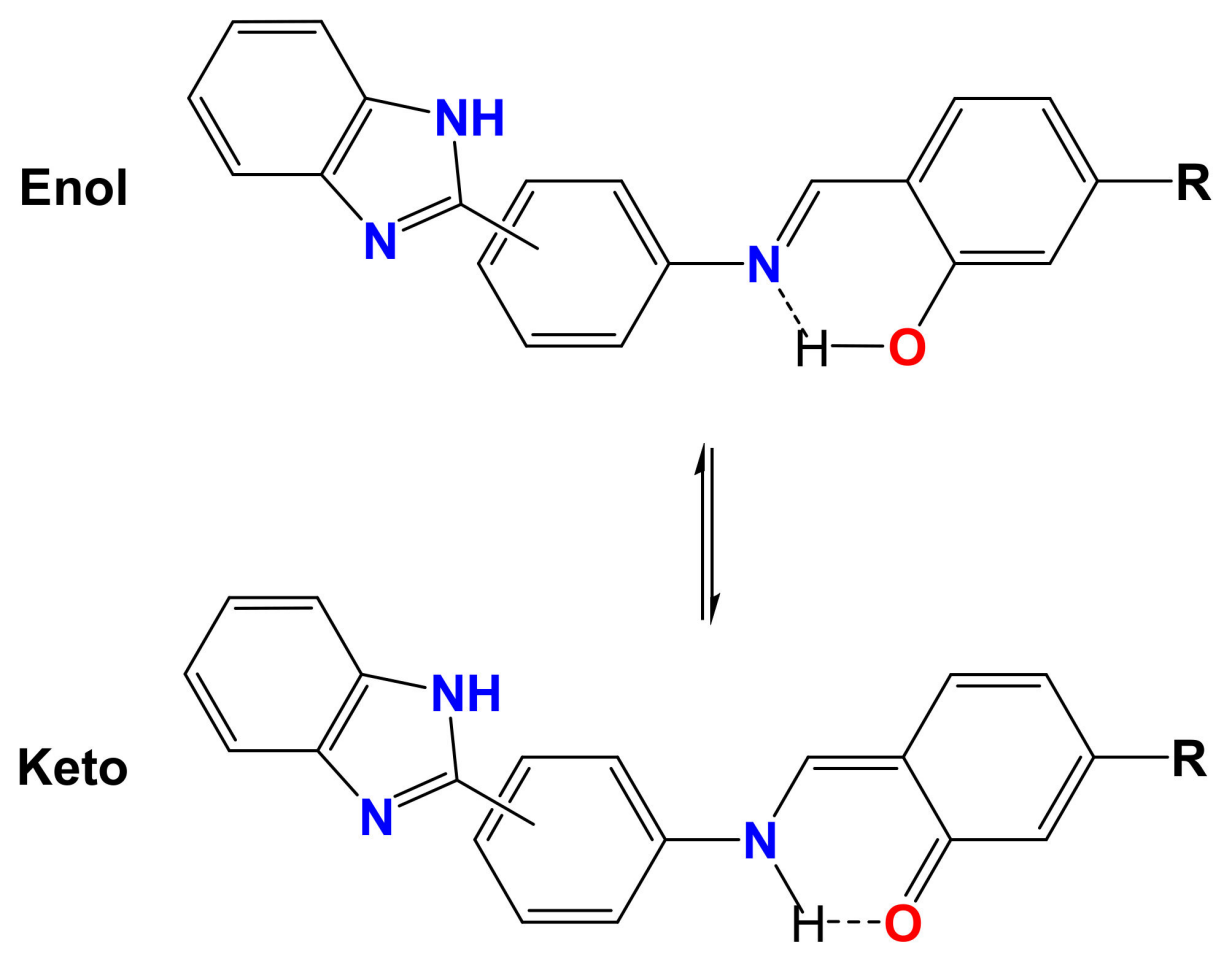
Keto-enol equilibrium of ligands (R = H for L1 and L3, OH for L2 and L4).

Salen-type Schiff bases can be present as various tautomers, as shown in Scheme 2. These structures present absorptions at different wavelengths, with generally lower energy levels for keto forms [59]. Two absorption bands can be observed in the L3 spectrum (Figure 5C), one at approximately 256 nm and the other at 315 nm, and the highest energy band is characteristic of the enol form. In contrast, for L4 (Figure 5D), two bands can be observed at 354 nm and 420 nm, and the latter was assigned to the absorption of the keto form [59], which indicates that for L4, both species coexist in free forms.

The presence of DNA in these systems would result in the stabilization of one of the tautomers when a DNA-compound complex is formed. For L3, as DNA was added to the system, the 315 nm band decreased, and a new band emerged at 350 nm, which indicated that in this system, the interaction with DNA could favor the keto form of L3. By contrast, for L4, the 420 nm band decreased, and the 354 nm band increased, with a hypsochromic shift of up to approximately 318 nm, which indicated that the union with DNA strands stabilized the enol form. For L1 and L2 (Figure 5A,B), the substitution in the *meta* position to the nitrogen of the imine bond appeared to favor the enol form for both compounds (-H or -OH), which remained stable even in the presence of DNA, resulting in only hypochromic and bathochromic effects after titration, without the appearance of new bands.

The lanthanide complexes followed similar patterns as the ligands (see Figure S37); however, the cerium compounds appeared to be stabilized in the enol form, without the emergence of new bands after titration (see Figure S38). The enol forms of the studied compounds are also the co-planar forms, which favor the intercalation of the compounds between the nitrogenous nucleic acid bases, which might be responsible for the observed changes at all degrees [21,58,60].

Monitoring the electronic spectrum of DNA (Figures S39–S41) also offered evidence of intercalation, revealing a hypochromic effect on the absorption band at 260 nm for molecules whose initial tautomeric shape was not changed by the interaction, such as the L1 and L2 compounds. The rest of the changes observed on the DNA spectrum were likely due to structural changes in the binding molecule that are different from the photometric target (binding molecule without change), which explains the negative absorbances and the baseline lags.

Using the Wolfe–Shimmer Equation (1), intrinsic binding constants (Kb) were estimated for evaluated compounds, considering the information obtained from the photometric titration spectra.

(1) $$\frac{\left[DNA\right]}{\varepsilon_{a}-\varepsilon_{f}}=\frac{\left[DNA\right]}{\varepsilon_{b}-\varepsilon_{f}}+\frac{1}{K_{b}\left(\varepsilon_{b}-\varepsilon_{f}\right)]}\;$$

where [DNA] stands for the concentration of added DNA, *ε_a_* corresponds to the apparent molar extinction coefficient, *ε_f_* refers to the molar extinction coefficient of the free compound, and *ε_b_* represents the molar extinction coefficient in DNA saturation [58]. Figures S42–S44 show the linear adjustments made to calculate the binding constants, which are summarized in Table 4.

In general, the binding constants for lanthanide complexes were larger than those for the free ligands. The presence of the positive metal center likely favors electrostatic interactions with the negative DNA groove, resulting in a type of insertion interaction involving both intercalative and sulcus-binding mechanisms [61]. The highest constants were identified for the Ce-L3 and Ce-L4 complexes, for which the stabilization of the co-planar enol form facilitated π stacking between nitrogenous bases. The constants were recorded on the order of 10^4^ M^−1^, which were two orders of magnitude lower than those identified for classic markers and intercalators, such as ethidium bromide or propidium bromide, which feature constants between 10^6^ and 10^8^ [57,58].

Thus, all of the evaluated compounds could interact with DNA strands through intercalative and insertion junctions. Although the proposed tautomeric changes must be supported using additional studies, the formation of the DNA-compound complex can favor multiple forms. The chemical composition of the nitrogenous bases of DNA in eukaryotes is identical to that of prokaryotes; hence, the compound could also bind to bacterial DNA, inhibiting replication [62] and thus cell division. Benzimidazole derivatives have been described as being able to bind to the minor groove of DNA, leading to the formation of single-strand DNA breakage [63], possibly leading to the arrest of the cell cycle [64]. In this way, the inhibition of bacterial proliferation can prevent the progression of infectious foci inside the host.

#### 2.3.3. Molecular Docking

**Figure 6:**
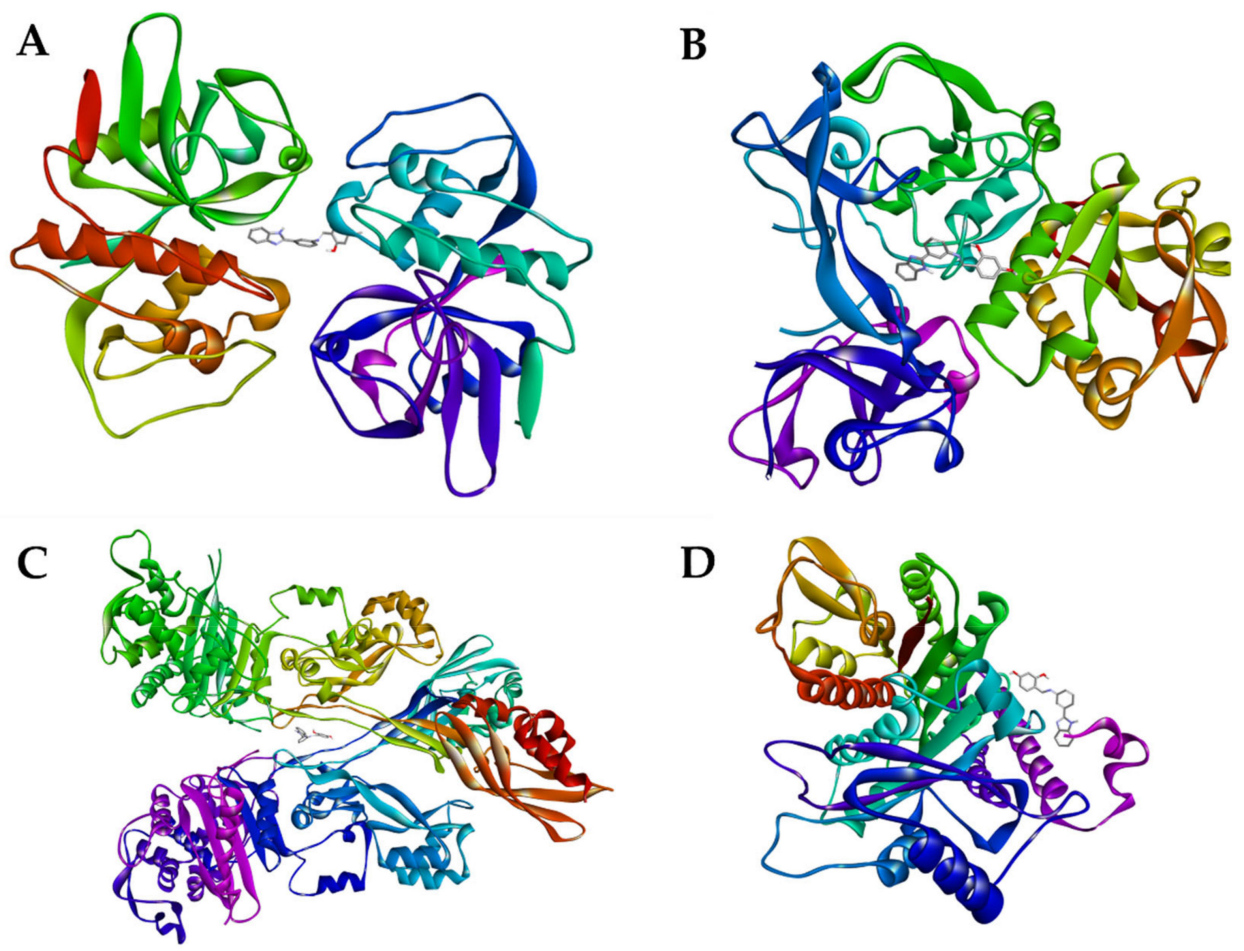
Receptors with L2 ligand: (**A**) Cruzain; (**B**) Leishmanin; (**C**) PBP2A; (**D**) Alpha-tubulin.

Figure 6 shows the four proteins that were used as receptors for the ligands and complexes in the molecular docking experiment. The coordinates used to locate the ligands, complexes, and controls were the same for each receptor protein and were obtained with CB-DOCK by applying blind docking predictions to the binding sites of the target proteins using cavity detection, based on the curvature (CurPocket) and binding positions of the ligands, using AutoDock Vina [65]. For cruzain, the grid center was (18,204, 7728, −20,409); for leishmanin, the grid center was (11,856, 39,761, 15,011); for alpha-tubulin, the grid center was (73,153, 57,344, 66,622); and for PBP2A, the grid center was (17.0, −21.0, −54.0). Table S3 shows the different molecules used to examine docking with cruzain, their corresponding binding energies, and their interactions. Comparing the ligands with each other, the ligand with the best affinity for cruzain was L4, which showed the lowest binding energy of 7.96 kcal/mol. When L4 was compared with L1, differences were observed in the number of interactions, with L4 featuring four hydrophobic interactions, which is one more than for L1. However, when L3 was compared with L4, the numbers of interactions were very similar, as illustrated in Figure S45; therefore, their binding energies were also similar. L2 appeared to present the largest number of hydrophobic interactions toward its benzene rings.

The interactions observed between the tested complexes and cruzain were better than those observed for the ligands, with the lowest energy observed for the La-L1 complex at −11.58 kcal/mol. The La-L1 complex presents 10 intramolecular interactions, five of which were due to the presence of the metallic center. Lanthanum only forms coordinated bonds with nitrogen or oxygen atoms with the ligands of the complex and not with atoms of the receptor. However, the numbers of interactions with cruzain were the lowest compared with those of the other molecules, forming only two hydrogen bridges with SER64 and LEU160. SER64 was also involved in the formation of hydrogen bridges with La-L1, Ce-L1, La-L2, and Ce-L2, which indicated that SER64 is important for the interaction between cruzain and the ligands that present an *m*-aminophenyl position in the active center. Another residue that was involved in interactions at a high frequency was ASP161, which formed hydrogen bridges, electrostatic interactions, and hydrophobic interactions with both ligands and complexes. Vinylsulfone had the fewest interaction compared with the binding energy results of the ligands and complexes.

In Table S4, the interactions between the ligand 2, La-L2 complex, and the amphotericin B control are reported for leishmanin. Amphotericin B had the lowest affinity for leishmanin, with a binding energy of -6.80 kcal/mol compared with −8.78 kcal/mol for ligand 4 and −9.79 kcal/mol for the La-L2 complex. Amphotericin B forms seven hydrogen bridges with leishmanin. Both L2 and the La-L2 complex present interactions with leishmanin that are primarily hydrophobic, and both interact with the ALA348 and PRO460 residues. The ALA349 residue was shared between L2 and La-L2.

**Figure 7:**
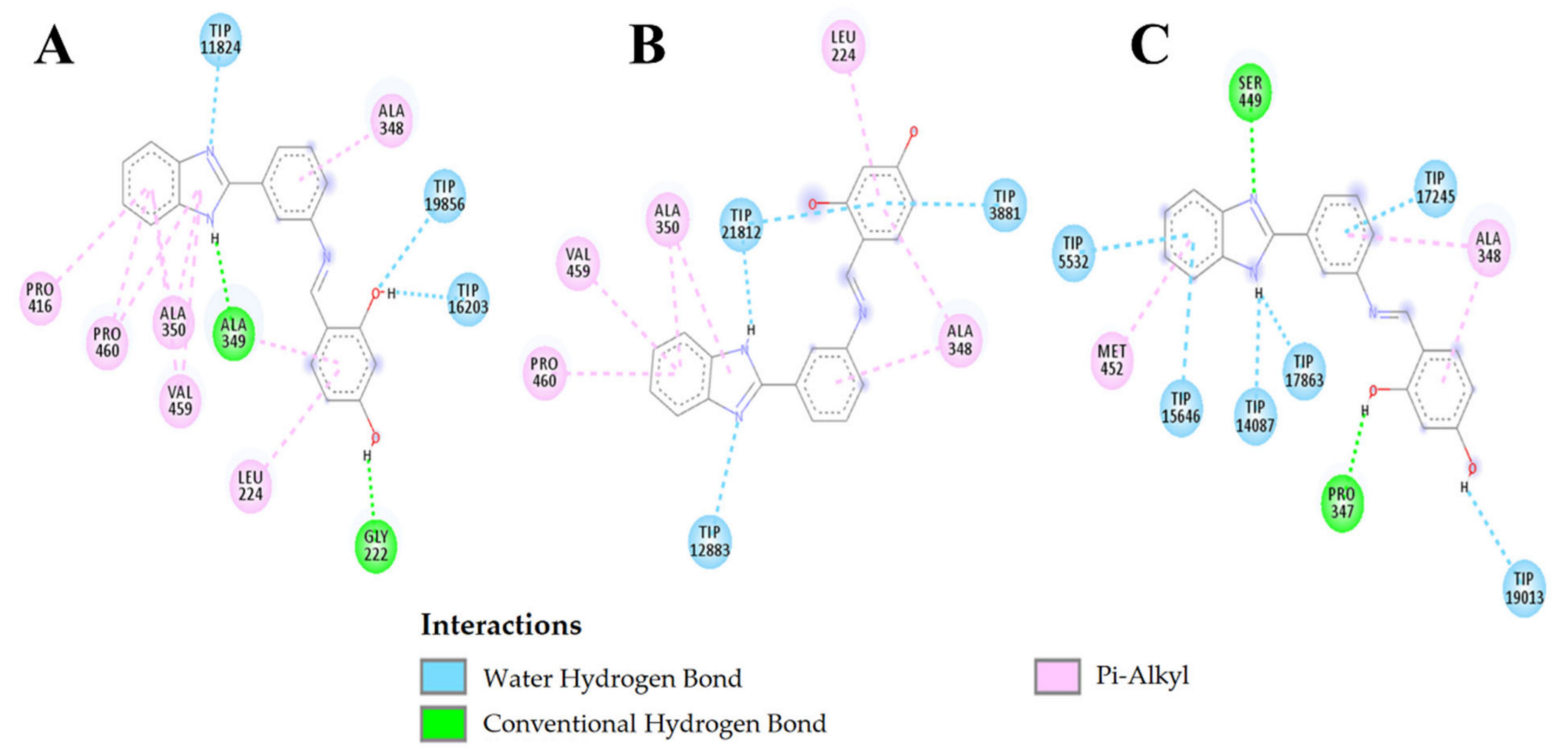
Interaction of the molecular dynamics between L2 and leishmanin. (**A**) at 0 ns; (**B**) at 5 ns; (**C**) at 10 ns. TIP: Water.

Figure 7 shows L2 interaction with leishmanin over time. At 0 ns, three hydrophilic interactions with water were observed, that remained constant in number until 5 ns, being duplicated at 10 ns. In contrast, the hydrophobic interactions proceed from six residues of leishmanin at 0 ns to only two residues at 10 ns. The ALA348 residue seems to interact during the whole simulation with the benzene rings; thus, we believe that it is an important residue in the active site of leishmanin.

**Figure 8:**
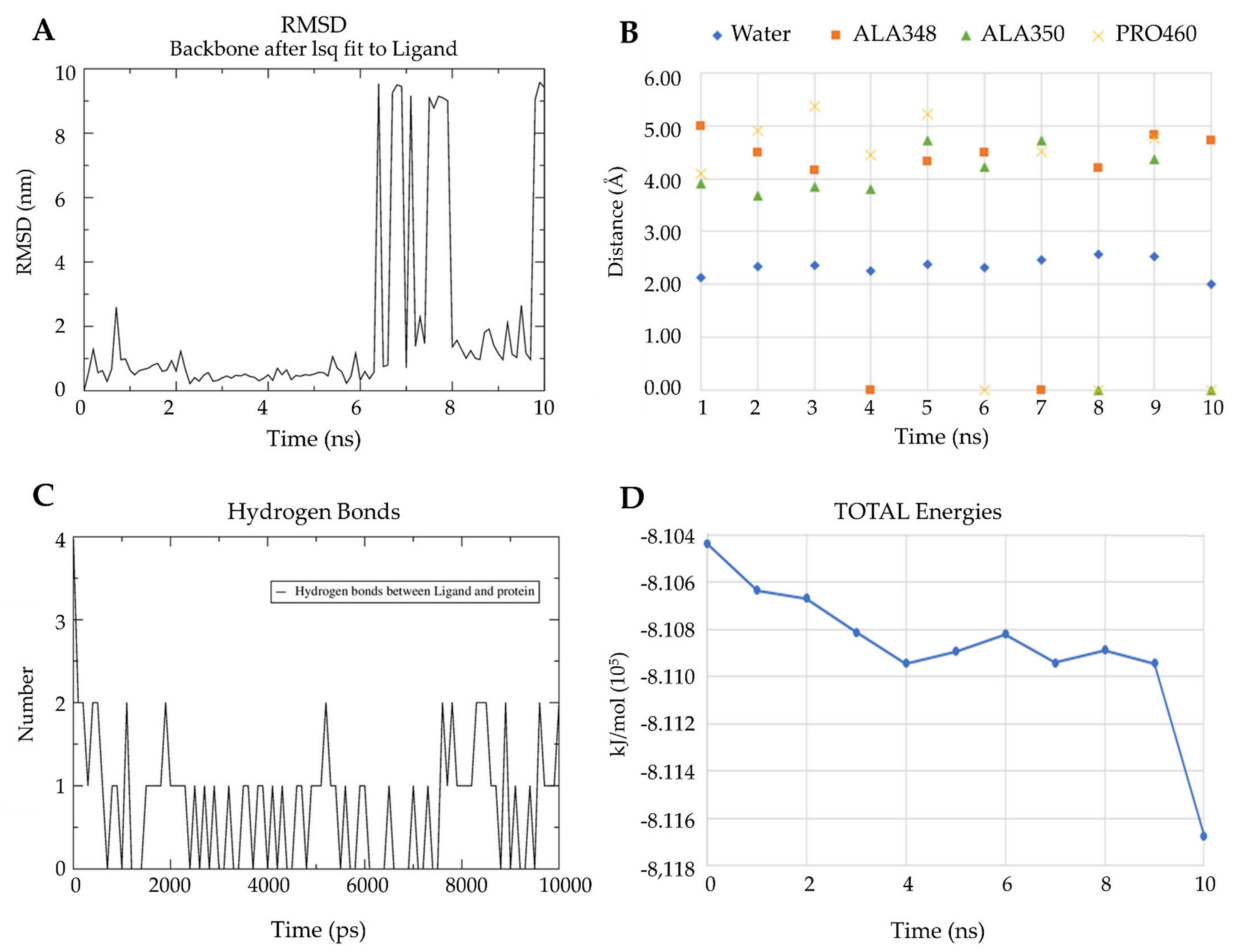
Results of molecular dynamics studies for L2: (**A**) RMSD between ligand with leishmanin; (**B**) Distance between ligand, water, and leishmanin residues ALA348, ALA350, PRO460; (**C**) Hydrogen bonds between ligand and leishmanin; (**D**) Total energy between ligand and leishmanin.

Figure 8A shows the root-mean-square deviation (RMSD) for the L2 structure variations with respect to its interaction with leishmanin. Low variation in the L2 structure was observed during the first 6 ns; however, between 6 and 8 ns, the behavior changes. Between 8 and 9 ns, the variation decreases, but starting from 9 ns, high variation is observed again. Figure 8B shows the distances in Angstrom (Å) between the ligand and the water molecules, as well as residues ALA348, ALA350, and PRO460 of leishmanin. The distance to water occurred during the 10 ns with an average of 2.5 Å. Residues ALA348 and ALA350 had between 4 and 5 Å distance to L2. ALA348 at 4 and 7 ns lost interaction with L2, as did ALA350 at 8 and 10 ns. Residue PRO460 had the longest distance to L2 (4-6 Å) and its interactions were constant for 10 ns except at 6 ns.

The hydrogen bonds between ALA348 and L2 are shown in Figure 8C. These findings confirm the importance of this residue in the interaction with L2. In Figure 8D, a decrease in total energy is observed up to 4 ns, reaching −811,000 kJ/mol. Then, it goes up to 6 ns to −810,800 kJ/mol and decreases at 7 ns again to −811,000 kJ/mol. Finally, at 9 ns, it drops to −811,600 kJ/mol, which correlates with a stronger interaction between L2 and the ALA348 residue, forming up to three hydrophobic interactions.

**Figure 9:**
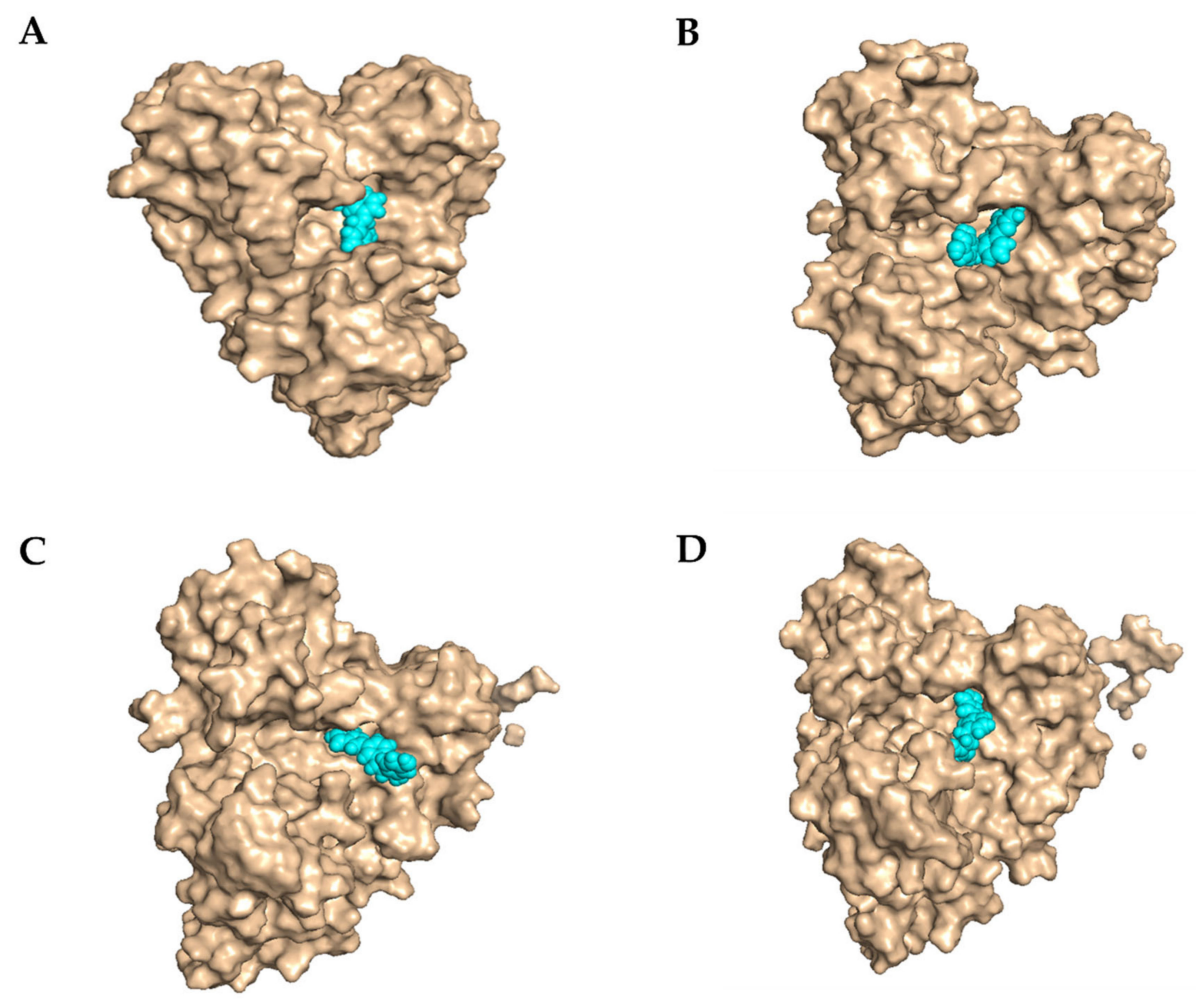
Surface of leishmanin with L2 (**A**) at 0 ns. (**B**) at 6 ns;(**C**) at 8 ns; (**D**) at 10 ns.

In Figure 9, L2 appears to remain in the pocket most of the time, and only at 8 ns a distance was observed, which coincided with the calculated distance and the RMSD plot.

In the interactions observed for the tested molecules and *P. falciparum* alpha-tubulin, as shown in Table S5, the vinblastine control had the best interaction characteristics, with a binding energy of −8.89 kcal/mol, followed by the La-L3 complex at −8.60 kcal/mol. The ALA451 and PRO263 residues generated both hydrogen bridges and hydrophobic interactions with the ligands and complexes.

Table S6 shows the interactions and binding energies between methicillin-resistant *S. aureus* PBP2A protein and the tested ligands, complexes, and controls. Ceftobiprole was the antibiotic with the best affinity, at −7.06 kcal/mol, which presented a better affinity than L3 and L4. The La-L1 complex presented the best binding energy at −10.01 kcal/mol, with a high degree of hydrophobic interactions, particularly with VAL277. VAL277 is present in all hydrophobic interactions with the benzene rings in the lanthanum and cerium complexes. ARG151 is another important residue for the interactions with the tested complexes, either forming hydrogen bridges or hydrophobic interactions. The most frequent hydrophobic interactions identified between ligands and PBP2A are LEU155, ASP323, and LYS322. Other PBP2A residues that were frequently found to interact with the complexes were LYS148, HIS293, and ASP295.

## 3. Materials and Methods

### 3.1. Materials and Physical Measurements

The commercially acquired chemical reagents were used directly without prior purification. Elemental analysis of hydrogen, carbon, and nitrogen was performed in a Flash EA 1112 Series CHN Analyzer EAE (Thermo Fischer Scientific, Waltham, MA, USA). Lanthanide concentrations were determined by complexometric titration. The conductivity of the metal complexes was determined in ethanol (1 × 10^−3^ M) using an Orion^TM^ 131S (Thermo Fischer Scientific, USA). Melting points were recorded on a Mel-Temp II apparatus (Mettler Toledo, Columbus, OH, USA) and are reported without correction. The IR spectra were recorded on a Shimadzu Affinity 1 (FT-IR) spectrometer (Shimadzu, Miyazaki, Japan). ^1^H and ^13^C{^1^H} NMR spectra were obtained at 25 °C using DMSO-*d*_6_ as a solvent on a Bruker Avance II 400 spectrometer (Bruker Corporation, Billerica, MA, USA). NMR spectra were recorded in δ units relative to deuterated solvent as an internal reference. The following abbreviations were used: s = singlet; d = doublet; t = triplet; m = multiplet. The thermal analysis was performed on the TGA 550 analyzer (TA Instruments, New Castle, DE, USA) under nitrogen atmosphere between 50 and 500 °C, with a heating rate of 10 °C/min. Electronic impact (EI) ionization mass spectra of the pre-ligands and two ligands were recorded on a Shimadzu-GCMS-QP2010 (Shimadzu Corporation, Miyazaki, Japan) at 70 eV. Direct Analysis in Real Time (DART) ionization system was used to obtain the mass spectra of the other two ligands on an AccuTOF JMS-T100LC (JEOL, Ltd., Tokyo, Japan). MALDI-TOF MS determinations for the complexes were recorded on a Bruker Microflex (MALDI-TOF) mass spectrometer (Bruker Corporation, Billerica, MA, USA).

### 3.2. Synthesis of Compounds

#### 3.2.1. Synthesis of Benzimidazoles

Initially, benzimidazoles used as pre-ligands were obtained by condensation reactions of *o*-phenylenediamine (from Sigma-Aldrich) with the corresponding carboxylic acid, similar to previously reported procedure [66].

2-(*m*-aminophenyl)benzimidazole (**Bz1**): 3-aminobenzoic acid (1.37 g, 10 mmol) and *o*-phenylenediamine (1.08 g, 10 mmol) were mixed and stirred in polyphosphoric acid for 3 h at 160 °C. The dark violet precipitate was then washed with distilled water, purified with activated carbon and recrystallized in ethanol to give the final product **1** as a beige powder. Yield: 1.79 g, 86%. C_13_H_11_N_3_ (209.25 g·mol^−1^): Calc. C, 74.62; H, 5.30; N, 20.08. Found: C, 74.53; H, 5.33; N, 20.14%. Melting Point: 256–258 °C. IR (ATR cm^−1^): υ(N-H_2_) 3437 and 3338; δ(N-H_2_) 1619; υ(N=C)_imidazole_ 1566; υ(C=C) 1507. ^1^H NMR (DMSO-*d_6_*) δ (ppm) 12.70 (s, 1H), 7.62 (d, *J* = 7.0 Hz, 1H), 7.49 (d, *J* = 7.0 Hz, 1H), 7.43 (s, 1H), 7.28 (d, *J* = 7.5 Hz, 1H), 7.23–7.11 (m, 3H), 6.68 (d, *J* = 7.8 Hz, 1H), 5.30 (s, 2H). MS (EI, m/z): 209.

2-(*p*-aminophenyl)benzimidazole (**Bz2**): It was obtained as a pink powder according to general procedure described for Bz1 starting from 4-aminobenzoic acid (1.37 g, 10 mmol) and *o*-phenylenediamine (1.08 g, 10 mmol). Yield: 1.55 g, 74%. C_13_H_11_N_3_ (209.25 g·mol^−1^): Calc. C, 74.62; H, 5.30; N, 20.08. Found: C, 74.58; H, 5.35; N, 20.07%. Melting Point: 242–244 °C. IR (ATR cm^−1^): υ(N-H_2_) 3401 and 3328; δ(N-H_2_) 1638; υ(N=C)_imidazole_ 1561; υ(C = C) 1492. ^1^H NMR (DMSO-*d_6_*) δ (ppm) 12.42 (s, 1H), 7.84 (d, *J* = 8.4 Hz, 2H), 7.47 (s, 2H), 7.11 (dd, *J* = 6.0, 3.2 Hz, 2H), 6.73–6.49 (m, 2H), 5.59 (s, 2H). MS (EI, m/z): 209.

#### 3.2.2. Synthesis of Schiff Bases

With benzimidazoles prepared, we proceeded to carry out the synthesis of Schiff bases from salicylaldehyde and 2,4-dihydroxybenzaldehyde (from Sigma-Aldrich) by nucleophilic addition to obtain the corresponding imines in methanol or ethanol as suggested by the literature [32,36,67]. 

2-(((3-(1*H*-benzo[*d*]imidazol-2-yl)phenyl)imino)methyl)phenol (**L1**): 2-(*m*-aminophenyl)benzimidazole (1.05 g, 5 mmol) and salicylaldehyde (0.61 g, 5 mmol) were mixed and stirred in methanol at reflux. After 2 h of reaction, the yellow precipitates were washed with a cold mixture of water:methanol (1: 1) and dried in a vacuum oven for 4 h. Yield: 1.24 g, 79%. C_20_H_15_N_3_O (313.36 g·mol^−1^): Calc. C, 76.66; H, 4.83; N, 13.41. Found: C, 76.38; H, 4.77; N, 13.49%. Melting Point: 233–235 °C. IR (ATR cm^−1^): υ(N=CH)_imine_ 1602; υ(N=C)_imidazole_ 1575; υ(C=C) 1509; υ(C-O)_phenolic_ 1258. ^1^H NMR (DMSO-*d_6_*) δ (ppm) 12.99 (s, 1H, NH), 12.99 (s, 1H, OH), 9.10 (s, 1H, CH=N), 8.20 (s, 1H, Ar), 8.12 (d, 1H, Ar), 7.73 (dd, 1H, Ar), 7.65 (t, 1H, Ar), 7.64 (d, 1H, Ar), 7.56 (dd, 1H, Ar), 7.46 (t, 1H, Ar), 7.23 (dt, 2H, Ar), 7.04 (t, 1H, Ar), 7.03 (d, 1H, Ar). ^13^C{^1^H} NMR (DMSO-*d_6_*) δ (ppm) 164.52 (azomethine CH=N), 160.82 (OH-C2’’), 151.18 (imidazole CH=N), 149.28–112.38 (Ar, C). MS (EI, m/z): 313.

4-(((3-(1*H*-benzo[*d*]imidazol-2-yl)phenyl)imino)methyl)benzene-1,3-diol (**L2**): It was carried out following a procedure similar to L1, using 2-(*m*-aminophenyl)benzimidazole (1.05 g, 5 mmol) and 2,4-dihydroxybenzaldehyde (0.69 g, 5 mmol). Yield: 1.42 g, 86%. C_20_H_15_N_3_O_2_ (329.36 g·mol^−1^): Calc. C, 72.94; H, 4.59; N, 12.76. Found: C, 72.73; H, 4.63; N, 13.00%. Melting Point: 281–283 °C. IR (ATR cm^−1^): υ(N=CH)_imine_ 1605; υ(N=C)_imidazole_ 1568; υ(C=C) 1495; υ(C-O)_phenolic_ 1260. ^1^H NMR (DMSO-*d_6_*) δ (ppm) 13.47 (s, 1H, NH), 12.98 (s, 1H, OH), 10.34 (s, 1H, OH), 8.94 (s, 1H, CH=N), 8.14 (s, 1H, Ar), 8.08 (d, 1H, Ar), 7.69 (d, 1H, Ar), 7.61 (t, 1H, Ar), 7.56 (d, 1H, Ar), 7.49 (d, 1H, Ar), 7.23 (m, 2H), 6.45 (d, 1H, Ar), 6.35 (s, 1H, Ar). ^13^C{^1^H} NMR (DMSO-*d_6_*) δ (ppm) 163.69 (azomethine CH=N), 163.55 (OH-C2’’), 163.21 (OH-C4’’), 151.28 (imidazole CH=N), 149.33–102.91 (Ar, C). MS (DART+) m/z: 330.

2-(((4-(1*H*-benzo[*d*]imidazol-2-yl)phenyl)imino)methyl)phenol (**L3**): It was carried out following a procedure similar to L1, using 2-(*p*-aminophenyl)benzimidazole (1.05 g, 5 mmol) and salicylaldehyde (0.61 g, 5 mmol) in ethanol. Yield: 1.28 g, 82%. C_20_H_15_N_3_O (313.36 g·mol^−1^): Calc. C, 76.66; H, 4.83; N, 13.41. Found: C, 76.26; H, 4.73; N, 13.43%. Melting Point: 245–247 °C. IR (ATR cm^−1^): υ(N=CH)_imine_ 1600; υ(N=C)_imidazole_ 1570; υ(C=C) 1495; υ(C-O)_phenolic_ 1277. ^1^H NMR (DMSO-*d_6_*) δ (ppm) 13.00 (s, 1H, NH), 13.00 (s, 1H, OH), 9.09 (s, 1H, CH=N), 8.29 (d, 2H, Ar), 7.71 (d, 1H, Ar), 7.63 (d, 2H, Ar), 7.46 (t, 1H, Ar), 7.24 (dt, 2H, Ar), 7.02 (t, 1H, Ar), 7.01 (d,1H, Ar). ). ^13^C{^1^H} NMR (DMSO-*d_6_*) δ (ppm) 164.34 (azomethine CH=N), 160.85 (OH-C2’’), 151.24 (imidazole CH=N), 149.67–117.16 (Ar, C). MS (EI, m/z): 314.

4-(((4-(1*H*-benzo[*d*]imidazol-2-yl)phenyl)imino)methyl)benzene-1,3-diol (**L4**): It was carried out following a procedure similar to L1, using 2-(*p*-aminophenyl)benzimidazole (1.05 g, 5 mmol) and 2,4-dihydroxybenzaldehyde (0.69 g, 5 mmol) in ethanol. Yield: 1.19 g, 72%. C_20_H_15_N_3_O_2_ (329.36 g·mol^−1^): Calc. C, 72.94; H, 4.59; N, 12.76. Found: C, 73.12; H, 4.54; N, 12.91%. Melting Point: 262–263 °C. IR (ATR cm^−1^): υ(N=CH)_imine_ 1600; υ(N=C)_imidazole_ 1566; υ(C=C) 1505; υ(C-O)_phenolic_ 1277. ^1^H NMR (DMSO-*d_6_*) δ (ppm) 13.45 (s, 1H, NH), 12.91 (s, 1H, OH), 10.33 (s, 1H, OH), 8.92 (s, 1H, CH=N), 8.24 (d, 2H, Ar), 7.66 (d, 1H, Ar), 7.54 (d, 2H, Ar), 7.53 (d, 1H, Ar), 7.21 (t, 2H, Ar), 6.43 (d, 1H, Ar), 6.32 (s, 1H, Ar). ). ^13^C{^1^H} NMR (DMSO-*d_6_*) δ (ppm) 163.64 (azomethine CH=N), 163.49 (OH-C2’’), 163.28 (OH-C4’’), 151.35 (imidazole CH=N), 149.77–102.88 (Ar, C). MS (DART+) m/z: 330.

#### 3.2.3. Synthesis of Lanthanide Complexes

The general procedure for the coordination of the Schiff bases to the lanthanide ions consisted of the dropwise addition of a solution of the metal chlorides (Alfa Aesar) to a suspension of each of the ligands in methanol at room temperature. After 2 h of constant stirring, the product was filtered, and the solution was evaporated under pressure under high vacuum. The complexes obtained as powders were recrystallized from a mixture of methanol:ethanol (1:1) and dried under vacuum for 4 h. 

[La(L1)_2_(Cl)(H_2_O)_2_]·H_2_O (La-L1): A solution of LaCl_3_⋅7H_2_O (83 mg, 0.22 mmol) in MeOH (1.5 mL) was added dropwise into 20 mL of methanol solution of *L1* (2 eq: 138 mg, 0.44 mmol). The final precipitate was observed as a wheat colored powder, yield: 149 mg, 79%. C_40_H_34_ClLaN_6_O_5_ (853.1 g·mol^−1^): Calc. C, 56.32; H, 4.02; N, 9.85; La, 16.28. Found: C, 55.99; H, 4.09; N, 10.10; La, 16.00%. IR (ATR cm^−1^): υ(N = CH)_imine_ 1622; υ(N = C)_imidazole_ 1563; υ(C = C) 1506; υ(C-O)_phenolic_ 1347. ^1^H NMR (DMSO-*d_6_*) δ (ppm) 13.02 (s, 1H, NH), 9.18 (s, 1H, CH = N), 8.35 (s, 1H, Ar), 8.20 (d, 1H, Ar), 7.76 (d, 1H, Ar), 7.64 (t, 1H, Ar), 7.63 (d, 1H, Ar), 7.56 (d, 1H, Ar), 7.46 (t, 1H, Ar), 7.23 (m, 2H, Ar), 7.03 (d, 2H, Ar). ^13^C{^1^H} NMR (DMSO-*d_6_*) δ (ppm) 164.46 (azomethine CH = N), 160.81 (OH-C2’’), 151.23 (imidazole CH = N), 149.21–112.42 (Ar, C). TGA mass loss 2.10% (60-165 °C, 1 step, calc. 1 × H_2_O = 2.11%) hydration, 4.27% (165–230 °C, 1 step, calc. 2 X H_2_O = 4.31%) coordinated, 26.81% (230–500 °C, 2 steps, decomposition). MS (MALDI-TOF) m/z: 853.2. Λ (Ethanol, 26 °C) (Ω^−1^∙cm^2^∙mol^−1^): 0.012.

[Ce(L1)_2_(Cl)(H_2_O)_3_]·2H_2_O (Ce-L1): A solution of CeCl_3_⋅7H_2_O (82 mg, 0.22 mmol) in MeOH (1.5 mL) was added dropwise into 20 mL of methanol solution of *L1* (2 eq: 138 mg, 0.44 mmol). The final precipitate was observed as a golden-colored powder, yield: 145 mg, 74%. C_40_H_38_CeClN_6_O_7_ (890.3 g·mol^−1^): Calc. C, 53.96; H, 4.30; N, 9.44; Ce, 15.74. Found: C, 54.12; H, 4.34; N, 9.87; Ce, 16.11%. IR (ATR cm^−1^): υ(N=CH)_imine_ 1624; υ(N=C)_imidazole_ 1565; υ(C = C) 1507; υ(C-O)_phenolic_ 1349. TGA mass loss 3.97% (60–165 °C, 1 step, calc. 2 × H_2_O = 4.04%) hydration, 6.28% (165–230 °C, 1 step, calc. 3 X H_2_O = 6.32%) coordinated, 30.56% (230–500 °C, 1 step, decomposition). MS (MALDI-TOF) m/z: 890.7. Λ (Ethanol, 26 °C) (Ω^−1^∙cm^2^∙mol^−1^): 0.015.

[La(L2)_2_(Cl)(H_2_O)_2_]·2H_2_O·2CH_3_OH (La-L2): The synthesis of complex ***La-L2*** was performed using the same procedure as that for compound ***La-L1***. The final precipitate was observed as a dark olive-green-colored powder, yield: 161 mg, 76%. C_42_H_44_ClLaN_6_O_10_ (967.2 g·mol^−1^): Calc. C, 52.16; H, 4.59; N, 8.69; La, 14.36. Found: C, 53.07; H, 4.59; N, 8.92; La, 14.25%. IR (ATR cm^−1^): υ(N = CH)_imine_ 1624; υ(N = C)_imidazole_ 1559; υ(C = C) 1493; υ(C-O)_phenolic_ 1310. ^1^H NMR (DMSO-*d_6_*) δ (ppm) 13.48 (s, 1H, NH), 10.58 (s, 1H, OH), 9.01 (s, 1H, CH = N), 8.26 (s, 1H, Ar), 8.14 (d, 1H, Ar), 7.64 (t, 1H, Ar), 7.63 (d, 1H, Ar), 7.60 (d, 1H, Ar), 7.47 (d, 1H, Ar), 7.24 (m, 2H), 6.49 (d, 1H, Ar), 6.42 (s, 1H, Ar). ^13^C{^1^H} NMR (DMSO-*d_6_*) δ (ppm) 165.79 (azomethine CH = N), 163.60 (OH-C2’’), 163.41 (OH-C4’’), 151.31 (imidazole CH=N), 149.22–102.98 (Ar, C). TGA mass loss 6.77% (70–120 °C, 1 step, calc. 2 × CH_3_OH = 6.62%), 3.99% (120–170 °C, 1 step, calc. 2 × H_2_O = 3.99%) hydration, 4.43% (170–235 °C, 1 step, calc. 2 X H_2_O = 4.15%) coordinated, 38.49% (235–500 °C, 1 step, decomposition). MS (MALDI-TOF) m/z: 967.3. Λ (Ethanol, 26 °C) (Ω^−1^∙cm^2^∙mol^−1^): 0.037.

[Ce(L2)_2_(Cl)(H_2_O)_2_]·H_2_O·CH_3_OH (Ce-L2): The synthesis of complex ***Ce-L2*** was performed by the same method employed for complex ***Ce-L1***. The final precipitate was observed as a brown-colored powder, yield: 134 mg, 66%. C_41_H_38_CeClN_6_O_8_ (918.4 g·mol^−1^): Calc. C, 53.62; H, 4.17; N, 9.15; Ce, 15.26. Found: C, 53.83; H, 4.28; N, 9.37; Ce, 15.94%. IR (ATR cm^−1^): υ(N = CH)_imine_ 1619; υ(N = C)_imidazole_ 1555; υ(C = C) 1492; υ(C-O)_phenolic_ 1310. TGA mass loss 3.77% (75–120 °C, 1 step, calc. 1 × CH_3_OH = 3.49%), 2.22% (120–160 °C, 1 step, calc. 1 × H_2_O = 2.03%) hydration, 4.39% (160–230 °C, 1 step, calc. 2 X H_2_O = 4.15%) coordinated, 20.75% (230–500 °C, 1 step, decomposition). MS (MALDI-TOF) m/z: 918.4. Λ (Ethanol, 26 °C) (Ω^−1^∙cm^2^∙mol^−1^): 0.031.

[La(L3)_2_(Cl)(H_2_O)_3_]·2H_2_O (La-L3): Compound ***La-L3*** was attained by the same method employed for complex ***La-L1***. The final precipitate was observed as a pale-green-colored powder, yield: 138 mg, 71%. C_40_H_38_ClLaN_6_O_7_ (889.1 g·mol^−1^): Calc. C, 54.03; H, 4.31; N, 9.45; La, 15.62. Found: C, 53.88; H, 4.24; N, 9.67; La, 15.42%. IR (ATR cm^−1^): υ(N = CH)_imine_ 1634; υ(N = C)_imidazole_ 1562; υ(C = C) 1493; υ(C-O)_phenolic_ 1308. ^1^H NMR (DMSO-*d_6_*) δ (ppm) 12.97 (s, 1H, NH), 9.09 (s, 1H, CH=N), 8.35 (d, 2H, Ar), 7.72 (d, 1H, Ar), 7.61 (d, 2H, Ar), 7.45 (t, 1H, Ar), 7.22 (dd, 2H, Ar), 7.02 (t, 1H, Ar), 7.02 (d,1H, Ar). ^13^C{^1^H} NMR (DMSO-*d_6_*) δ (ppm) 164.19 (azomethine CH = N), 160.80 (OH-C2’’), 151.25 (imidazole CH = N), 149.70–114.02 (Ar, C). TGA mass loss 4.16% (70-165 °C, 1 step, calc. 2 × H_2_O = 4.05%) hydration, 6.49% (165–220 °C, 1 step, calc. 3 X H_2_O = 6.33%) coordinated, 33.69% (220–500 °C, 2 steps, decomposition). MS (MALDI-TOF) m/z: 889.5. Λ (Ethanol, 26 °C) (Ω^−1^∙cm^2^∙mol^−1^): 0.007.

[Ce(L3)_2_(Cl)(H_2_O)]·CH_3_OH (Ce-L3): Compound ***Ce-L3*** was attained by the same method employed for complex***Ce-L1***. The final precipitate was observed as a cream-colored powder, yield: 155 mg, 83%. C_41_H_34_CeClN_6_O_4_ (850.3 g·mol^−1^): Calc. C, 57.91; H, 4.03; N, 9.88; Ce, 16.48. Found: C, 57.40; H, 4.14; N, 10.04; Ce, 16.10%. IR (ATR cm^−1^): υ(N = CH)_imine_ 1635; υ(N = C)_imidazole_ 1562; υ(C = C) 1493; υ(C-O)_phenolic_ 1310. TGA mass loss 3.82% (60–120 °C, 1 step, calc. 1 × CH_3_OH = 3.76%), 2.21% (120–250 °C, 1 step, calc. 1 X H_2_O = 2.20%) coordinated, 27.31% (250–500 °C, 1 step, decomposition). MS (MALDI-TOF) m/z: 851.2. Λ (Ethanol, 26 °C) (Ω^−1^∙cm^2^∙mol^−1^): 0.011.

[La(L4)_2_(Cl)(H_2_O)_2_]·H_2_O (La-L4): Compound ***La-L4*** was attained by the same method employed for complex ***La-L1***. The final precipitate was observed as a yellow-green-colored powder, yield: 150 mg, 77%. C_40_H_34_ClLaN_6_O_7_ (885.1 g·mol^−1^): Calc. C, 54.28; H, 3.87; N, 9.50; La, 15.69. Found: C, 54.51; H, 3.98; N, 9.47; La, 15.95 IR (ATR cm^−1^): υ(N = CH)_imine_ 1635; υ(N = C)_imidazole_ 1560; υ(C = C) 1494; υ(C-O)_phenolic_ 1313. ^1^H NMR (DMSO-*d_6_*) δ (ppm) 13.46 (s, 1H, NH), 10.60 (s, 1H, OH), 8.93 (s, 1H, CH = N), 8.31 (d, 2H, Ar), 7.61 (m, 2H, Ar), 7.54 (d, 2H, Ar), 7.21 (dd, 2H, Ar), 6.48 (d, 1H, Ar), 6.40 (s, 1H, Ar). ^13^C{^1^H} NMR (DMSO-*d_6_*) δ (ppm) 165.83 (azomethine CH = N), 163.68 (OH-C2’’), 163.38 (OH-C4’’), 151.41 (imidazole CH = N), 149.77–102.88 (Ar, C). TGA mass loss 2.21% (60–110 °C, 1 step, calc. 1 × H_2_O = 2.15%) hydration, 4.33% (110–220 °C, 1 step, calc. 2 X H_2_O = 4.04%) coordinated, 21.61% (220–500 °C, 2 steps, decomposition). MS (MALDI-TOF) m/z: 885.3. Λ (Ethanol, 26 °C) (Ω^−1^∙cm^2^∙mol^−1^): 0.028.

[Ce(L4)_2_(Cl)(H_2_O)_2_]·H_2_O (Ce-L4): Compound ***Ce-L4*** was obtained by the same method employed for complex ***Ce-L1***. The final precipitate was observed as a mustard-colored powder, yield: 133 mg, 68%. C_40_H_34_CeClN_6_O_7_ (886.3 g·mol^−1^): Calc. C, 54.21; H, 3.87; N, 9.48; Ce, 15.81. Found: C, 54.35; H, 3.97; N, 9.67; Ce, 15.62%. IR (ATR cm^−1^): υ(N=CH)_imine_ 1636; υ(N = C)_imidazole_ 1559; υ(C = C) 1492; υ(C-O)_phenolic_ 1313. TGA mass loss 2.34% (65–150 °C, 1 step, calc. 1 × H_2_O = 2.14%) hydration, 4.31% (150–245 °C, 1 step, calc. 2 X H_2_O = 4.04%) coordinated, 22.14% (230–500 °C, 1 step, decomposition). MS (MALDI-TOF) m/z: 886.0. Λ (Ethanol, 26 °C) (Ω^−1^∙cm^2^∙mol^−1^): 0.036.

### 3.3. Biological Assays

#### 3.3.1. Cytotoxicity Studies

*Cell lines culture:* The antiproliferative activity studies were carried out on U251 (human glioblastoma), PC-3 (prostate cancer), K562 (myelogenous leukemia), HCT-15 (colorectal carcinoma), MCF-7 (breast epithelial adenocarcinoma), and SKLU-1 (lung carcinoma) cells, which were cultured at 37 °C in RPMI-1640 medium (Gibco) enriched with 1% nonessential amino acids (Gibco), 1% penicillin:streptomycin:amphotericin B solution, 10% fetal bovine serum (FBS, Gibco), and 2 mM L-glutamine (Invitrogen) in a humidified atmosphere of 5% (v/v) CO_2_. Trypan blue was used as indicator of cell viability. Human tumor cytotoxicity was determined under protocols approved by the NCI 1 [68]. 

*Antiproliferative activity:* The cells were removed from the tissue culture flasks by treatment with trypsin and diluted with fresh media. Of these cell suspensions, 100 µL/well of 7.5 × 10^4^ cell/mL (U251), 7.5 × 10^4^ cell/mL (PC-3), 5.0 × 10^4^ cell/mL (K562), 10.0 × 10^4^ cell/mL (HCT-15), 5.0 × 10^4^ cell/mL (MCF-7), 7.5 × 10^4^ cell/mL (SK-LU-1), and 10.0 × 10^4^ cell/mL (COS-7) of cells suspension were seeded in 96 well micro-titer plates (Costar) and incubated for 24 h at 37 °C (5% CO_2_) to allow for cell attachment. The compounds were prepared in stock concentration of 20 mM, solubilized in DMSO. For the concentration of 25 µM, the % of DMSO in the well was 0.025%. Subsequently, 100 µL of a 25 µM compound solution in DMSO was added to each well. The same concentration of vehicle was used as positive control; in all cases, DMSO was constant during measurements. The cancer cell lines were exposed to the tested compounds for 48 h. Cell culture medium without tumor cells and compounds was tested as a negative control. Reference drug used was cisplatin. 

*Cytotoxic activity on U-937 cell line:* Before conducting the antiparasitic activity studies, the protocol was followed to determine the cytotoxic activity of the compounds based on the viability of the human promonocytic cell line U-937 (ATCC CRL-1593.2^TM^) determined using the MTT enzymatic micro-method (3-(4,5-dimethylthiazol-2-yl)-2,5-diphenyl tetrazolium bromide) [28]. All of the compounds were tested in triplicate in two independent experiments. The probit probabilistic method was used to express results as 50% lethal concentration (LC_50_) [69].

#### 3.3.2. In Vitro Leishmanicidal Activity

The in vitro leishmanicidal activity of the compounds against intracellular amastigotes was evaluated using flow cytometry based on methodology described by Pulido et al. [70]. Infected cells cultivated in the absence of compounds served as a control. Reference drug was Amphotericin B. Each concentration of compound solution, as well as each control, was tested in two independent experiments. The results are expressed as the percentages of inhibition [69].

#### 3.3.3. In Vitro Trypanocidal Activity

In vitro trypanocidal activity of compounds was evaluated on *T. cruzi* cells (Tulahuen strain) for intracellular amastigotes that express the *Escherichia coli* beta-galactosidase gene. Compounds were added at concentrations of 20 μg/mL on U-937 cells infected by epimastigotes after incubation for 24 h. The experiments were performed in duplicate two times, and the results are expressed as the percentages of inhibition. Reference compound used was benznidazole, a compound conventionally used for the treatment of Chagas disease [70,71].

#### 3.3.4. In Vitro Anti-Plasmodial Activity

The *P. falciparum* 3D7 strain was grown following the procedure described by Trager and Jensen [72]. Cytotoxicity was previously determined on erythrocytes. The activity was determined in cultures in 96 well-suspension cultures using the quantification of parasite lactate dehydrogenase (pLDH) as previously described [69]. The experiment was performed in duplicate two times. Probit method was used to estimate the EC_50_ (effective concentration 50) [73].

#### 3.3.5. In Vitro Antibacterial Activity

The in vitro antibacterial activity of the compounds was determined by broth microdilution [74,75] for two Gram-positive (*S. aureus* ATCC^®^ 25923, *L. monocytogenes* ATCC^®^ 19115) and two Gram-negative (*E. coli* ATCC^®^ 25922, *P. aeruginosa* ATCC^®^ 27583) strains. The final concentration of the compounds was in the range 4–2000 μg/mL. Free medium with DMSO (0.01%) was used as a negative control, while bacterial medium without an inhibiting agent was used as positive control. Ciprofloxacin (CP) and silver nitrate (AgNO_3_) were used as control antibiotics. The bacteria were incubated with the compounds for 24 h at 37 °C to determine the minimum inhibitory concentration (MIC). To verify that the inhibition observed in the wells corresponded to the same MIC in all cases, assays were carried out in triplicate.

### 3.4. Interaction with Model Membranes

#### 3.4.1. Membrane Preparation

The 1,2-dimyristoyl-sn-glycero-3-phosphocholine (PC) and 1,2-dimyristoyl-sn-glycero-3-phosphorylglycerol (PG) lipids were used to mimic a synthetic bacterial membrane model (PC/PG ratio: 3:1) and a mammalian membrane model (PC). The lipids were suspended in a 2:1 (v/v) chloroform/methanol mixture as suggested in the literature [48]. The PC/PG mixture or only PC was dried with nitrogen and under vacuum to obtain lipid films that were subsequently hydrated with HEPES buffer or solutions containing the compounds in HEPES buffer. Multilamellar vesicles (MLVs) were obtained after three vortexing and incubation processes at 37 °C [76].

#### 3.4.2. Differential Scanning Calorimetry

From 2 mg of lipids, MLVs were obtained with ratio 1:50 (compound/lipids). The samples were placed in DSC Tzero hermetic pans and the scanning was carried out over a 10–40 °C temperature at a heating rate of 1 °C/min in DSC Q25 equipment (TA Instruments, USA); HEPES buffer was used as reference solution. To obtain the temperature of phase transition (Tm) and the transition enthalpy (DH), the Trios software (TA Instruments) was used in the analysis of the thermograms.

### 3.5. DNA Interaction Studies

The interaction studies of the compounds obtained with highly polymerized and lyophilized calf thymus DNA (CT-DNA from Sigma Aldrich) were carried out with electronic absorption experiments, following the methodology reported in the literature [77]. The DNA solutions had an A_260_/A_280_ ratio between 1.7 and 1.9, indicating that the DNA was free of RNA and proteins. 

#### Electronic Absorption Monitoring Assays

An UV-visible Evolution 220 spectrophotometer (Thermo Fischer Scientific, USA), equipped with Single Cell Peltier System for temperature control, was used to obtain the electronic spectra. The interaction studies were carried out with CT-DNA titrations at a constant concentration of the compounds (60 μM) at 25 °C, and compounds titrations at a constant concentration of CT-DNA (48 μM) at 25 °C. After the addition of each titrant, the electronic absorption spectra of the evaluated compound were measured in the range of 500–230 nm. DNA absorbance was eliminated by adding an equal amount of DNA to the sample and the standard solution [78,79]. 

### 3.6. Molecular Docking

The protein preparation, the molecule preparation, the grid generation, and the molecule-protein docking were performed using Autodock software [80]. For the protein preparation, the target proteins were initially pre-processed by removal of water molecules, addition of Kollman charges, and optimization of the hydrogen bond (H-bond). For the ligand preparation, the hydrogen atoms were added and Gasteiger charges. The coordinates of grid were obtained by CB-Dock online tool (http://cao.labshare.cn/cb-dock/, accessed on 7 May 2021) using the prepared ligand and protein. Discovery Studio Visualizer software (http://accelrys.com, accessed on 7 May 2021) was used to analyze ligand–receptor interactions to find the amino acids that interact with the molecules. 

#### 3.6.1. Construction of the Ligands and Complexes 3D Model

The 3D structure of the ligands and the complexes were realized with Chemdraw online version (https://chemdrawdirect.perkinelmer.cloud/js/sample/index.html, accessed on 7 May 2021). The structure was optimized using Avogadro version 1.2 (https://avogadro.cc/, accessed on 7 May 2021) with Universal Force Field (UFF) [81] in order to obtain PDB files for molecular docking.

#### 3.6.2. Obtaining the Receptors

The receptor proteins to be evaluated belong to the following organisms: *Leishmania spp., T. cruzi*, *P. falciparum,* and *S. aureus*. The proteins used as receptors for the ligands were leishmanin (1LML), cruzain (4PI3), tubulin Alpha (*P. falciparum*), and PBP2A of *S. aureus* (5M18). For *P. falciparum* tubulin Alpha, the GenBank sequence CAA34101 (453 aa) was used to build the 3D model using Swiss-model (https://swissmodel.expasy.org/, accessed on 7 May 2021); this was validated by PROSA (https://prosa.services.came.sbg.ac.at/prosa.php, accessed on 7 May 2021) and Molprobity (http://molprobity.biochem.duke.edu/, accessed on 7 May 2021).

#### 3.6.3. Controls

In order to compare the affinity results of ligands and complexes, control molecules were used: amphotericin for leishmanin, vinylsulfone for cruzain, vinblastine for alpha tubulin, and finally cefepime, ceftobiprole, and benzopenicillin for PB2A. These controls were first obtained in a pubchem SDF format (https://pubchem.ncbi.nlm.nih.gov/, accessed on 7 May 2021). Then, with Avogadro, they were converted to a 3D structure, and the structure was optimized using the GAFF force field. Then, the PDB format for docking was obtained.

#### 3.6.4. Validation of Docking between L2 and Leishmanin with Molecular Dynamics

The validation of leishmanin with L2 ligand was performed through molecular dynamics. The model system was hydrated by means of Charmm-Gui solution builder. The simulation was performed with GROMACS version 2020.2. Minimization was run for 0.1 ns using the steep descent algorithm, with a Verlet cutoff scheme and coulombtype pme. Equilibration was performed for 2.5 ns, with a Verlet cutoff-scheme and coulombtype pme, using the Nosé–Hoover algorithm at 310.15 K. Finally, the molecular dynamics was run for 10 ns, also with the Nosé–Hoover algorithm at temperature 310.15 K, with a Verlet cutoff scheme and coulombtype pme. RMSD analysis, hydrogen bonds, affinity energy, and the distance between L2 ligand and leishmanin were performed. The visualization of the leishmanin surface with the L2 ligand was performed with pymol educative version (https://pymol.org/edu/?q=educational/, accessed on 7 May 2021). 

## 4. Conclusions

In summary, a number of novel lanthanide complexes derived from Schiff base ligands, including a benzimidazole fragment, were obtained and unequivocally characterized using different analytical techniques. Preliminary in vitro assays were conducted to evaluate their potential pharmacological applications through various bioassays, including antiproliferative, antiparasitic, and antibacterial assays. The results suggested that structural modifications in the compounds play important roles in their biological activities, revealing that ligands L1 and L2 and their metal complexes showed lower MIC values compared with ligands L3 and L4 and their complexes, which may be due to substitutions on the aminophenol ring. Similarly, the compounds appeared to influence membrane fluidity by altering the hydrophobic core of the bilayer, which might be correlated with potential pharmacological applications. These findings could become a useful reference in further attempts to develop new pharmacological agents. Additional studies examining how the modifications affect the molecular architecture are currently ongoing in our laboratory to shed further light on the mechanism of action associated with these types of compounds.

## Figures and Tables

**Table 1:** In vitro antiparasitic and cytotoxic activities of Schiff bases and complexes *.

Compound	Cytotoxicity				Antiparasitic Activity								
	U-937 Cell Line		Erythrocytes		Anti-*L. braziliensis* ^a^			Anti-*T. cruzi* ^b^			Anti-*P. falciparum* ^c^		
	LC_50_	SD	LC_50_	SD	EC_50_	SD	SI	EC_50_	SD	SI	EC_50_	SD	SI
L1	7.77	0.46	>200	NA	>4	NA	<1.94	17.03	2.13	0.46	14.04	1.01z	>14.24
La-L1	53.65	8.06	>200	NA	106.09	61.07	0.51	62.24	8.25	0.86	13.80	1.06	>14.49
Ce-L1	44.64	10.68	>200	NA	>25	NA	<1.78	178.99	28.94	0.25	15.16	0.26	>13.19
L2	17.09	4.74	>200	NA	>8	NA	<2.13	14.97	0.05	1.14	15.57	0.92	>12.84
La-L2	18.17	3.94	>200	NA	12.67	2.47	1.43	29.27	4.62	0.62	12.81	0.54	>15.63
Ce-L2	45.37	10.12	>200	NA	26.17	1.46	1.73	56.39	4.18	0.80	13.73	0.63	>14.56
L3	20.40	5.62	>200	NA	>10	NA	<2.04	30.53	2.72	0.67	15.54	0.99	>12.87
La-L3	28.83	6.55	>200	NA	>14	NA	<2.05	>15	NA	<1.92	12.79	1.81	>15.63
Ce-L3	61.73	3.81	>200	NA	>30	NA	<2.06	130.58	14.63	0.47	16.05	0.56	>12.46
L4	14.27	1.34	>200	NA	>7	NA	<2.03	13.64	1.72	1.05	13.98	0.44	>14.30
La-L4	122.39	18.06	>200	NA	>50	NA	<2.44	>50	NA	<2.44	18.35	0.49	>10.89
Ce-L4	41.77	7.41	>200	NA	>20	NA	<2.09	>20	NA	<2.09	15.54	0.45	>12.87
Amphotericin B	36.5	2.8	ND	ND	0.31	0.08	456.3	ND	ND	ND	ND	ND	ND
Benznidazole	>200	NA	ND	ND	ND	ND	ND	11.89	3.30	>16.8	ND	ND	ND
Chloroquine	ND	ND	>200	NA	ND	ND	ND	ND	ND	ND	0.01	0.003	>200

* Results expressed in μg/mL. SD = Standard Deviation; SI = Selectivity Index; NA = Not Applicable; ND = Not Determined. Growth inhibition of U-937 cells or parasites determined by flow cytometry for intracellular amastigotes of *L. braziliensis*, by fluorometry for all forms of *P. falciparum* and by the colorimetric β-galactosidase method for intracellular amastigotes of *T. cruzi*); controls: ^a^ Amphotericin B, ^b^ Benznidazole, and ^c^ Chloroquine.

**Table 2:** In vitro antibacterial activities of Schiff bases and complexes *.

Compound	Gram-Positive Strains		Gram-Negative Strains	
	*S. aureus* ATCC^®^ 25923	*L. monocytogenes* ATCC^®^ 19115	*E. coli* ATCC^®^ 25922	*P. aeruginosa* ATCC^®^ 27583
L1	500	500	2000	2000
La-L1	125	500	500	1000
Ce-L1	250	500	500	1000
L2	500	500	2000	2000
La-L2	250	500	1000	1000
Ce-L2	250	500	1000	1000
L3	250	1000	500	2000
La-L3	500	1000	500	2000
Ce-L3	500	1000	1000	2000
L4	250	1000	1000	2000
La-L4	250	1000	2000	2000
Ce-L4	250	1000	2000	2000
CP ^a^	0.50	0.50	<0.008	>4.00
ÂgNO_3_^b^	<100	<100	<100	<100

* Results expressed in μg/mL; controls: ^a^ Ciprofloxacin (CP) and ^b^ Silver nitrate (AgNO_3_).

**Table 3:** *Tm* and enthalpy transition (DH) values of multilamellar vesicles (MLVs) formed by DMPC and DMPC:DMPG (3:1) before and after addition of ligands and La complexes at a 1:50 compound:lipid molar ratio.

MLV	Compound	Pretransition Temperature (°C)	Tm (°C)	DH (J·g^−1^)
DMPC	-	12.94	23.02	1.48
	L1	-	22.39	0.82
	La-L1	-	22.85	0.62
	L2	-	22.94	1.05
	La-L2	-	22.22	0.57
	L3	-	22.22	0.54
	La-L3	-	22.46	0.25
	L4	-	22.53	0.70
	La-L4	-	21.60	0.21
DMPC/DMPG (3:1)	-	12.71	23.02	1.72
	L1	-	22.41	1.48
	La-L1	-	23.55	0.61
	L2	-	23.06	1.46
	La-L2	-	22.58	0.51
	L3	-	23.39	1.50
	La-L3	-	21.64	1.31
	L4	-	22.86	1.41
	La-L4	-	22.47	1.38

**Table 4:** Intrinsic binding constants (Kb) for all compounds evaluated.

Compound	Band Followed (nm)	Estimated Constant (10^4^ M^−1^)
L1	300	0.3
L2	297	1.1
L3	314	1.3
L4	420	0.7
La-L1	299	1.6
La-L2	299	1.3
La-L3	318	1.6
La-L4	323	1.4
Ce-L1	295	0.6
Ce-L2	294	1.1
Ce-L3	329	2.2
Ce-L4	324	1.8

## Data Availability

Data is contained within the article.

## Supplementary Materials

The following are available online at https://www.mdpi.com/article/10.3390/antibiotics10060728/s1. Figure S1. FT-IR spectra of Bz1, L1, La-L1 and Ce-L1; Figure S2. FT-IR spectra of Bz1, L2, La-L2 and Ce-L2; Figure S3. FT-IR spectra of Bz2, L3, La-L3 and Ce-L3; Figure S4. FT-IR spectra of Bz2, L4, La-L4 and Ce-L4; Figure S5. NMR-1H spectra of Bz1; Figure S6. NMR-1H spectra of Bz2; Figure S7. NMR-1H and 13C{1H} spectra of L1; Figure S8. NMR-1H and 13C{1H} spectra of L2; Figure S9. NMR-1H and 13C{1H} spectra of L3; Figure S10. NMR-1H and 13C{1H} spectra of L4; Figure S11. NMR-1H and 13C{1H} spectra of La-L1; Figure S12. NMR-1H and 13C{1H} spectra of La-L2; Figure S13. NMR-1H and 13C{1H} spectra of La-L3; Figure S14. NMR-1H and 13C{1H} spectra of La-L4; Figure S15. Mass spectra (EI) of Bz1; Figure S16. Mass spectra (EI) of Bz2; Figure S17. Mass spectra (EI) of L1; Figure S18. Mass spectra (DART+) of L2 [M+1]; Figure S19. Mass spectra (EI) of L3; Figure S20. Mass spectra (DART+) of L4 [M+1]; Figure S21. Mass spectra (MALDI-TOF) of La-L1; Figure S22. Mass spectra (MALDI-TOF) of Ce-L1 Figure S23. Mass spectra (MALDI-TOF) of La-L2; Figure S24. Mass spectra (MALDI-TOF) of Ce-L2; Figure S25. Mass spectra (MALDI-TOF) of La-L3; Figure S26. Mass spectra (MALDI-TOF) of Ce-L3; Figure S27. Mass spectra (MALDI-TOF) of La-L4; Figure S28. Mass spectra (MALDI-TOF) of Ce-L4; Figure S29. Simultaneous TG-DTG curves of La-L1; Figure S30. Simultaneous TG-DTG curves of Ce-L1; Figure S31. Simultaneous TG-DTG curves of La-L2; Figure S32. Simultaneous TG-DTG curves of Ce-L2; Figure S33. Simultaneous TG-DTG curves of La-L3; Figure S34. Simultaneous TG-DTG curves of Ce-L3; Figure S35. Simultaneous TG-DTG curves of La-L4; Figure S36. Simultaneous TG-DTG curves of Ce-L4; Figure S37. Photometric study at 60 μM of (A) La-L1, (B) La-L2, (C) La-L3 and (D) La-L4, with CT-DNA at concentrations between 0 and 96 μM (solid black line = 0 μM). Figure S38. Photometric study at 60 μM of (A) Ce-L1, (B) Ce-L2, (C) Ce-L3 and (D) Ce-L4, with CT-DNA at concentrations between 0 and 96 μM (solid black line = 0 μM). Figure S39. Photometric study of CT-DNA at 48 μM with (A) L1, (B) L2, (C) L3 and (D) L4, at concentrations between 0 and 60 μM. Figure S40. Photometric study of CT-DNA at 48 μM with (A) La-L1, (B) La-L2, (C) La-L3 and (D) La-L4, at concentrations between 0 and 60 μM. Figure S41. Photometric study of CT-DNA at 48 μM with (A) Ce-L1, (B) Ce-L2, (C) Ce-L3 and (D) Ce-L4, at concentrations between 0 and 60 μM (solid black line = 0 μM). Figure S42. Wolf–Shimer plots for (A) L1, (B) L2, (C) L3 and (D) L4. Figure S43. Wolf–Shimer plots for (A) La-L1, (B) La-L2, (C) La-L3 and (D) La-L4. Figure S44. Wolf–Shimer plots for (A) Ce-L1, (B) Ce-L2, (C) Ce-L3 and (D) Ce-L4. Figure S45. Interaction between ligands and cruzain residues. (A) Ligand 1 (B) Ligand 2 (C) Ligand 3 (D) Ligand 4. Table S1. Bands present in the IR spectra of Bz1, Bz2, Schiff bases ligands and their lanthanide complexes (values in cm-1); Table S2. NMR-1H spectral data of Schiff bases ligands and lanthanide complexes*; Table S3. Interaction of the molecules with the cruzain; Table S4. Interaction of the molecules with the leishmanin; Table S5. Interaction of the molecules with alpha tubulin; Table S6. Interaction of the molecules with *S. aureus* PBP2A.
