# Supplementary material for: In Vitro Evaluation of the Potential Pharmacological Activity and Molecular Targets of New Benzimidazole-Based Schiff Base Metal Complexes
Source: Antibiotics (Basel). 2021 Jun 16;10(6):728. doi: 10.3390/antibiotics10060728 (PMC8235109; doi:10.3390/antibiotics10060728)
Supplement: Supplementary file 1 [file antibiotics-10-00728-s001.zip › antibiotics-1234251-supplementary.pdf]

Supporting Materials for:

In vitro Evaluation of the Potential Pharmacological Activity and Molecular Targets of New Benzimidazole-Based Schiff Base Metal Complexes

Alberto Aragón-Muriel [1], Yamil Liscano [2], Yulieth Upegui [3], Sara M. Robledo [3], María Teresa Ramírez-Apan [4], David Morales-Morales [4], Jose Oñate-Garzón [2] and Dorian Polo-Cerón [1,]*

[1] Laboratorio de Investigación en Catálisis y Procesos (LICAP), Departamento de Química, Facultad de Ciencias Naturales y Exactas, Universidad del Valle, 760001 Cali, Colombia; alberto.aragon@correounivalle.edu.co (A.A.-M.); dorian.polo@correounivalle.edu.co (D.P.-C.)
[2] Grupo de Investigación en Química y Biotecnología (QUIBIO), Facultad de Ciencias Básicas, Universidad Santiago de Cali, 760031 Cali, Colombia; yamil.liscano00@usc.edu.co (Y.L.); jose.onate00@usc.edu.co (J.O.-G.)
[3] PECET, Facultad de Medicina, Universidad de Antioquia, 050010 Medellín, Colombia; yulieth.upegui@udea.edu.co (Y.U.); sara.robledo@udea.edu.co (S.M.R.)
[4] Instituto de Química, Universidad Nacional Autónoma de México, Cd. Universitaria, Circuito Exterior, Coyoacán, 04510 México D.F., México; damor@unam.mx (D.M.-M.); mtrapan@yahoo.com.mx (M.R.-A.)

* Correspondence: dorian.polo@correounivalle.edu.co

1. FT-IR spectral data

Table S1. Bands present in the IR spectra of Bz1, Bz2, Schiff bases ligands and their lanthanide complexes (values in cm^{-1})

Compound	υ(N-H₂)	δ(N-H₂)	υ(N=CH)	υ(N=C)	υ(C=C)	υ(C-O)
Bz1	3437, 3338	1619	-	1566	1507	-
L1	-	-	1602	1575	1509	1258
La-L1	-	-	1622	1563	1506	1347
Ce-L1	-	-	1624	1565	1507	1349
L2	-	-	1605	1568	1495	1260
La-L2	-	-	1624	1559	1493	1310
Ce-L2	-	-	1619	1555	1492	1310
Bz2	3401, 3328	1638	-	1561	1492	-
L3	-	-	1600	1570	1495	1277
La-L3	-	-	1634	1562	1493	1308
Ce-L3	-	-	1635	1562	1493	1310
L4	-	-	1600	1566	1505	1277
La-L4	-	-	1635	1560	1494	1313
Ce-L4	-	-	1636	1559	1492	1313

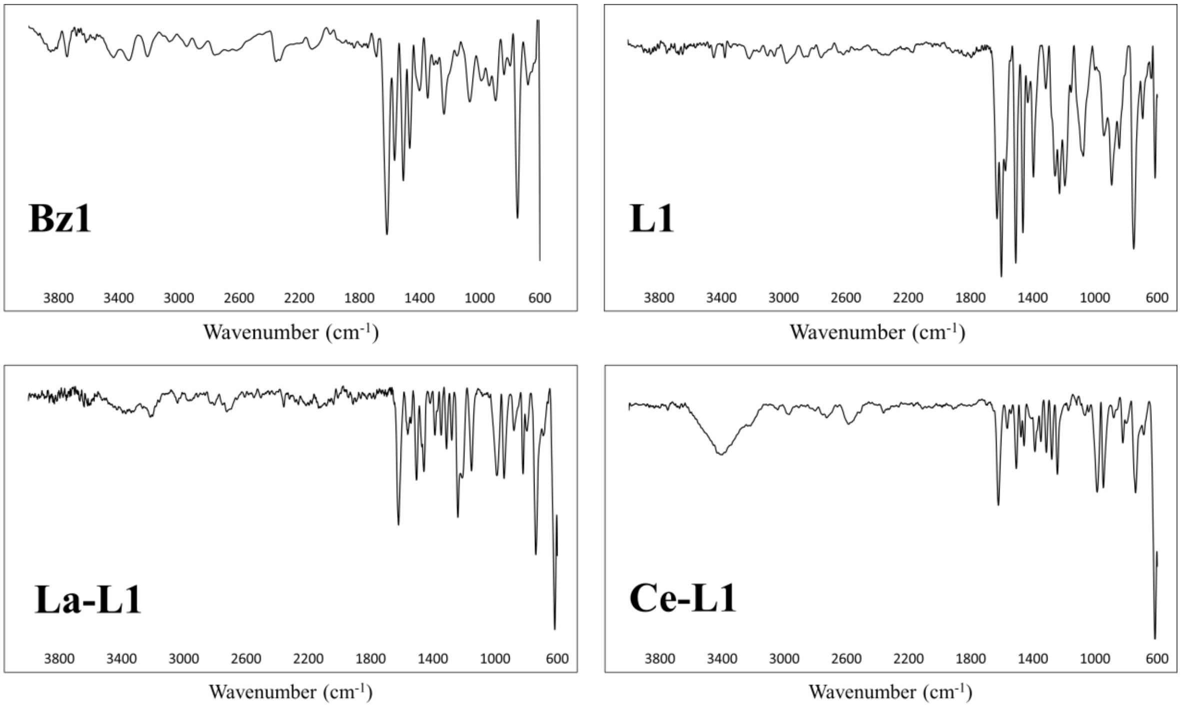

Figure S1. FT-IR spectra of *Bz1*, *L1*, *La-L1* and *Ce-L1*.

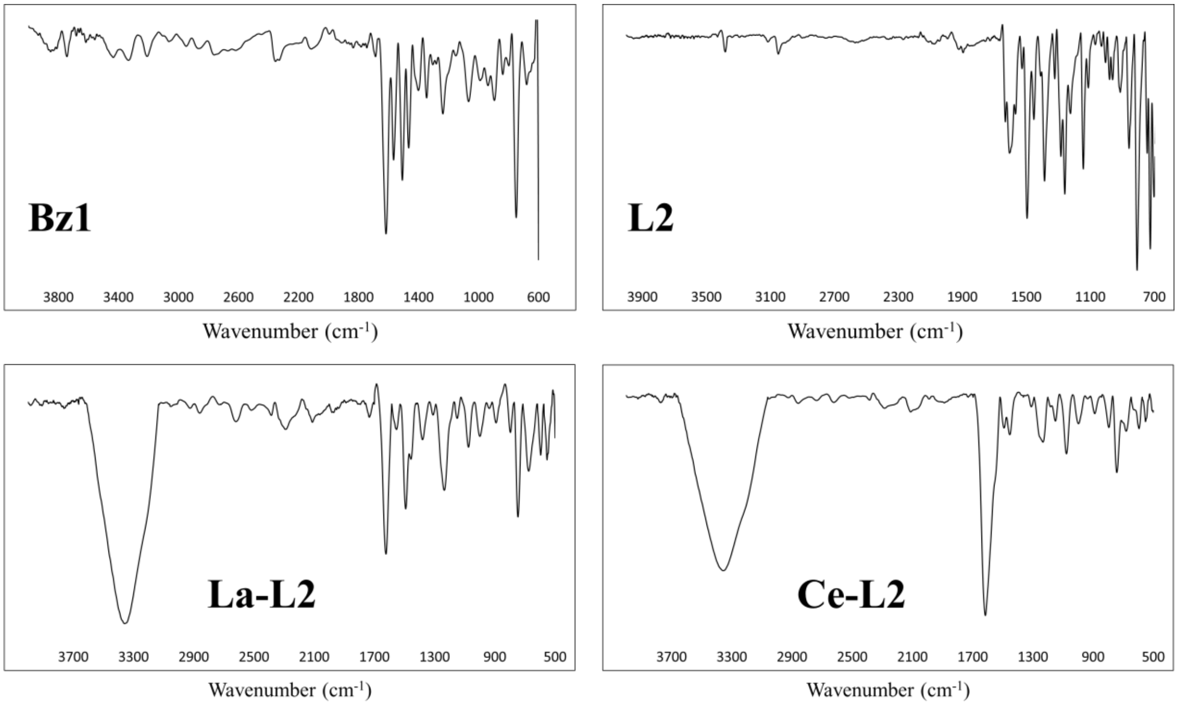

Figure S2. FT-IR spectra of *Bz1*, *L2*, *La-L2* and *Ce-L2*.

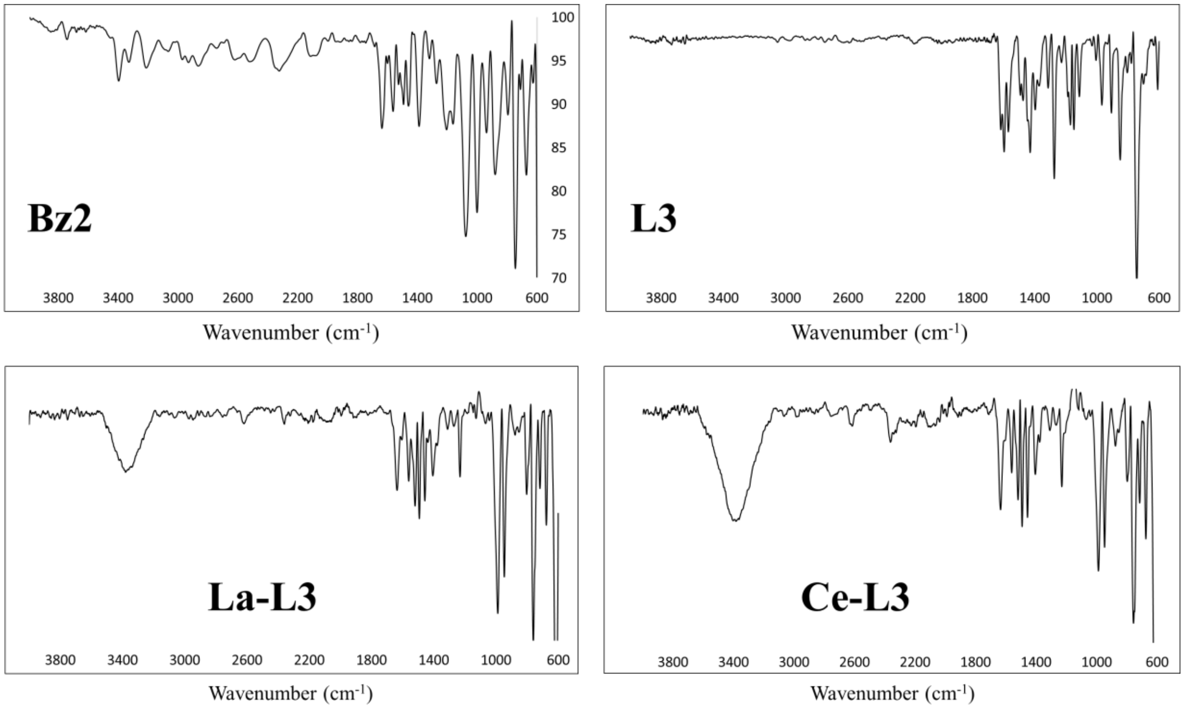

Figure S3. FT-IR spectra of *Bz2*, *L3*, *La-L3* and *Ce-L3*.

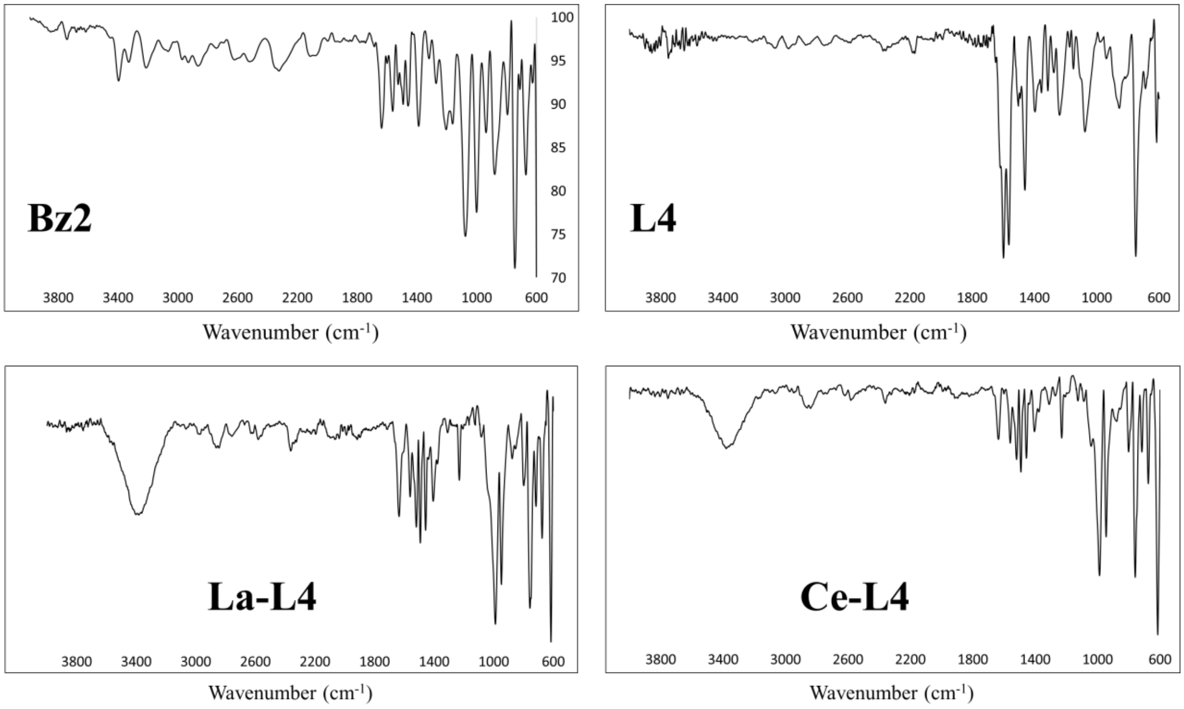

Figure S4. FT-IR spectra of *Bz2*, *L4*, *La-L4* and *Ce-L4*.

2. NMR spectral data

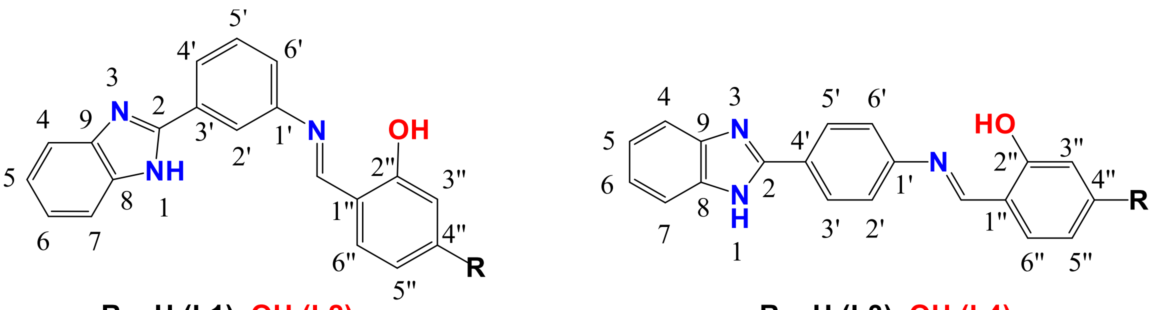

R = H (L1), OH (L2) **R = H (L3), OH (L4)**

Scheme S1. Structure of Schiff bases ligands.

Table S2. NMR-^{1}H spectral data of Schiff bases ligands and lanthanide complexes[*]

Compound	Benzimidazole ring				Aminophenyl ring					Salicylidene ring				
	NH	H_4	H_7	$H_{5,6}$	$H_{2'}$	$H_{4'}$	$H_{5'}$	$H_{6'}$	N=CH	OH	$H_{3''}$	$H_{4''}$	$H_{5''}$	$H_{6''}$
L1	12.99s	7.56dd (7.6, 2.2)	7.73dd (8.1,1.8)	7.23dt (6.4,3.5)	8.20s	8.12d (7.7)	7.65t (7.8)	7.64d (7.8)	9.10s	12.99s	7.03d (7.4)	7.46t (7.8)	7.04t (7.4)	7.64d (7.8)
La-L1	13.02s	7.56d (7.7)	7.76d (7.5)	7.23m	8.35s	8.20d (7.5)	7.64t (7.2)	7.63d (7.2)	9.18s	-	7.03d (7.8)	7.46t (7.9)	7.03d (7.8)	7.63d (7.2)
	NH	H_4	H_7	$H_{5,6}$	$H_{2'}$	$H_{4'}$	$H_{5'}$	$H_{6'}$	N=CH	OH(2)	$H_{3''}$	OH(4)	$H_{5''}$	$H_{6''}$
L2	13.47s	7.56d (7.7)	7.69d (7.8)	7.23m	8.14s	8.08d (7.7)	7.61t (7.8)	7.49d (7.9)	8.94s	12.98s	6.35s	10.34s	6.45d (8.5)	7.51d (8.6)
La-L2	13.48s	7.60d (7.8)	7.63d (7.6)	7.24m	8.26s	8.14d (7.7)	7.64t (7.6)	7.47d (8.6)	9.01s	-	6.42s	10.58s	6.49d (8.5)	7.52d (8.7)
	NH	H_4	H_7	$H_{5,6}$	$H_{2',6'}$	$H_{3',5'}$			N=CH	OH	$H_{3''}$	$H_{4''}$	$H_{5''}$	$H_{6''}$
L3	13.00s	7.63d (8.4)	7.71d (7.7)	7.24dt (6.2,3.5)	7.63d (8.4)	8.29d (8.4)			9.09s	13.00s	7.01d (7.7)	7.46t (7.8)	7.02t (7.7)	7.63d (8.4)
La-L3	12.97s	7.61d (8.3)	7.72d (7.6)	7.22dd (5.8,3.0)	7.61d (8.3)	8.35d (8.3)			9.09s	-	7.02d (6.4)	7.45t (7.7)	7.02t (6.4)	7.61d (8.3)
	NH	H_4	H_7	$H_{5,6}$	$H_{2',6'}$	$H_{3',5'}$			N=CH	OH(2)	$H_{3''}$	OH(4)	$H_{5''}$	$H_{6''}$
L4	13.45s	7.53d (7.4)	7.66d (7.2)	7.21t (9.2)	7.54d (8.6)	8.24d (8.6)			8.92s	12.91s	6.32s	10.33s	6.43d (8.5)	7.47d (8.5)
La-L4	13.46s	7.61m	7.61m	7.21dd (5.9,3.0)	7.54d (8.3)	8.31d (8.4)			8.93s	-	6.40s	10.60s	6.48d (8.5)	7.49d (8.5)

[*]Spectra have been recorded in DMSO-*d*6; δ in ppm and coupling constant in Hz are given in parentheses.

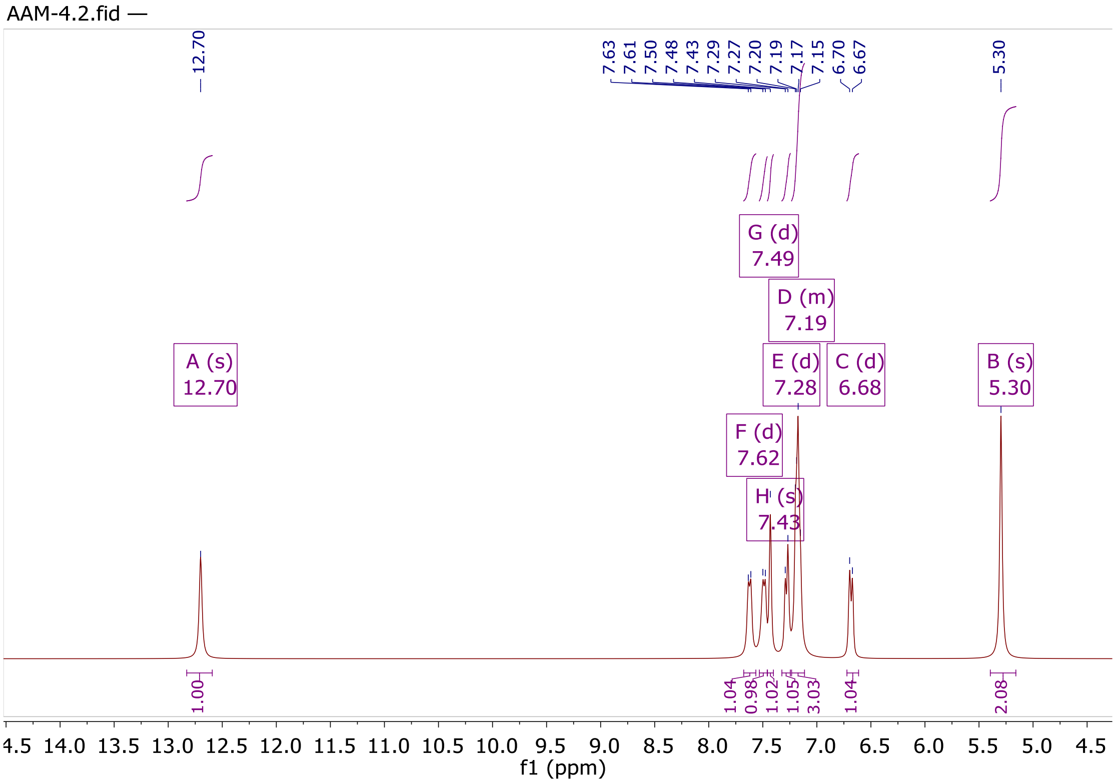

Figure S5. NMR-^{1}H spectra of *Bz1*

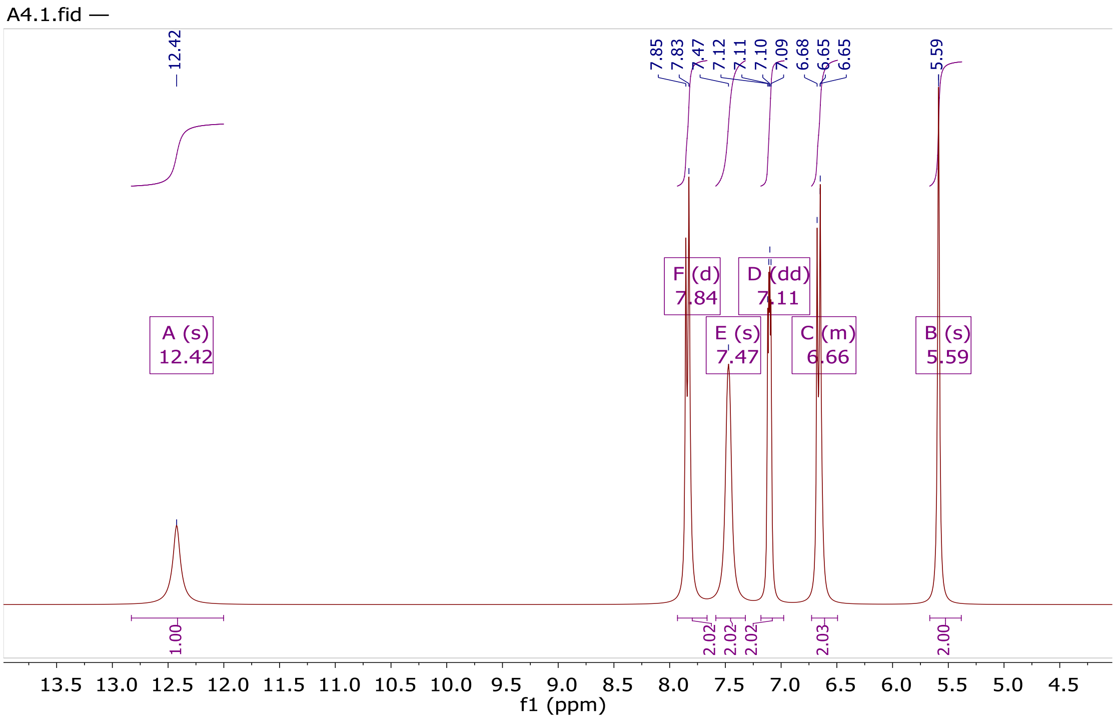

Figure S6. NMR-^{1}H spectra of *Bz2*

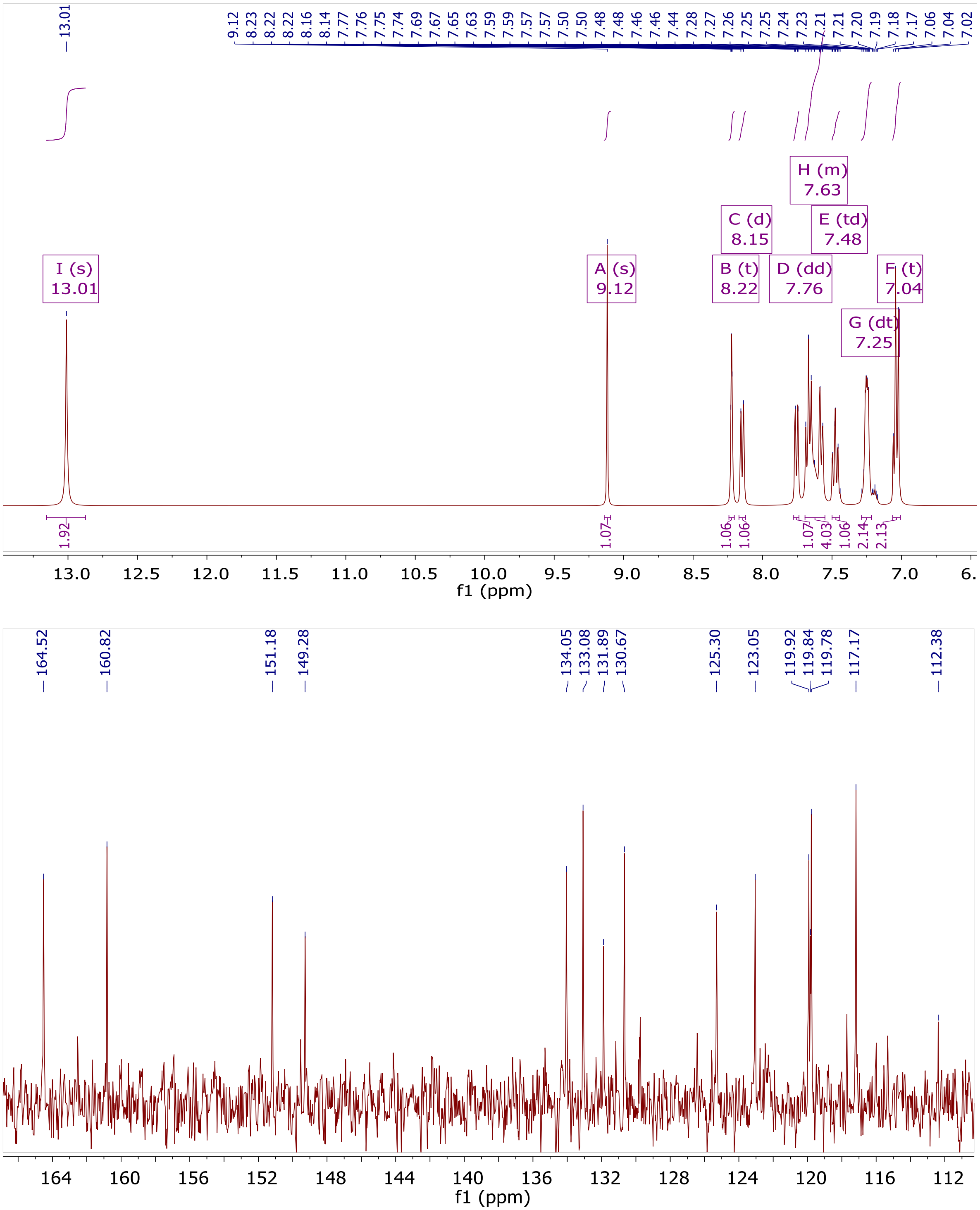

Figure S7. NMR-^{1}H and $^{13}C\{^{1}H\}$ spectra of *L1*

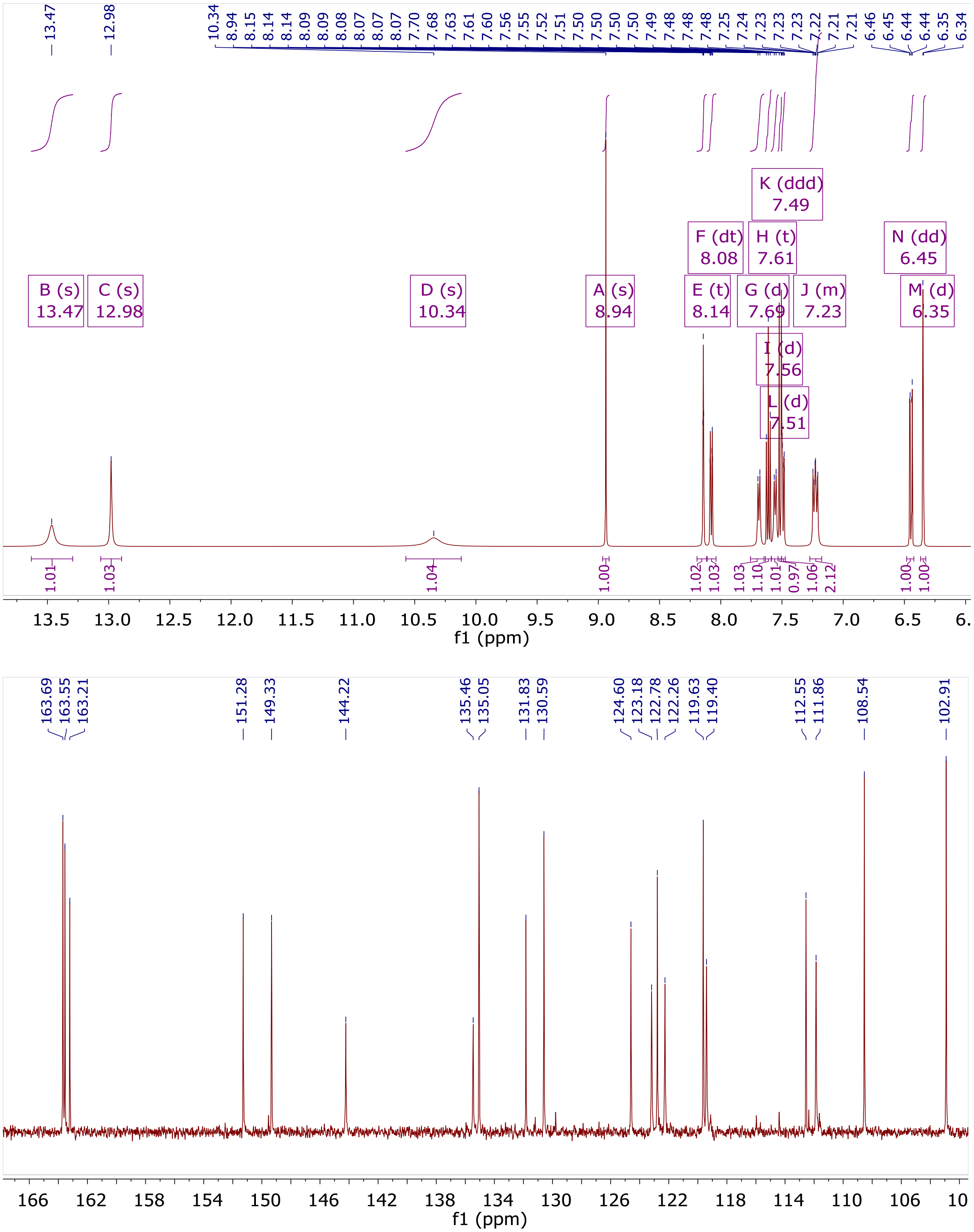

Figure S8. NMR-^{1}H and $^{13}C\{^{1}H\}$ spectra of *L2*

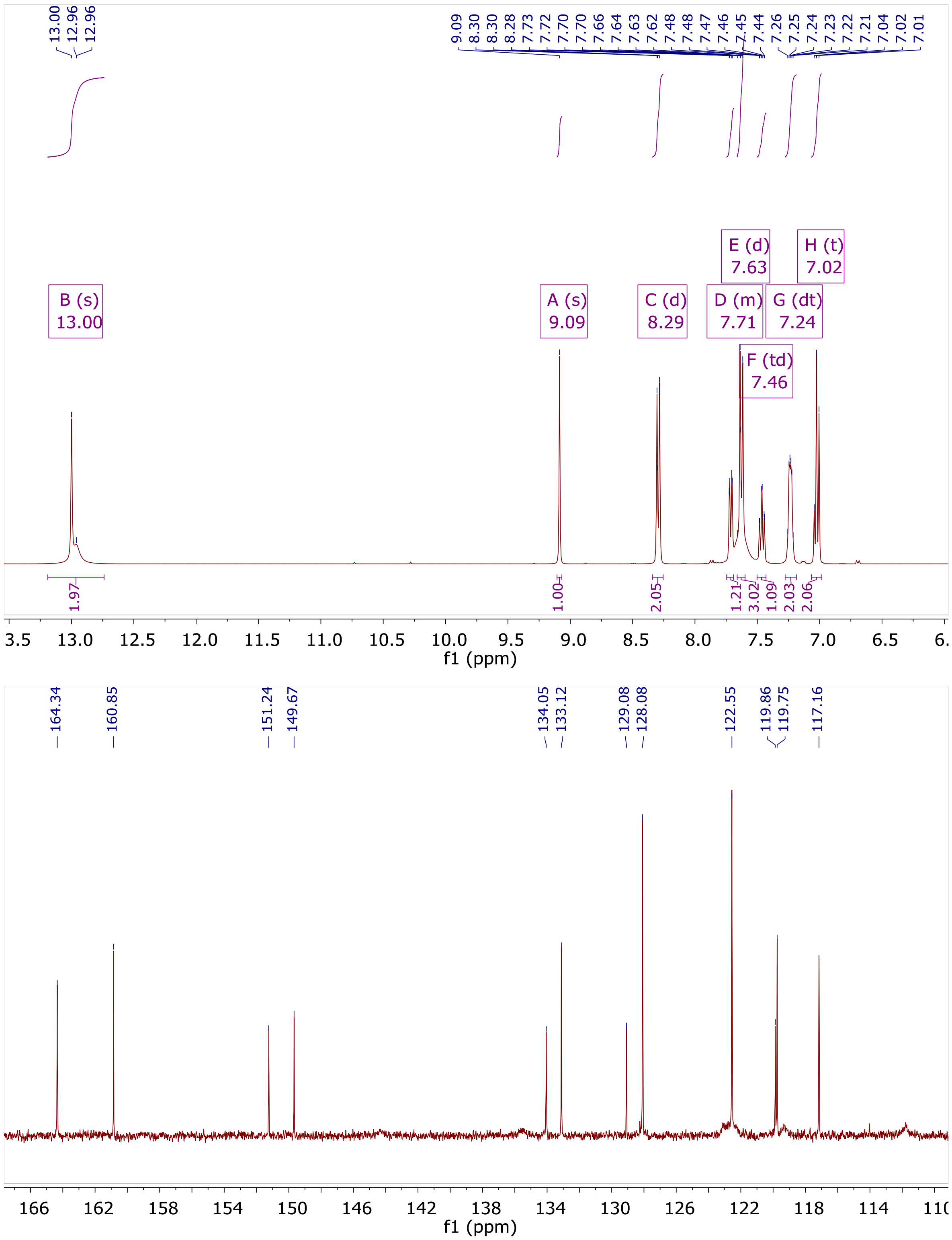

Figure S9. NMR-^{1}H and ^{13}C{^{1}H} spectra of *L3*

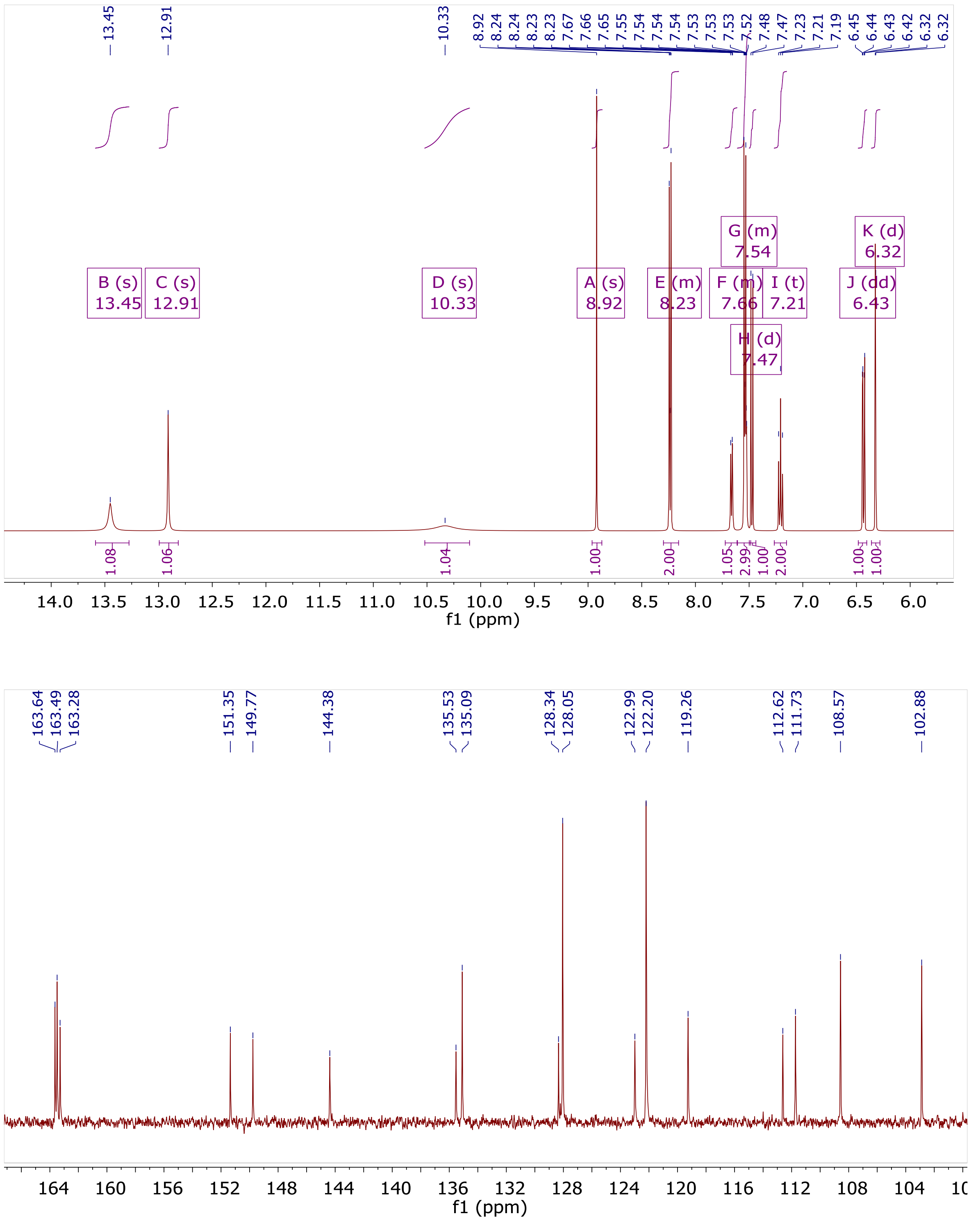

Figure S10. NMR-^{1}H and $^{13}C\{^{1}H\}$ spectra of *L4*

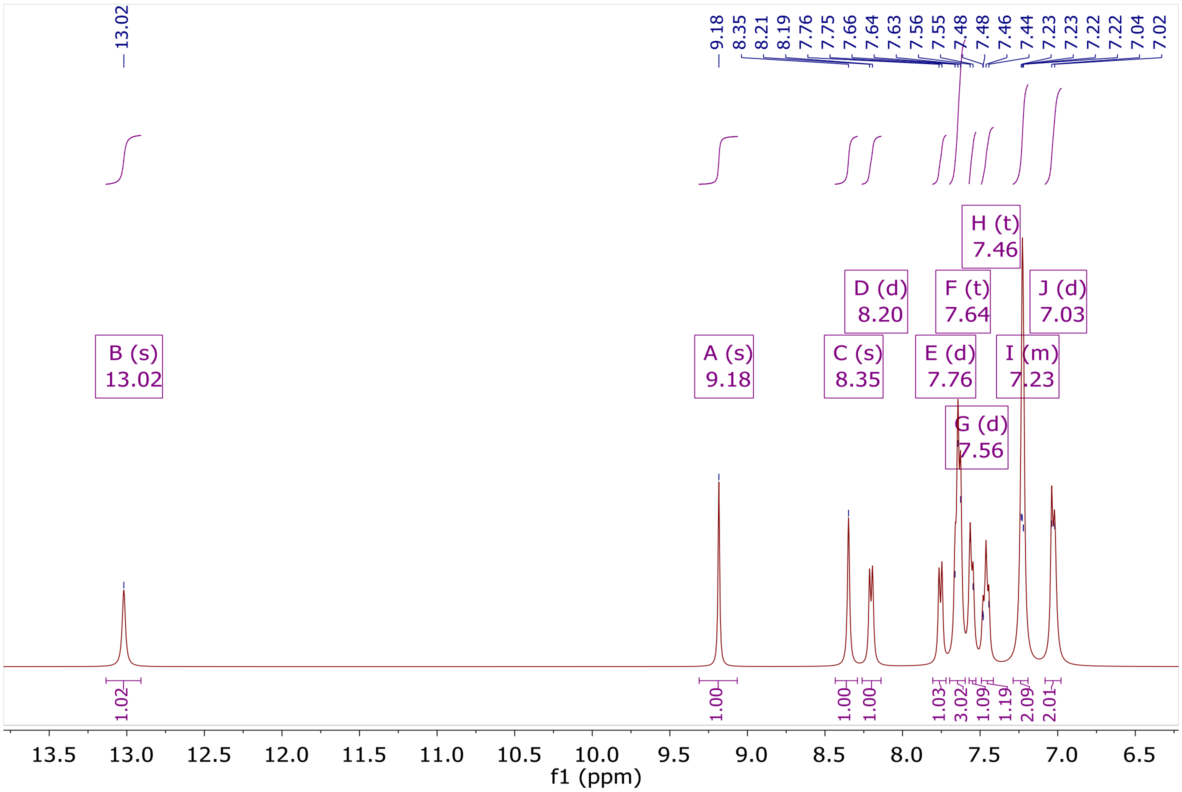

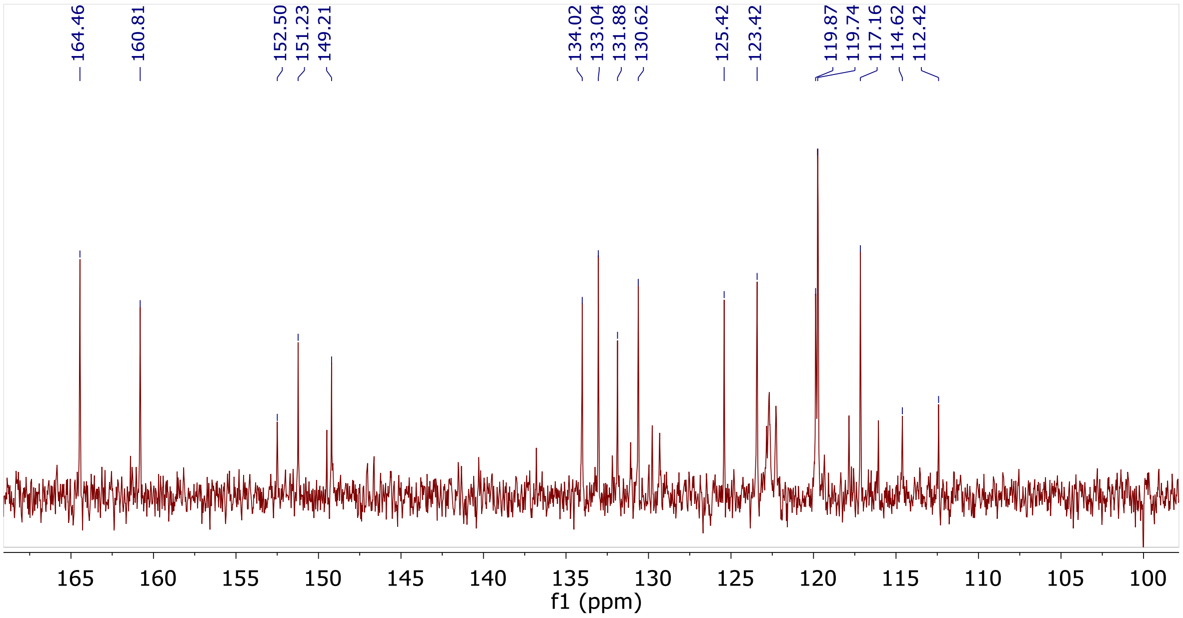

Figure S11. NMR-^{1}H and ^{13}C{^{1}H} spectra of *La-L1*

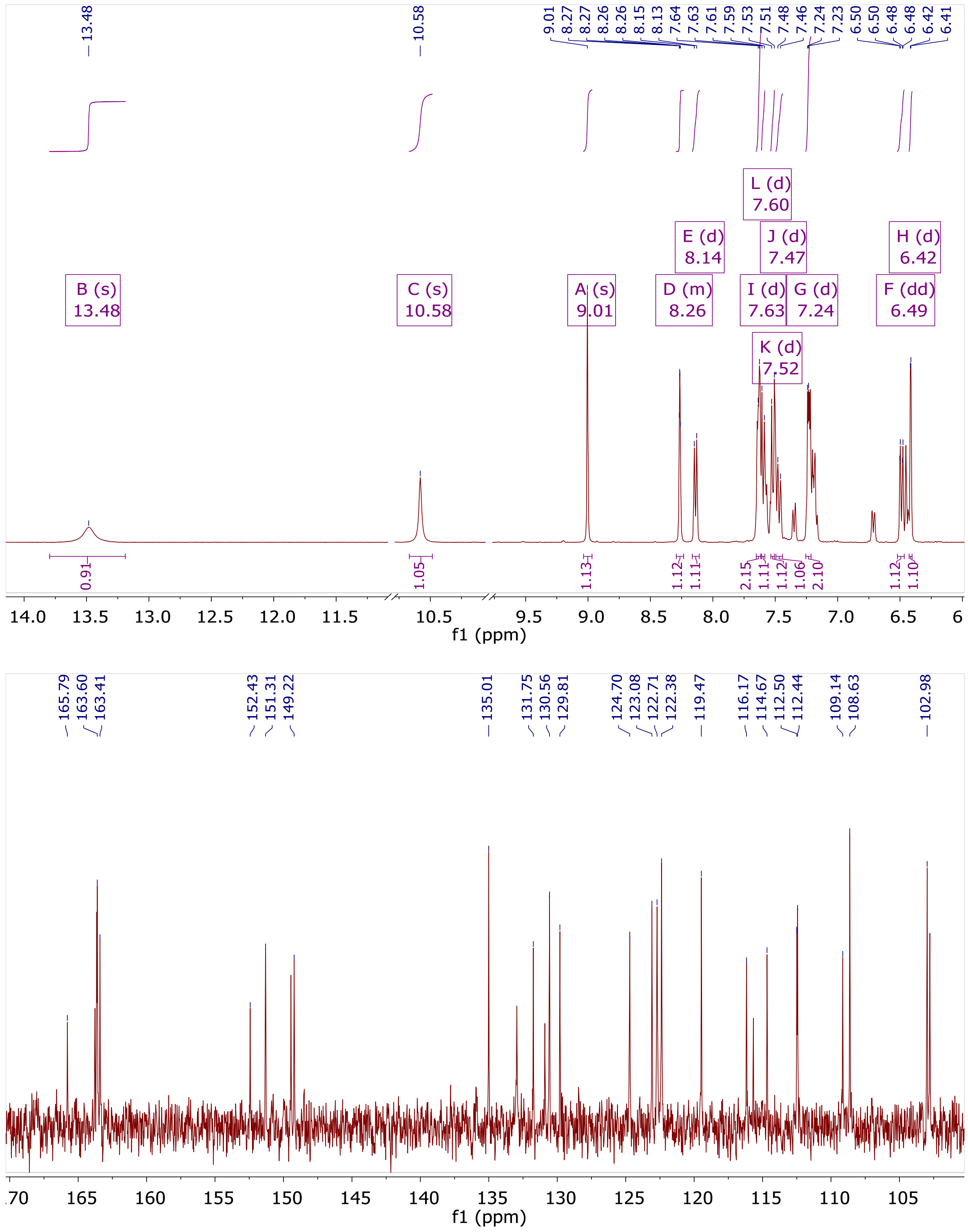

Figure S12. NMR-^{1}H and ^{13}C{^{1}H} spectra of *La-L2*

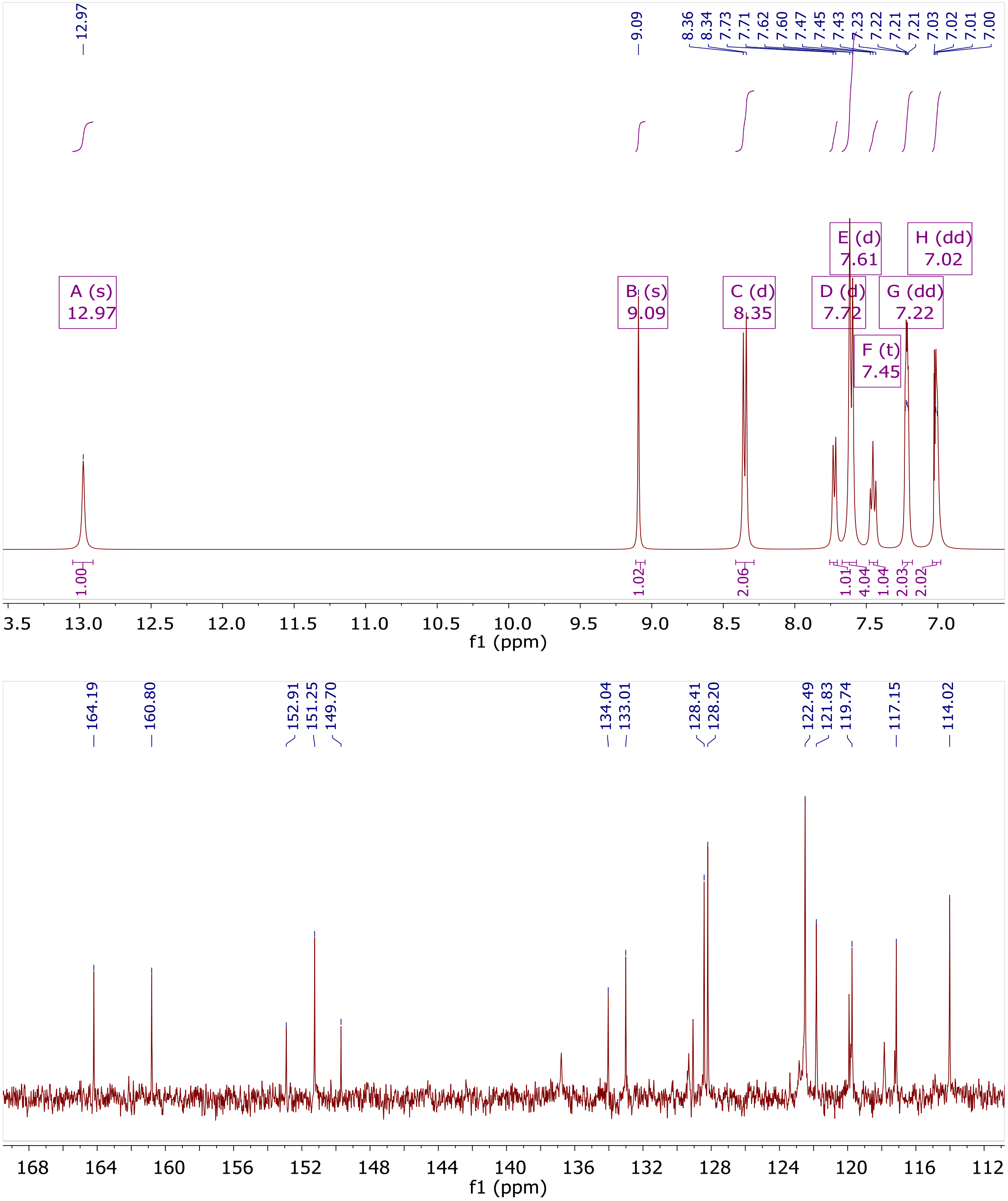

Figure S13. NMR-^{1}H and ^{13}C{^{1}H} spectra of *La-L3*

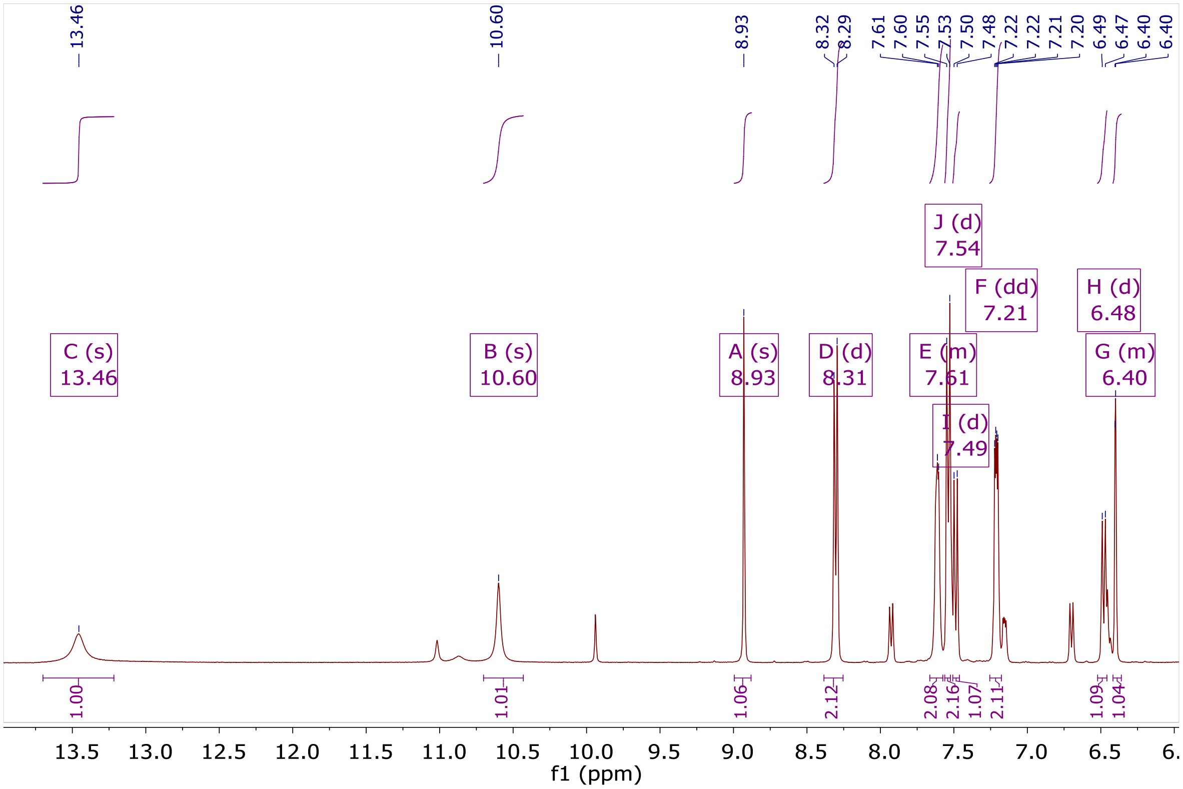

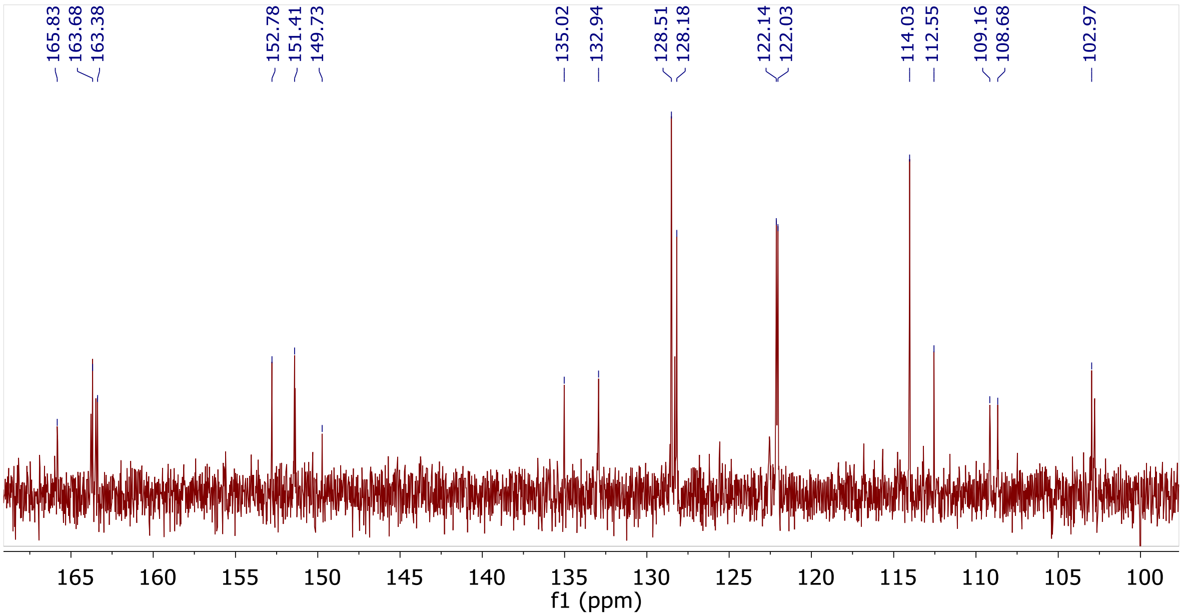

Figure S14. NMR-^{1}H and $^{13}C\{^{1}H\}$ spectra of *La-L4*

3. Mass spectral data

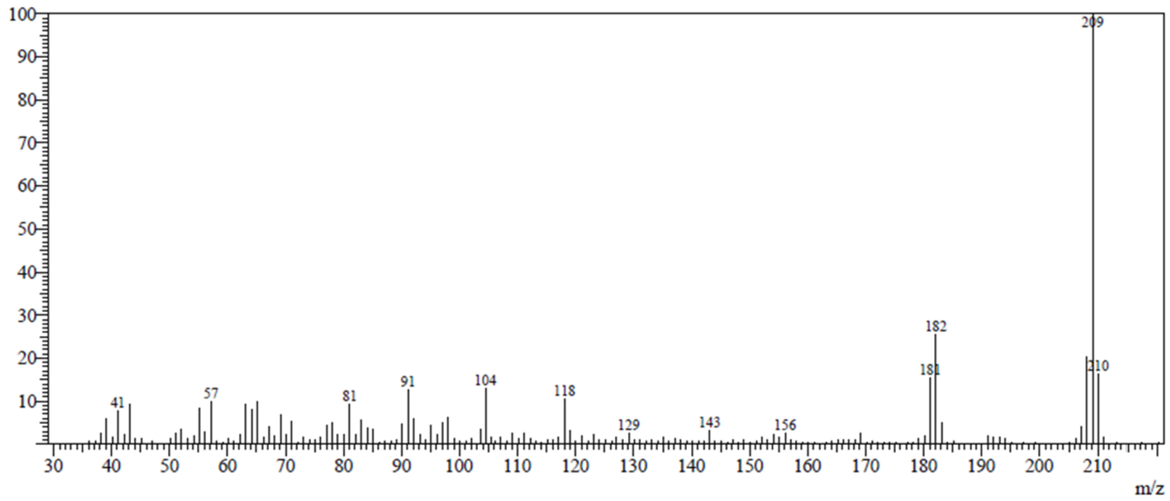

Figure S15. Mass spectra (EI) of *Bz1*

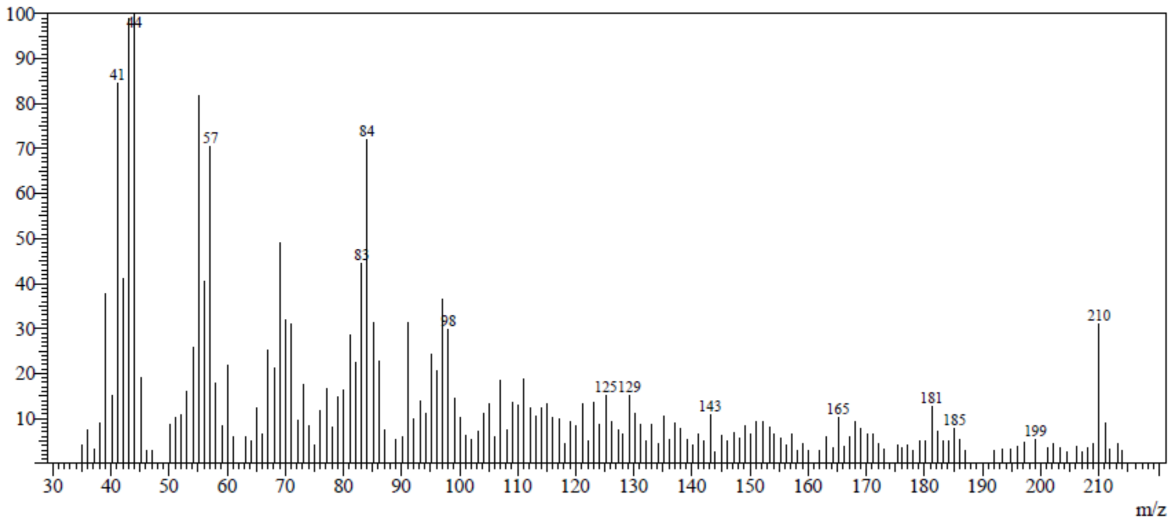

Figure S16. Mass spectra (EI) of *Bz2*

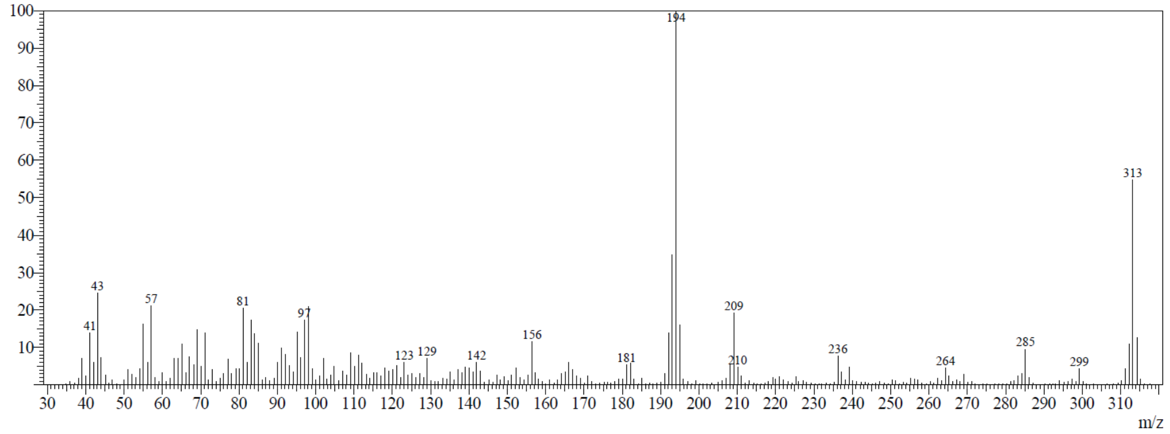

Figure S17. Mass spectra (EI) of *L1*

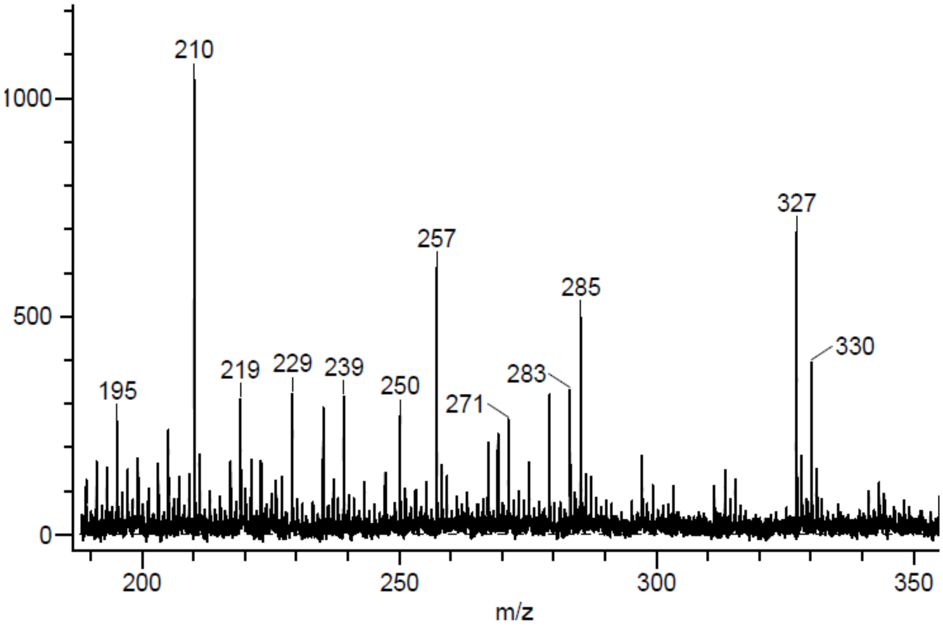

Figure S18. Mass spectra (DART+) of *L2* [M+1]

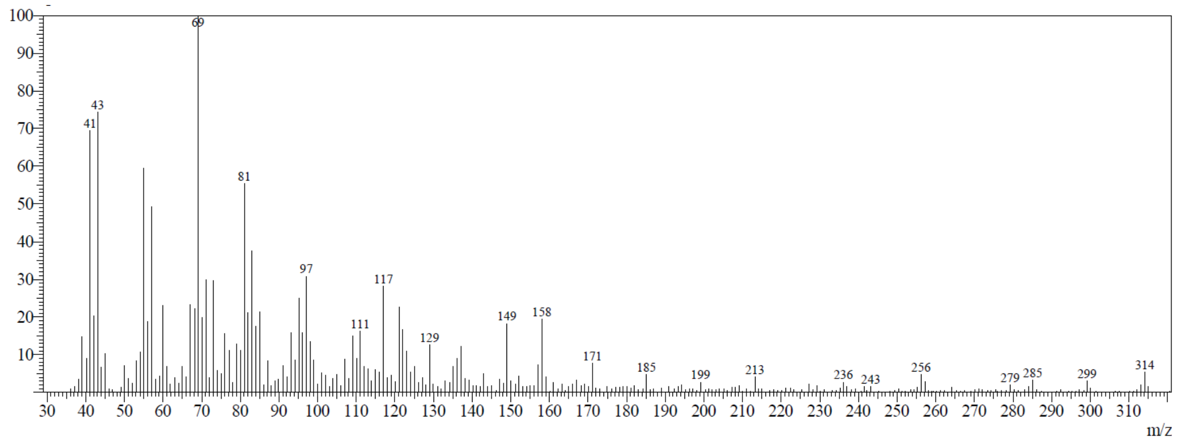

Figure S19. Mass spectra (EI) of *L3*

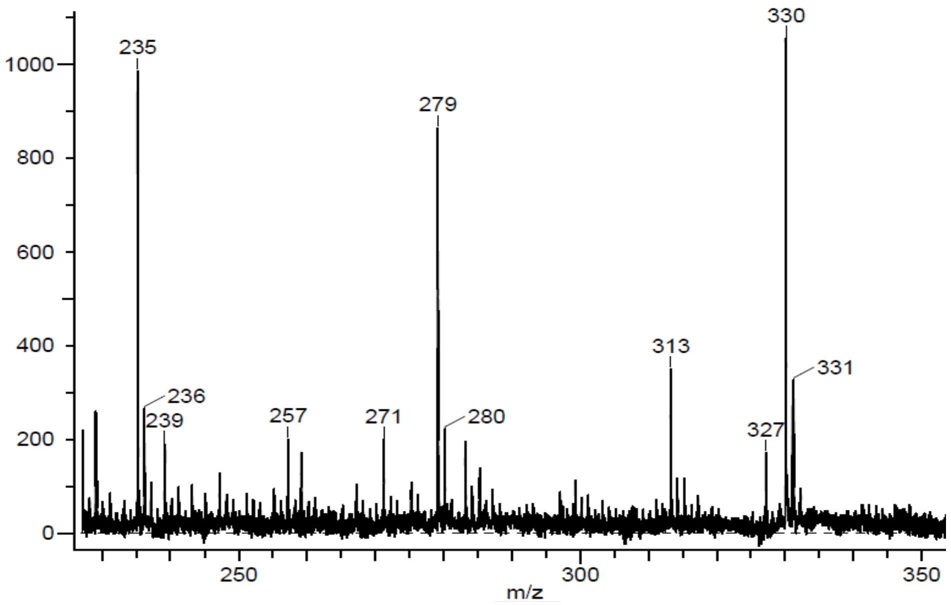

Figure S20. Mass spectra (DART+) of *L4* [M+1]

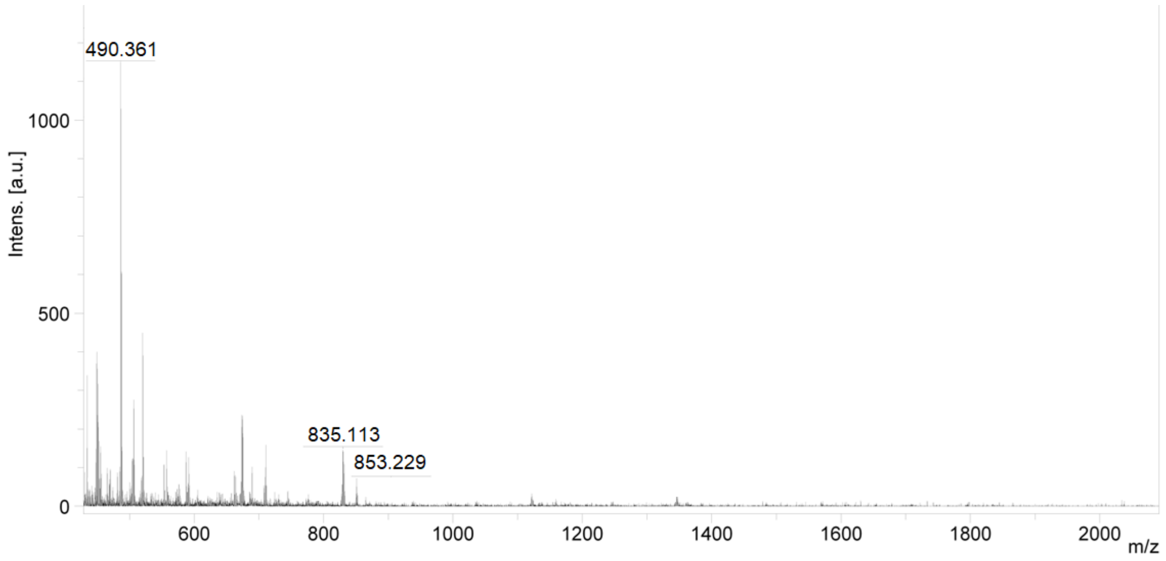

Figure S21. Mass spectra (MALDI-TOF) of *La-L1*

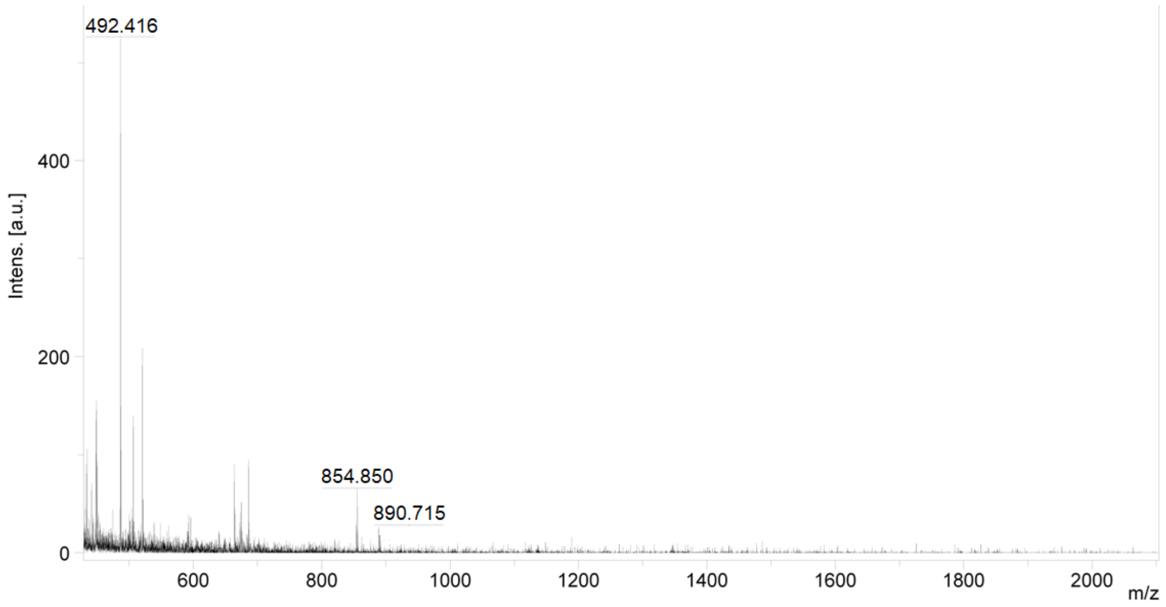

Figure S22. Mass spectra (MALDI-TOF) of *Ce-L1*

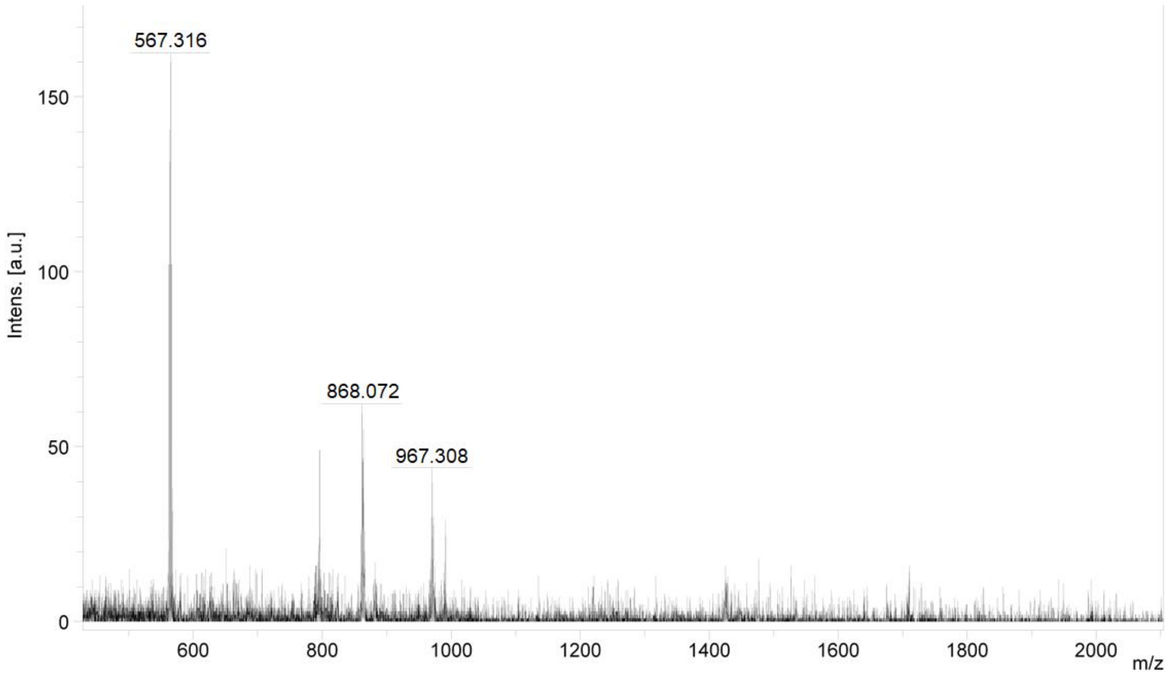

Figure S23. Mass spectra (MALDI-TOF) of *La-L2*

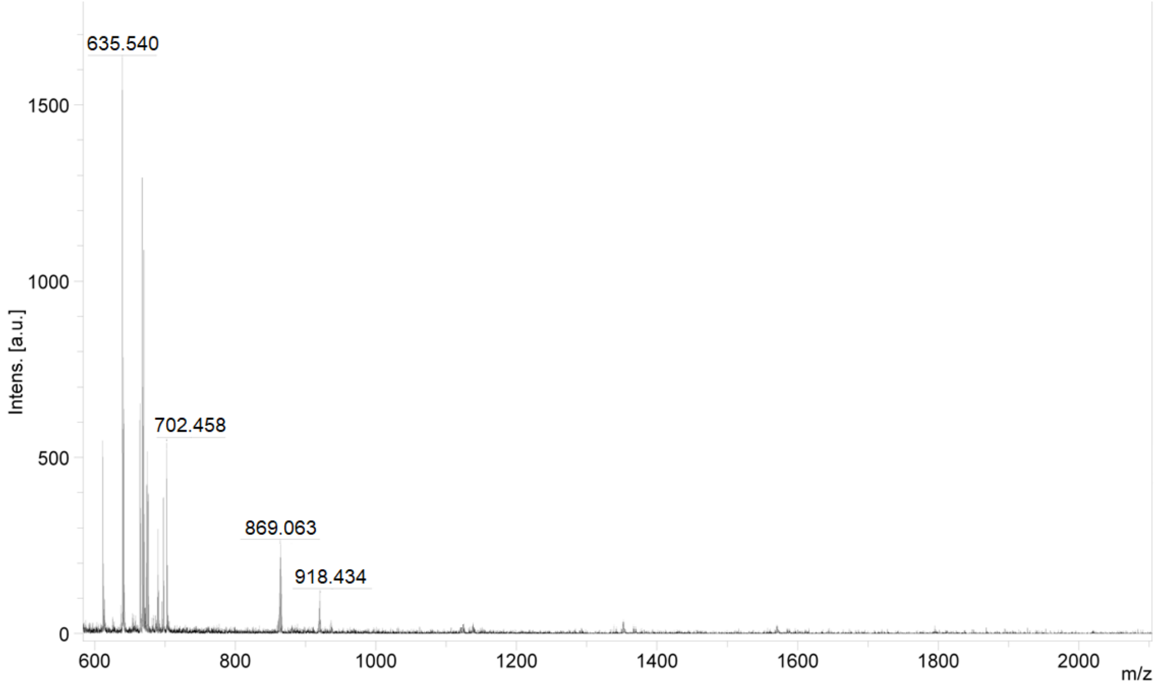

Figure S24. Mass spectra (MALDI-TOF) of *Ce-L2*

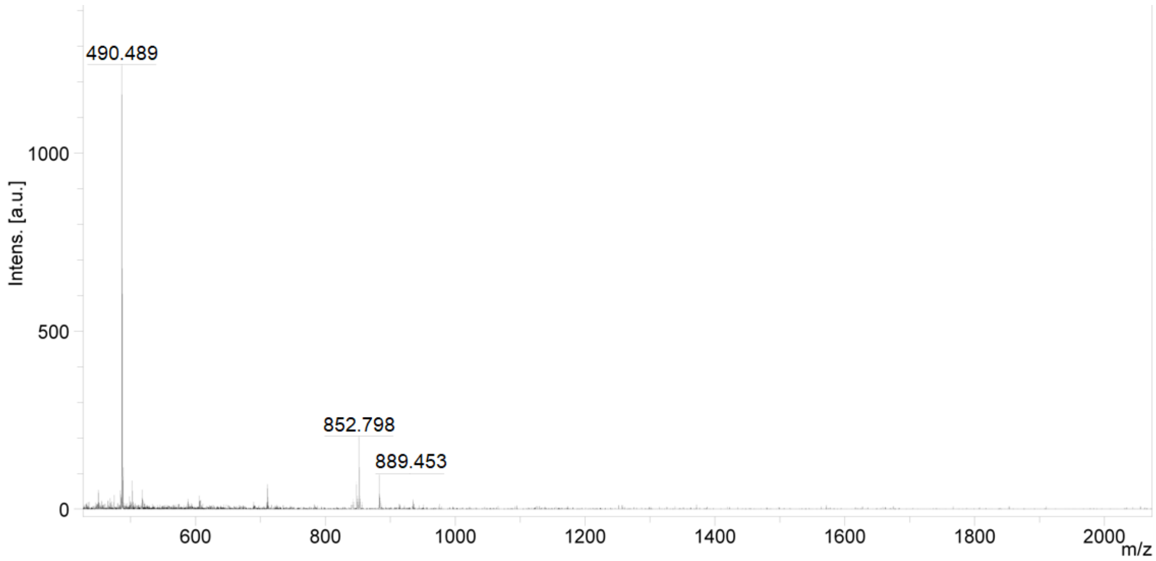

Figure S25. Mass spectra (MALDI-TOF) of *La-L3*

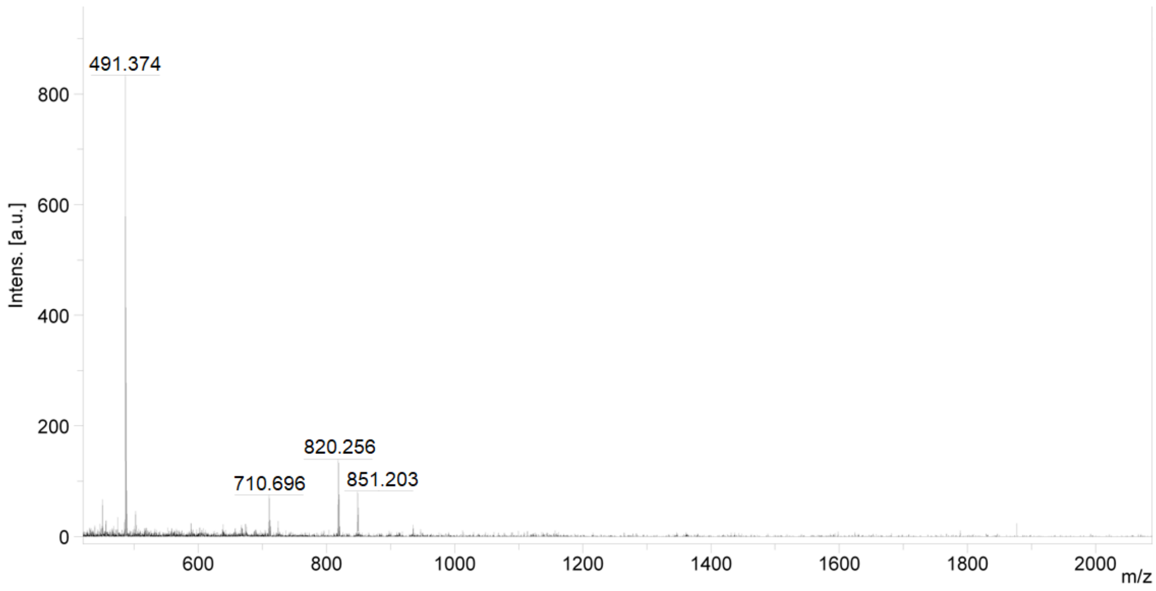

Figure S26. Mass spectra (MALDI-TOF) of *Ce-L3*

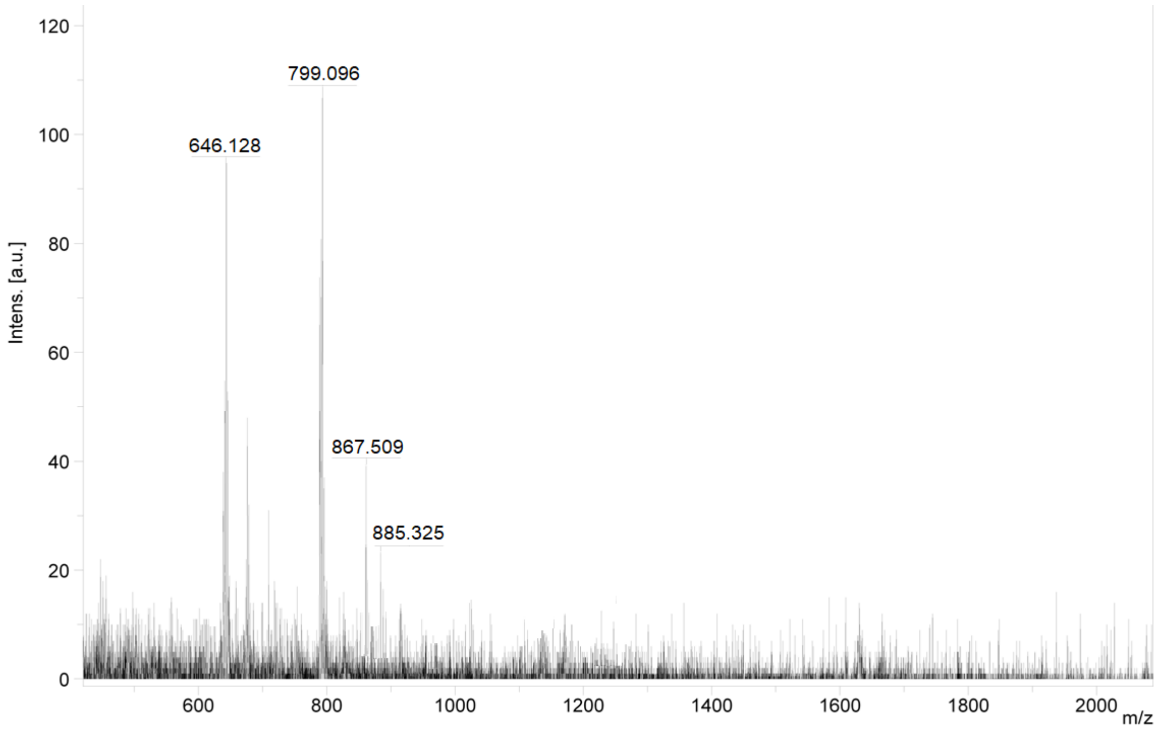

Figure S27. Mass spectra (MALDI-TOF) of *La-L4*

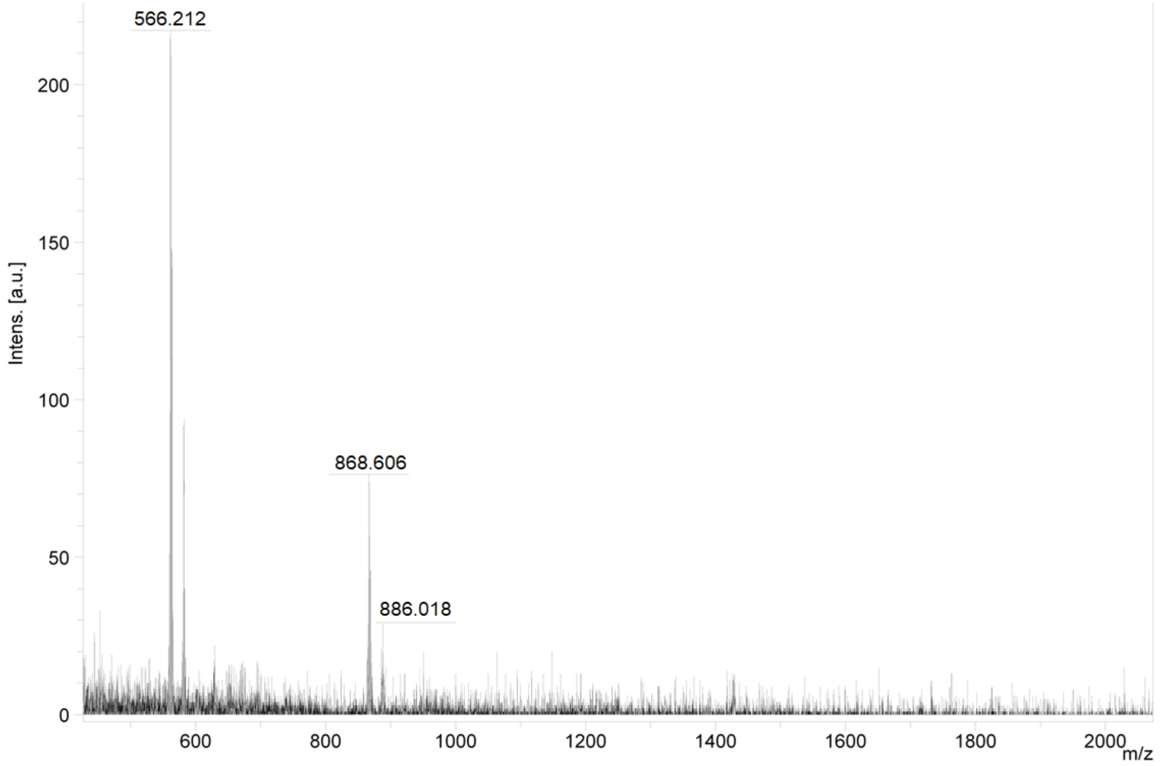

Figure S28. Mass spectra (MALDI-TOF) of *Ce-L4*

4. TG-DTG curves

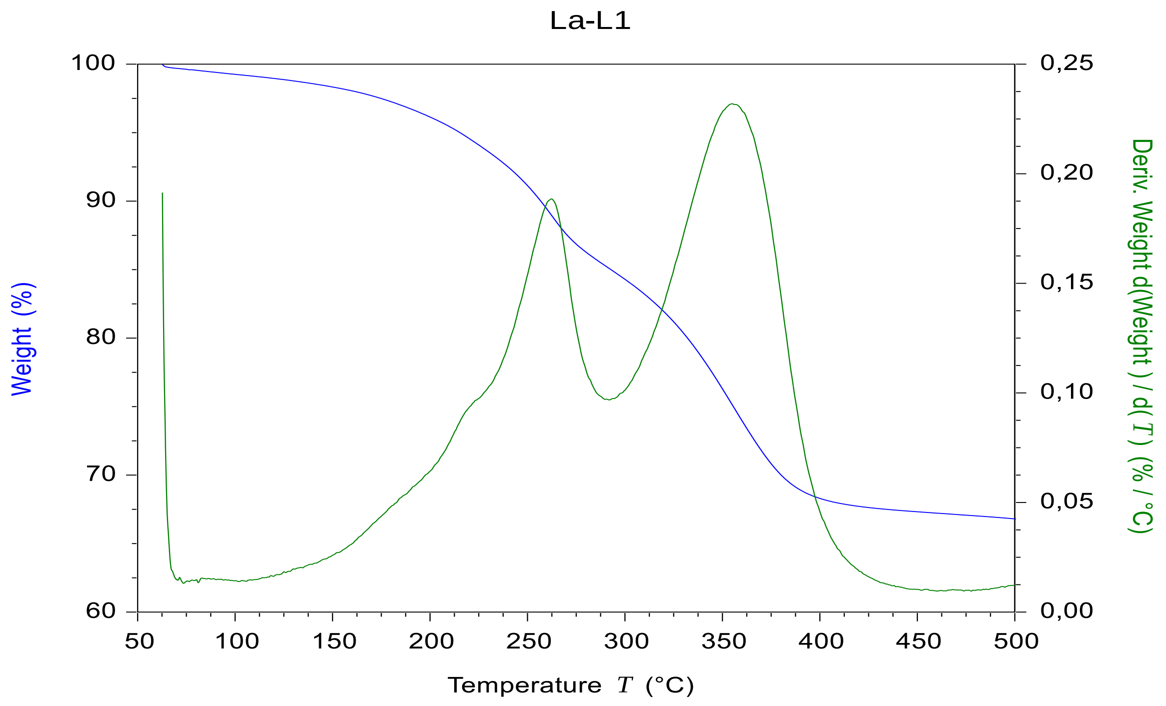

Figure S29. Simultaneous TG-DTG curves of *La-L1*

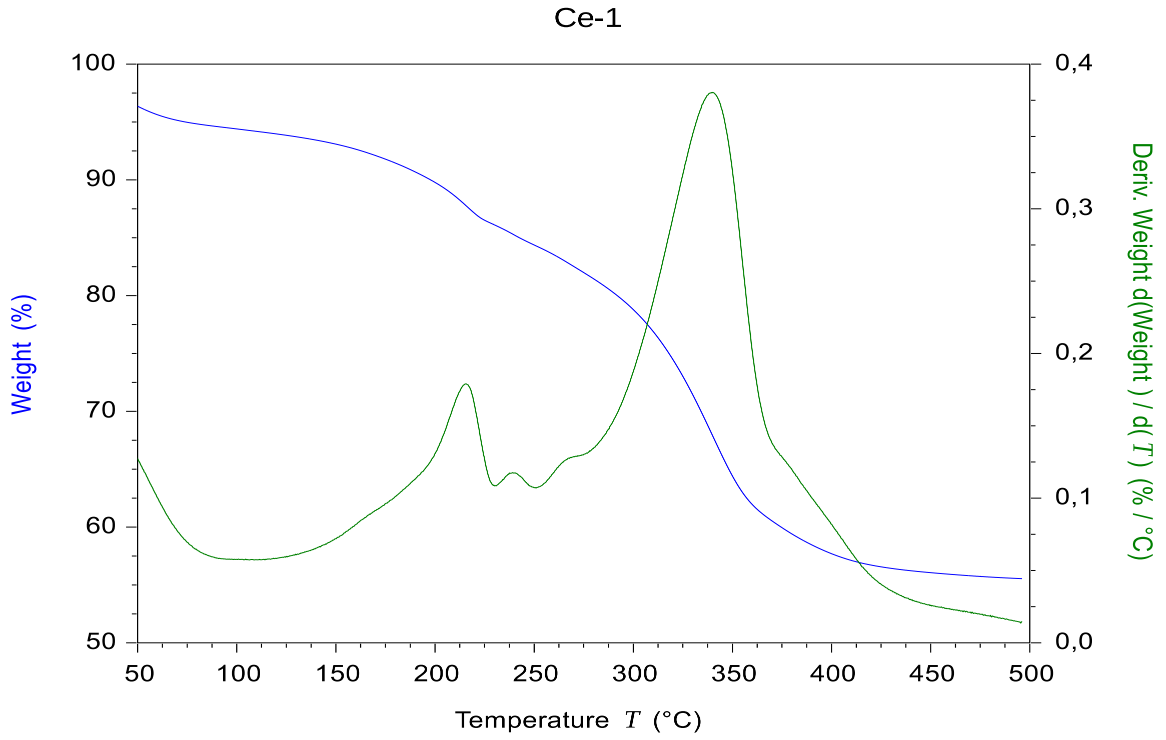

Figure S30. Simultaneous TG-DTG curves of *Ce-L1*

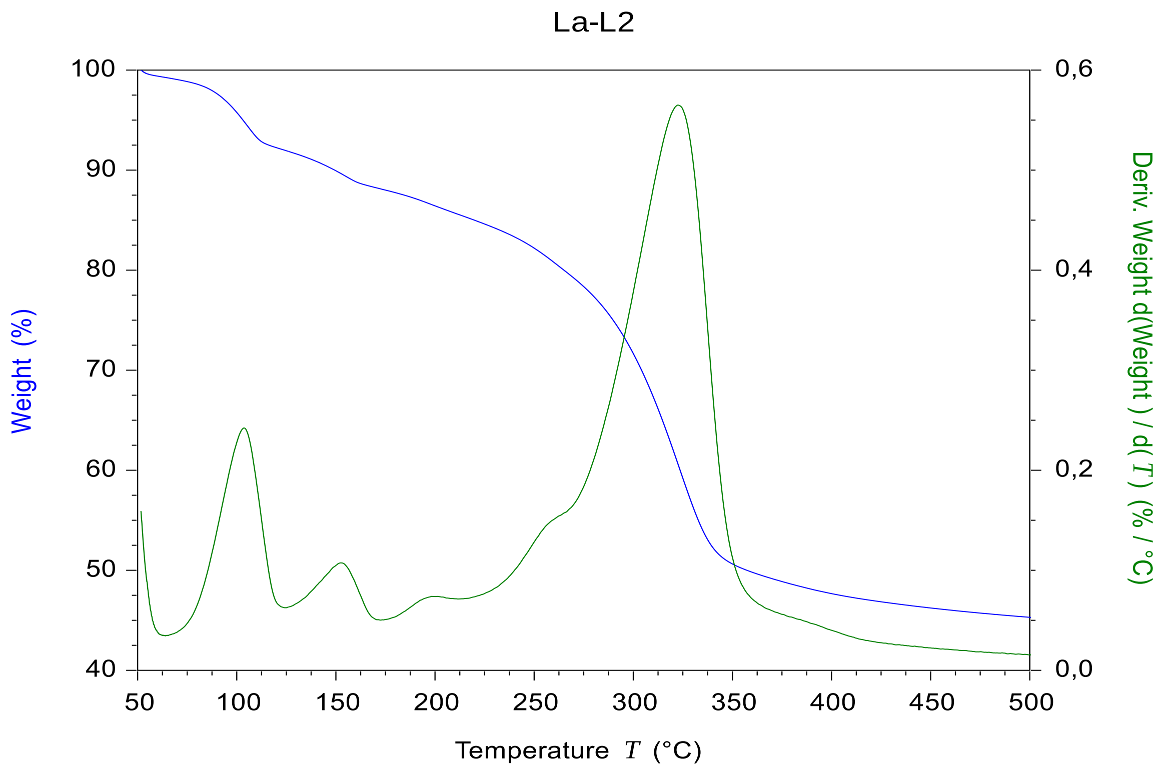

Figure S31. Simultaneous TG-DTG curves of *La-L2*

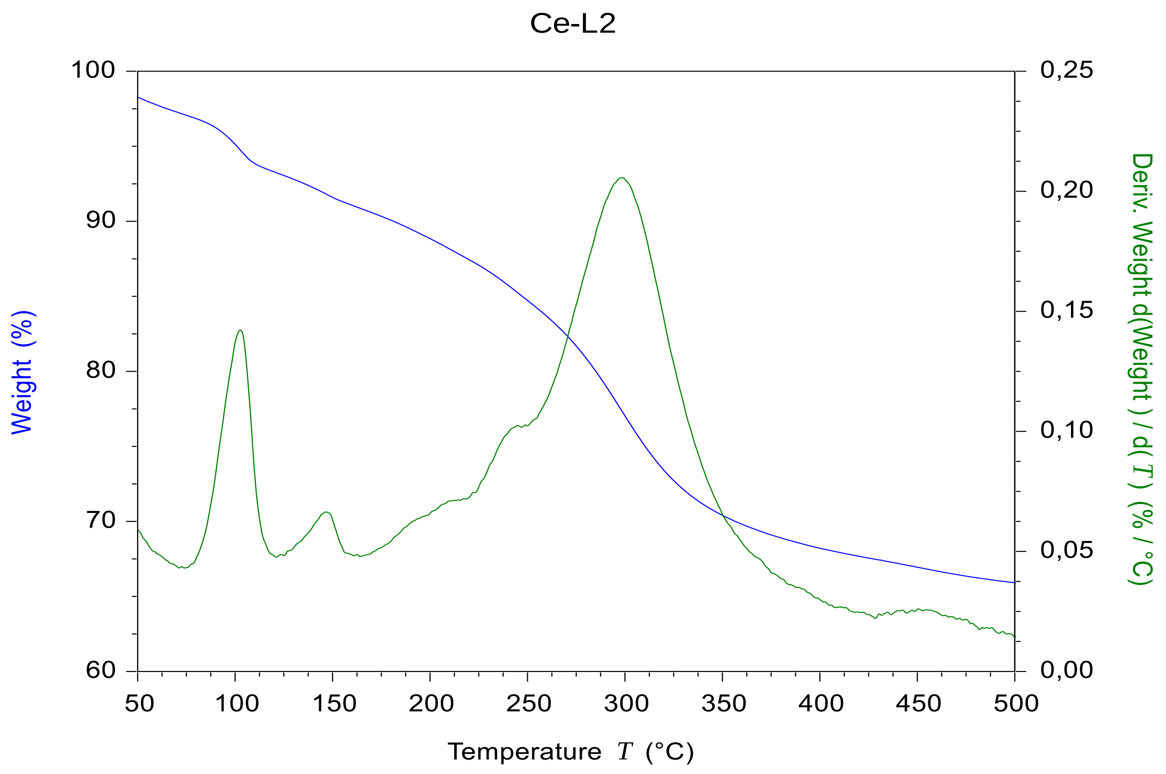

Figure S32. Simultaneous TG-DTG curves of *Ce-L2*

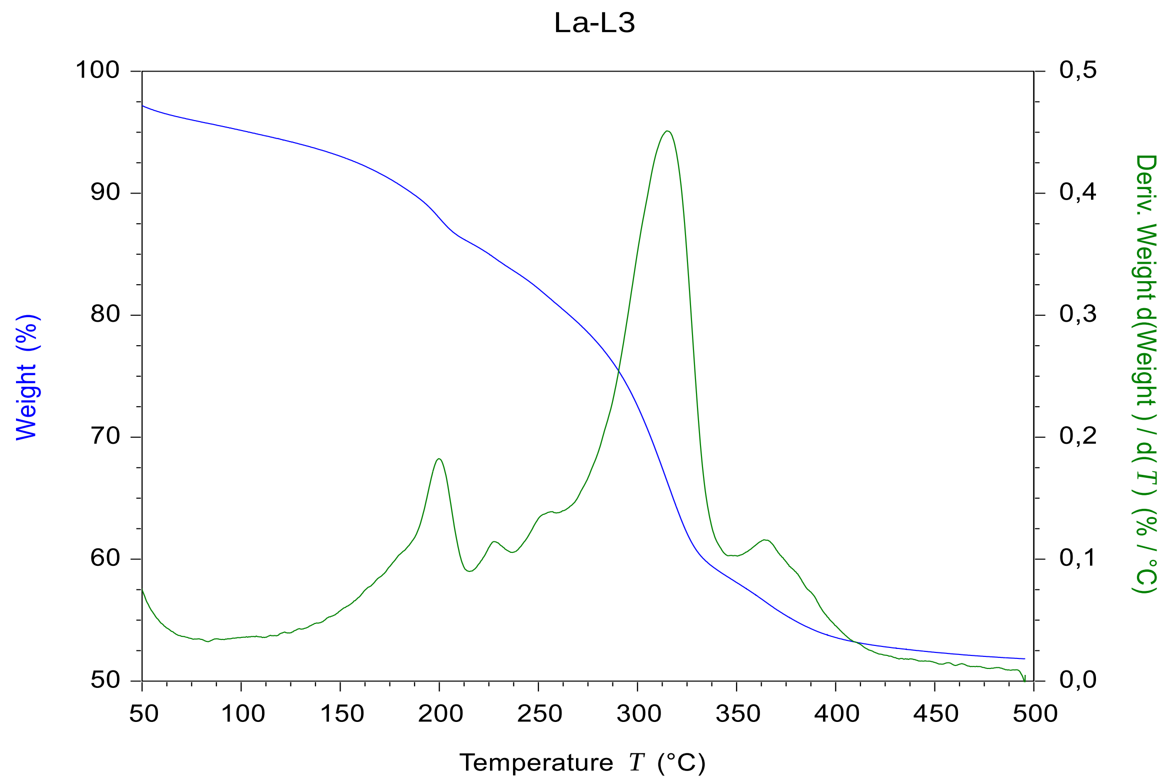

Figure S33. Simultaneous TG-DTG curves of *La-L3*

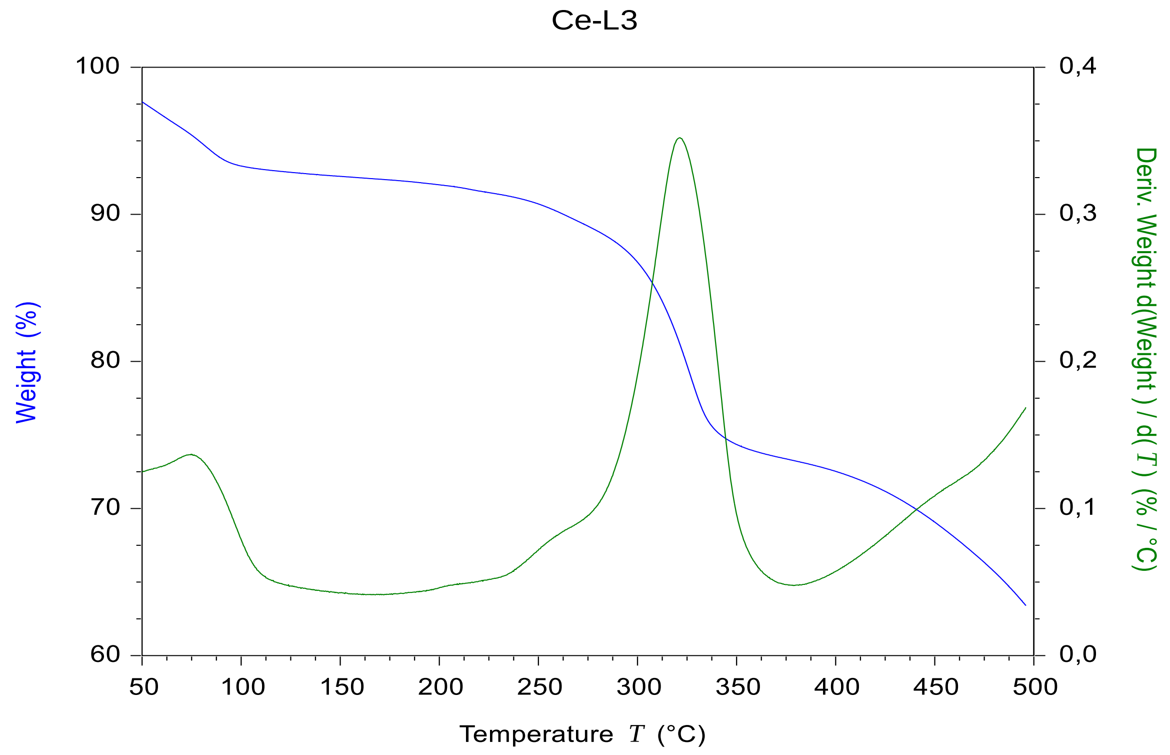

Figure S34. Simultaneous TG-DTG curves of *Ce-L3*

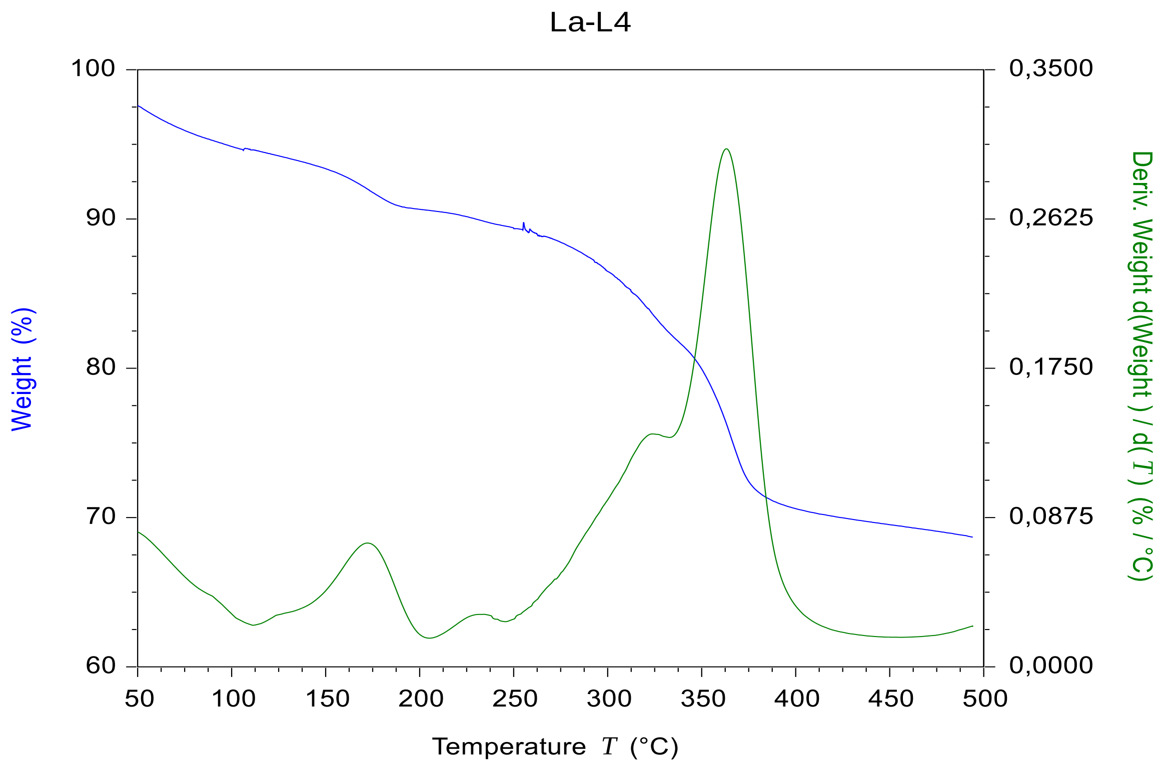

Figure S35. Simultaneous TG-DTG curves of *La-L4*

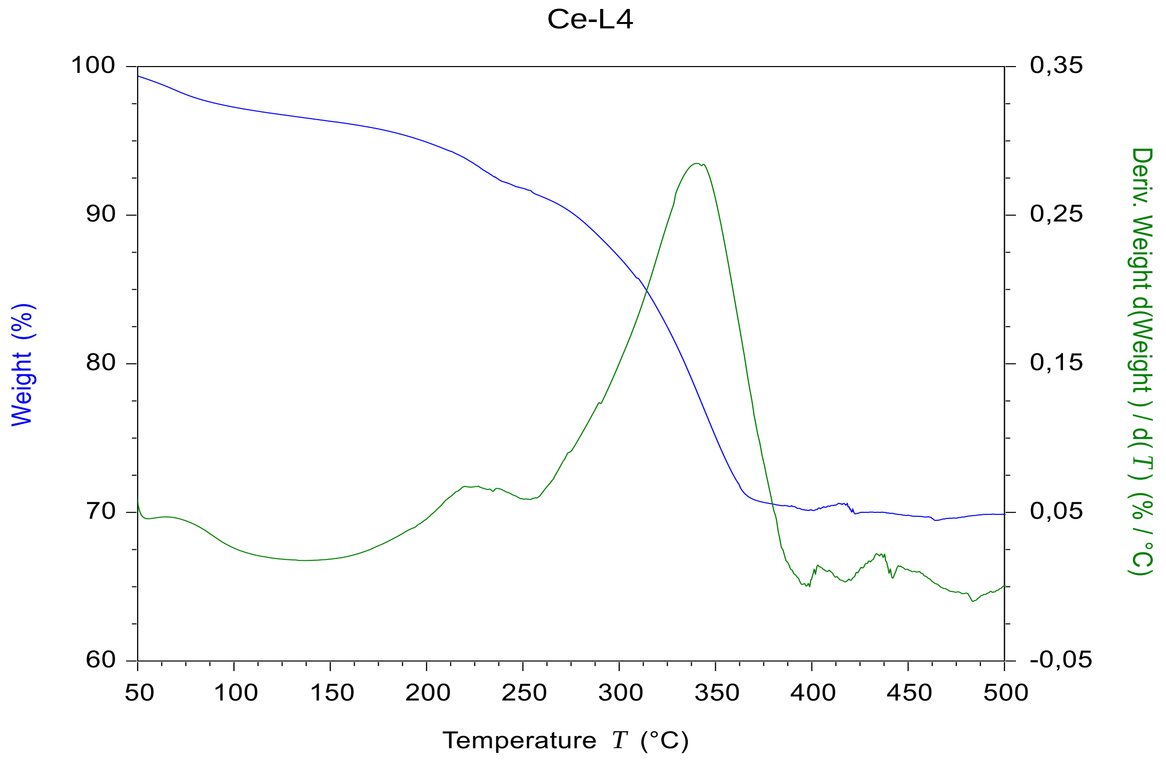

Figure S36. Simultaneous TG-DTG curves of *Ce-L4*

5. UV-Vis spectral data for DNA studies

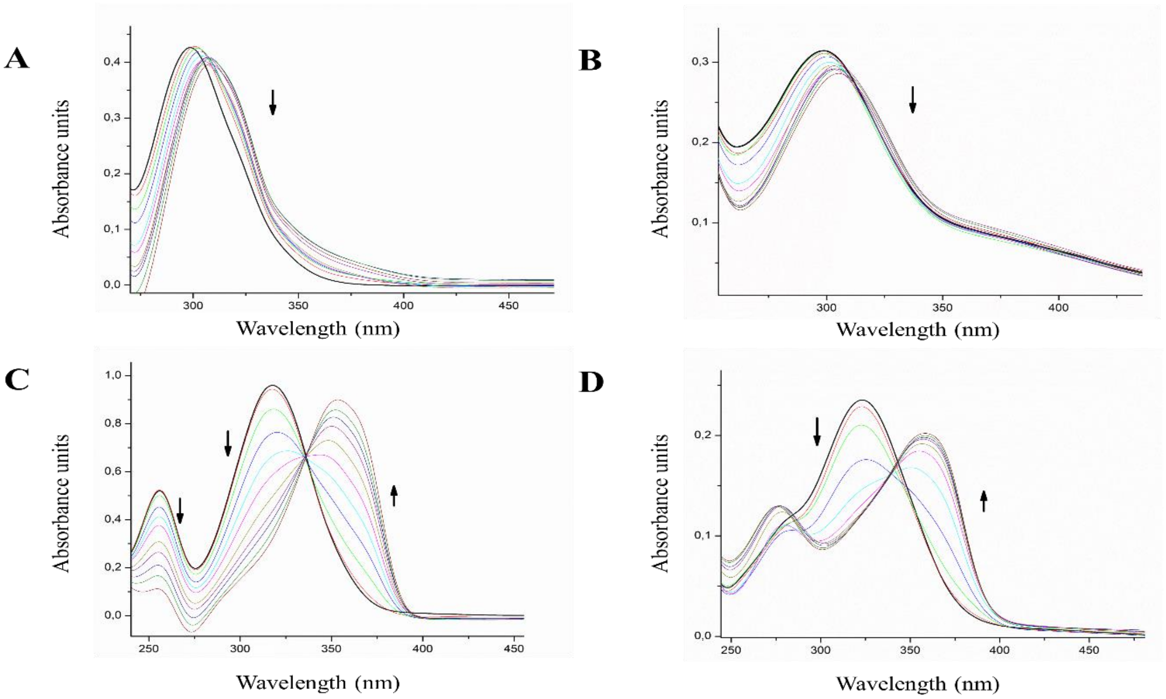

Figure S37. Photometric study at 60 µM of (A) La-L1, (B) La-L2, (C) La-L3 and (D) La-L4, with CT-DNA at concentrations between 0 and 96 µM (solid black line = 0 µM).

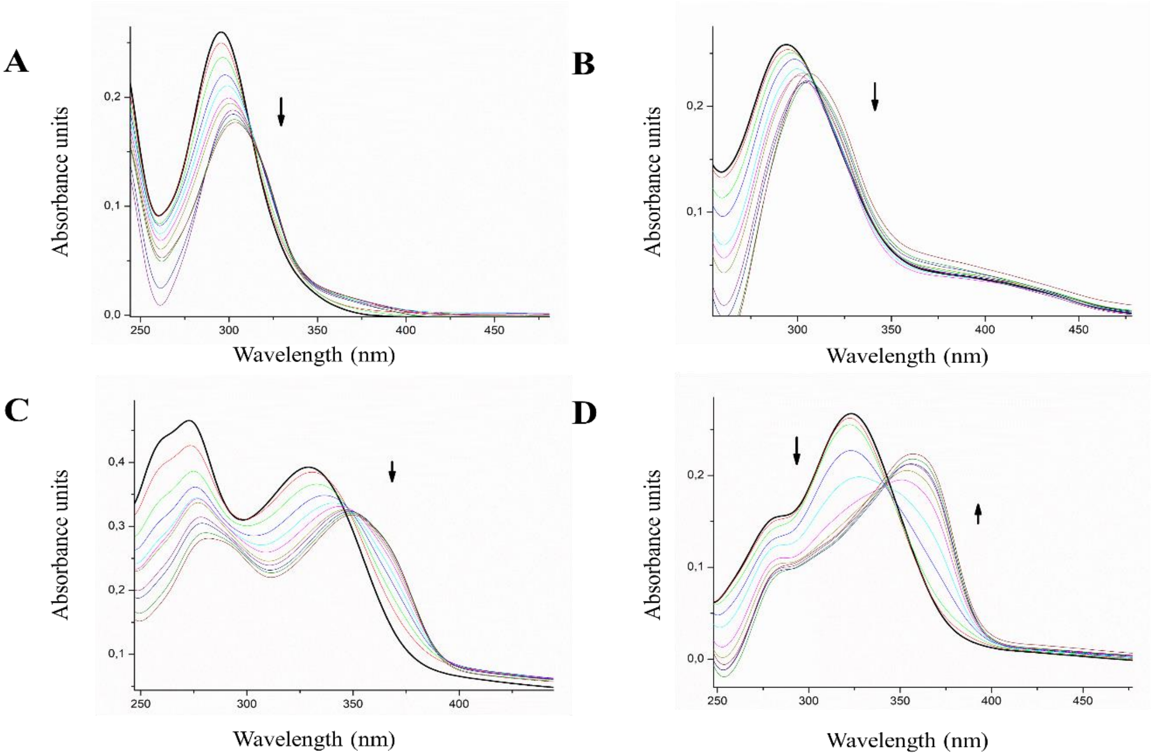

Figure S38. Photometric study at 60 µM of (A) Ce-L1, (B) Ce-L2, (C) Ce-L3 and (D) Ce-L4, with CT-DNA at concentrations between 0 and 96 µM (solid black line = 0 µM).

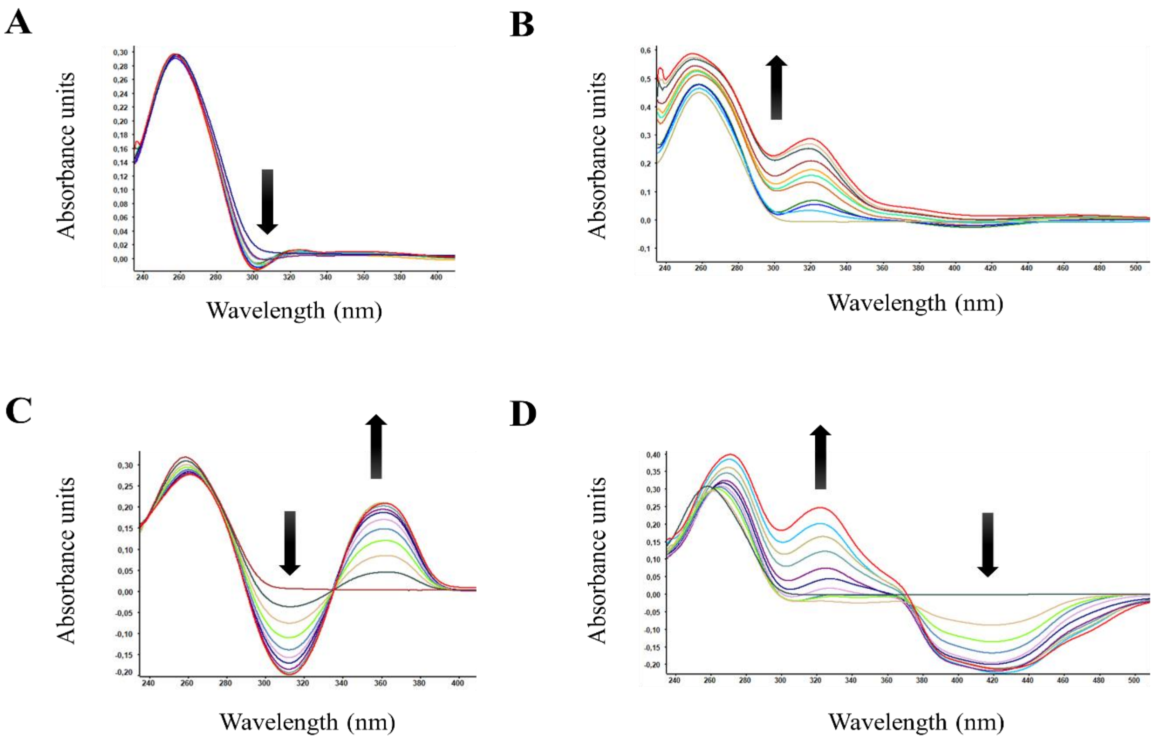

Figure S39. Photometric study of CT-DNA at 48 µM with (A) L1, (B) L2, (C) L3 and (D) L4, at concentrations between 0 and 60 µM.

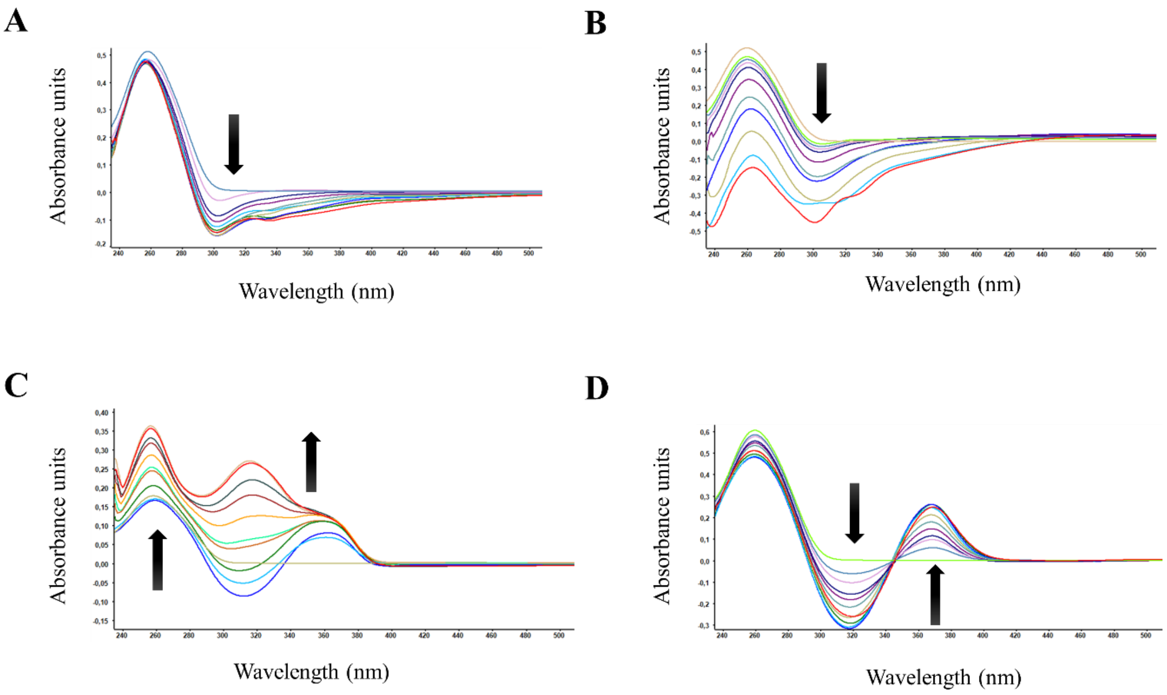

Figure S40. Photometric study of CT-DNA at 48 µM with (A) La-L1, (B) La-L2, (C) La-L3 and (D) La-L4, at concentrations between 0 and 60 µM.

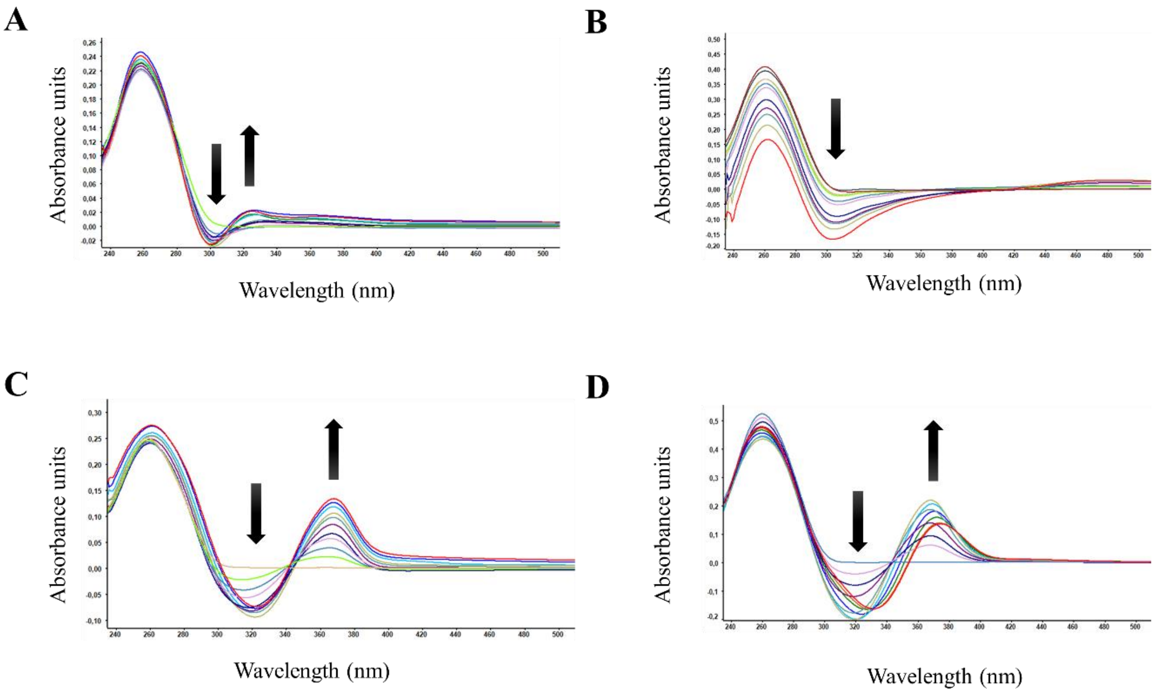

Figure S41. Photometric study of CT-DNA at 48 µM with (A) Ce-L1, (B) Ce-L2, (C) Ce-L3 and (D) Ce-L4, at concentrations between 0 and 60 µM (solid black line = 0 µM).

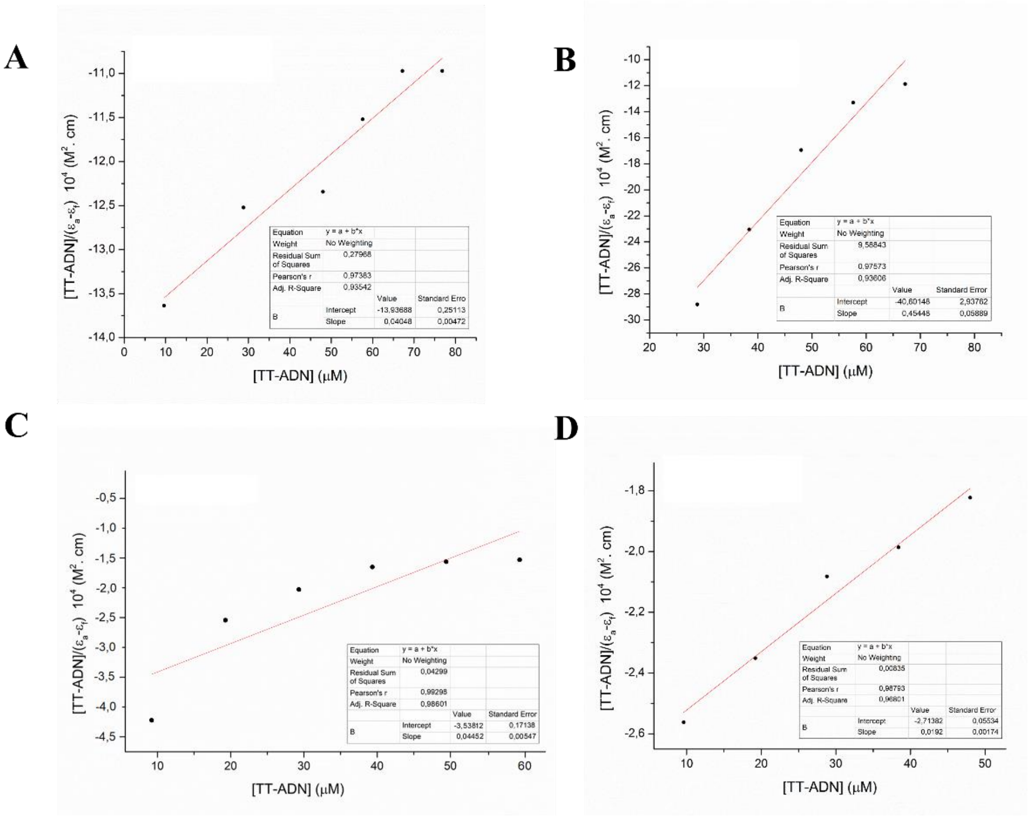

Figure S42. Wolf–Shimer plots for (A) L1, (B) L2, (C) L3 and (D) L4.

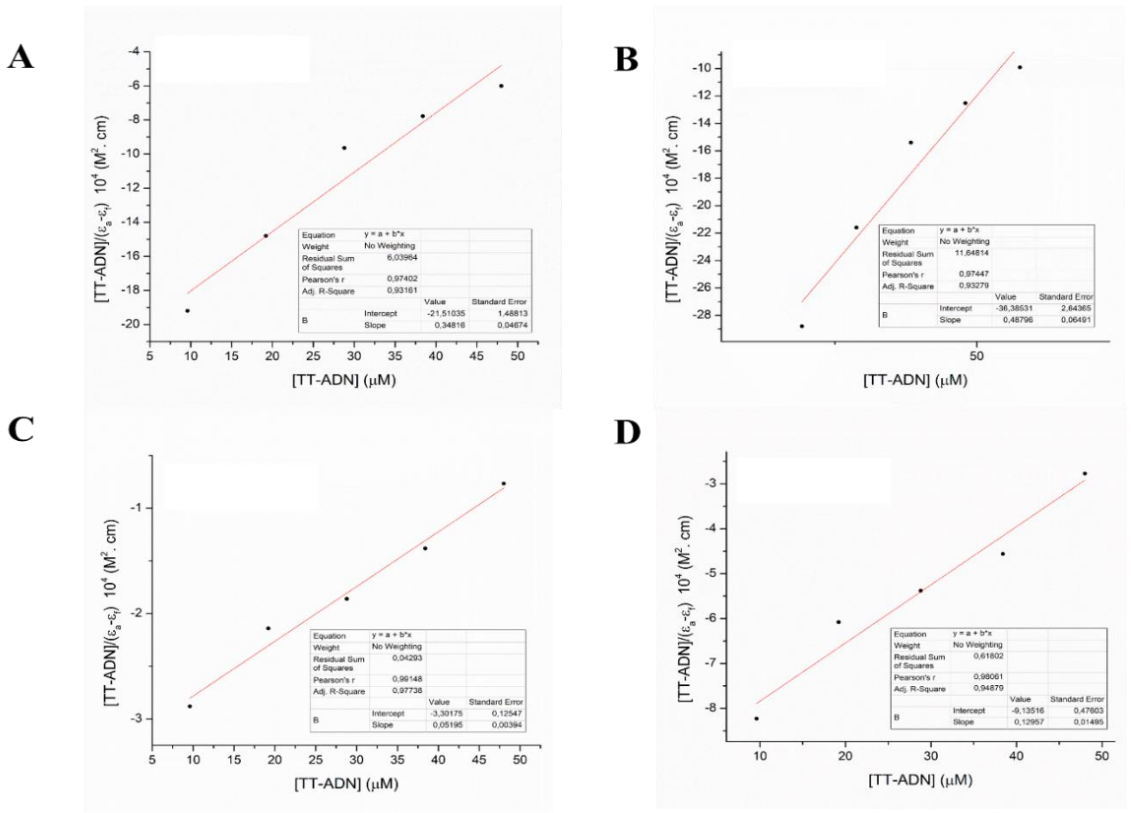

Figure S43. Wolf–Shimer plots for (A) La-L1, (B) La-L2, (C) La-L3 and (D) La-L4.

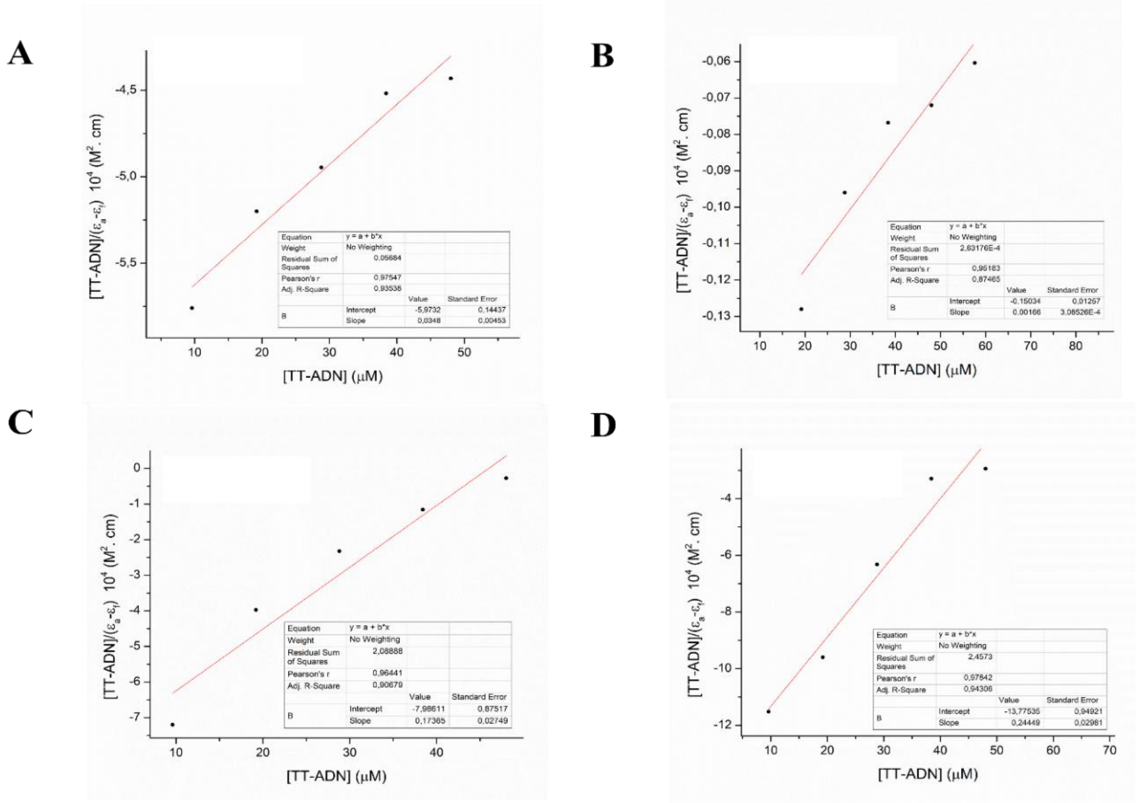

Figure S44. Wolf–Shimer plots for (A) Ce-L1, (B) Ce-L2, (C) Ce-L3 and (D) Ce-L4.

6. Molecular docking studies

Table S3. Interaction of the molecules with the cruzain

Molecule	Binding energy kcal/mol	Hydrogen bonds	Electrostatic bond	Hydrophobic bond
L1	-7.56	Ligand - B:CYS25; Ligand - B:SER64	NA	Ligand - B:ASP161; Ligand - B:ALA138; Ligand - B:CYS25
L2	-6.92	Ligand - A:LEU160; Ligand - B:ASP60; Ligand - B:SER64	NA	Ligand - A:ASP161; Ligand -A:HIS162; Ligand - B:GLY65; Ligand - B:GLY66; Ligand - A:ALA138
L3	-7.86	Ligand - B:CYS63; Ligand - B:CYS25; Ligand- B:GLY66	Ligand - A:ASP161	Ligand - B:HIS162; Ligand - B:GLY163; Ligand - B:ALA138; Ligand - B:CYS25
L4	-7.96	Ligand - B:CYS25; Ligand - B:CYS63	Ligand - A:ASP161	Ligand - B:SER64; Ligand - B:ASP161; Ligand - B:ALA138; Ligand - B:CYS25
La-L1	-11.58	Ligand - B:SER64; Ligand - A:LEU160	NA	NA
La-L2	-10.81	Ligand - B:ASP161; Ligand - B:LEU160; Ligand - B:CYS63; Ligand - B:GLY23; Ligand - A:SER64	NA	Ligand - B:ASP161; Ligand - HIS162; Ligand - B:ALA138
La-L3	-11.1	Ligand - A:CYS25	Ligand - B:ASP161	Ligand - A:ASP161; Ligand - HIS162; Ligand - A:LEU67
La-L4	-10.63	Ligand - A:GLN159; Ligand - B:ASP161; Ligand - B:SER64; Ligand - B:THR59; Ligand - A:LEU160; Ligand - B:CYS25	Ligand - A:GLU239	Ligand - A:PRO213; Ligand - B:ILE309; Ligand - A:VAL277
Ce-L1	-10.88	Ligand- A:SER64; Ligand - A:ASP161; Ligand - B:SER64; Ligand - B:CYS63	NA	Ligand -A:HIS162; Ligand - B:CYS25; Ligand - A:LEU67; Ligand - A:ALA138; Ligand - A:LEU160
Ce-L2	-10.35	Ligand - A:SER142	NA	Ligand - A:TRP184; Ligand - A:CYS22; Ligand - A:CYS25
Ce-L3	-10.98	Ligand - B:SER64; Ligand - A:SER64	NA	Ligand - A:LEU67; Ligand - A:CYS25; Ligand - A:CYS63
Ce-L4	-11.08	Ligand - B:CYS63; Ligand - B:GLU208; Ligand - B:ASP16	Ligand - A:ASP161	Ligand - A:HIS162; Ligand - B:ASP161; Ligand - B:ALA138; Ligand - A:ALA141
Vinilsulfone	-4.3	Ligand - A:VAL139; Ligand - A:ASP161; Ligand - A:HIS162	NA	NA

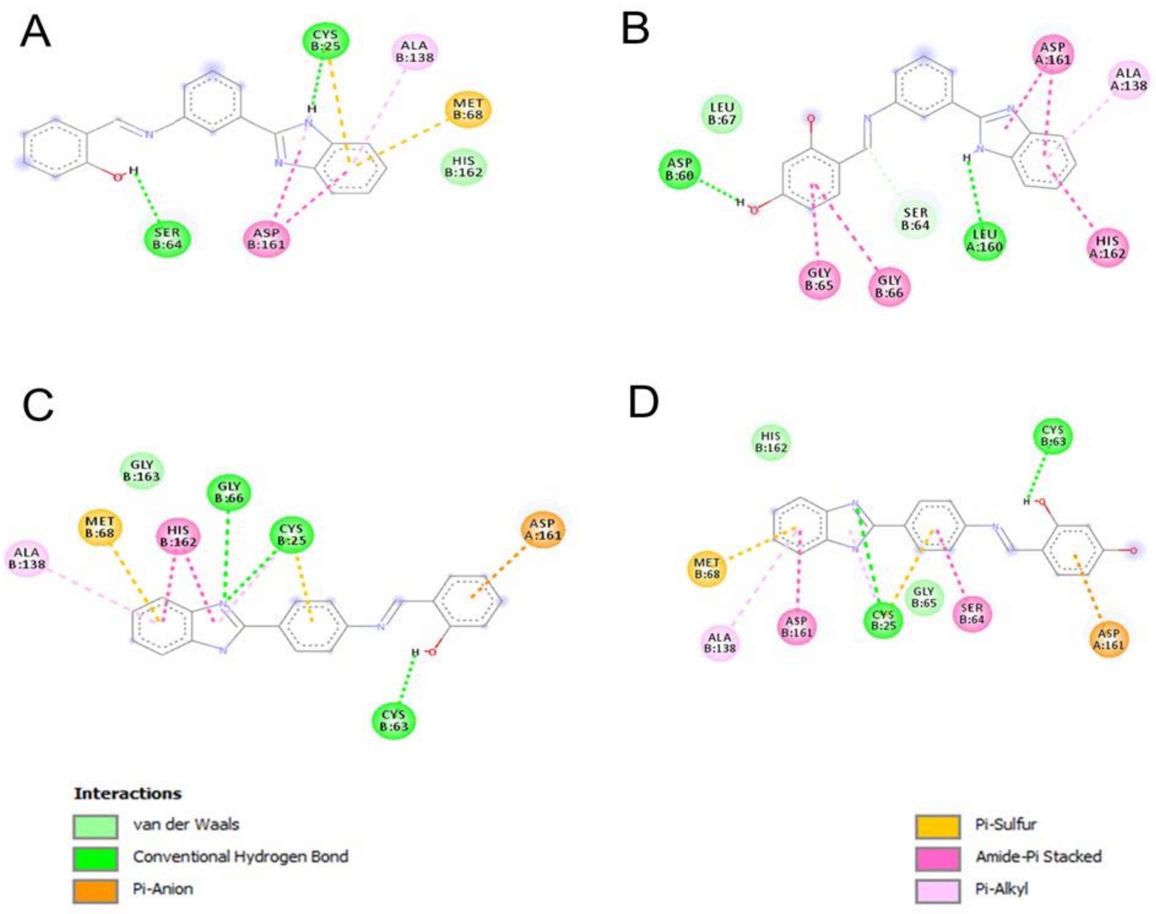

Figure S45. Interaction between ligands and cruzain residues. (A) Ligand 1 (B) Ligand 2 (C) Ligand 3 (D) Ligand 4.

Table S4. Interaction of the molecules with the leishmanin

Molecule	Binding energy kcal/mol	Hydrogen bonds	Electrostatic bond	Hydrophobic bond
L2	-8.78	Ligand - A:ALA349; Ligand - A:GLY222;	Ligand - A:ASP342	Ligand - A:ALA349; Ligand- A:ALA350; Ligand - A:ALA348; Ligand - A:PRO416; Ligand - A:PRO460; Ligand - A:LEU224
La-L2	-9.79	Ligand - A:ALA349; Ligand - A:GLU265	NA	Ligand - A:ALA348; Ligand - A:PRO460; Ligand - A:LEU257; Ligand - A:ALA350; Ligand - A:VAL223; Ligand - A:LEU420
Anfotericina B	-6.80	Ligand - A:LYS573; Ligand - A:GLN166; Ligand - A:THR169; Ligand - A:ASP371; Ligand - A:THR517; Ligand - A:ALA572; Ligand - A:LYS173	NA	NA

Table S5. Interaction of the molecules with alpha tubulin

Molecule	Binding energy kcal/mol	Hydrogen bonds	Electrostatic bond	Hydrophobic bond
L1	-6.78	Ligand - A:PRO261; Ligand - A:ASP431	NA	Ligand - A:TYR262; Ligand - A:TYR453; Ligand - A:PRO263; Ligand - A:ALA451; Ligand - A:VAL435
L2	-6.23	Ligand - A:THR198; Ligand - A:LYS166	Ligand - A:ASP431	Ligand - A:TYR262; Ligand - A:PRO263; Ligand - A:ARG264
L3	-5.48	Ligand - A:ALA451	NA	Ligand - A:ALA451; Ligand - A:TYR453; Ligand - A:PRO263
L4	-5.41	Ligand - A:ASP431	Ligand - A:GLU196	Ligand - A:VAL435; Ligand - A:ARG264
La-L1	-8.46	Ligand - A:PRO261; Ligand - A:PRO263; Ligand - A:ALA451	NA	Ligand - A:PRO263; Ligand - A:ALA451
La-L2	-7.37	Ligand - A:LEU195; Ligand - A:THR198; Ligand - A:LYS166; Ligand - A:LYS163; Ligand - A:HIS266	Ligand - A:LYS166; Ligand -A:ASP199	Ligand - A:TYR453; Ligand - A:ALA451; Ligand - A:PRO263; Ligand - A:LYS163
La-L3	-8.60	Ligand - A:ASP431; Ligand - A:GLU196	Ligand - A:ASP431	Ligand -A:GLU196; Ligand - A:PRO263; Ligand - A:ARG264
La-L4	-7.66	Ligand - A:GLU196; Ligand- A:PRO263; Ligand - A:GLY162; Ligand - A:SER158	NA	NA
Ce-L1	-7.50	Ligand:C - A:ASP199:OD1	Ligand - A:LYS166	Ligand - A:TYR453: Ligand - A:GLU196; Ligand - A:ALA451; Ligand - A:PRO263; Ligand - A:LYS163
Ce-L2	-6.98	Ligand - A:PRO263; Ligand - A:ASP431; Ligand - A:ARG264; Ligand -A:ALA451	Ligand -A:GLU196; Ligand - A:ARG264	Ligand - A:TYR262; Ligand - A:PRO263; Ligand - A:ALA451
Ce-L3	-6.91	Ligand - A:ASP431; Ligand - A:PRO263	Ligand - A:ARG264; Ligand - A:ASP431	Ligand - A:TYR262; Ligand - A:PRO263; Ligand - A:VAL435
Ce-L4	-7.62	Ligand - A:PRO261; Ligand - A:PRO263	Ligand - A:GLU196	Ligand - A:TYR453; Ligand - A:TYR262; Ligand - A:PRO263; Ligand - A:ALA451
Vinblastina	-8.89	Ligand - A:PRO263; Ligand - A:ASP431	NA	Ligand - A:GLU196; Ligand - A:VAL435; Ligand - A:TYR262; Ligand - A:PRO263; Ligand - A:ARG264

Table S6. Interaction of the molecules with *S. aureus* PBP2A

Molecule	Binding energy kcal/mol	Hydrogen bonds	Electrostatic bond	Hydrophobic bond
L1	-7.59	Ligand - A:ASP323; Ligand - B:GLU161; Ligand - B:LYS153	Ligand - B:LYS153; Ligand - B:ASP323	Ligand - A:LYS322; Ligand - A:ASP323; Ligand - B:ASP323; Ligand- A:LYS322; Ligand - B:LEU155; Ligand - A:LEU155
L2	-7.70	Ligand - B:LYS322; Ligand - A:GLU161; Ligand - B:ASP320; Ligand - B:ASP323; Ligand - A:LYS153	Ligand - A:ASP323; Ligand - B:ASP323	Ligand - B:LEU155; Ligand - A:LEU155; Ligand - B:LYS322
L3	-7.03	Ligand - A:GLN325; Ligand - B:ASP323	Ligand - A:GLU161	Ligand - B:ASP323; Ligand - B:LYS322; Ligand - A:LEU155; Ligand - B:LEU155
L4	-6.65	Ligand - B:ASP323; Ligand - B:GLU161; Ligand - A:GLU161	Ligand - A:ASP323	Ligand - B:LEU155; Ligand - A:LEU155
La-L1	-10.01	Ligand - A:LYS148	Ligand - A:LYS148; Ligand -A:GLU170; Ligand - A:ASP275	Ligand - A:PRO258; Ligand - A:VAL277; Ligand - A:ARG151; Ligand - A:ARG241; Ligand - A:VAL256
La-L2	-8.47	Ligand - A:LYS148; Ligand - A:ARG151; Ligand - A:ASP275; Ligand - A:ASP295; Ligand - A:HIS293; Ligand - A:VAL277	Ligand - A:LYS148; Ligand - A:HIS293	Ligand - A:HIS293; Ligand - A:PRO258; Ligand - A:VAL277
La-L3	-9.52	Ligand - A:GLU239; Ligand - A:ASP275; Ligand - A:GLU170	Ligand - A:LYS148; Ligand -A:ASP275	Ligand - A:THR238; Ligand - A:PRO213; Ligand - A:VAL277
La-L4	-8.36	Ligand- A:GLU239; Ligand - A:PHE211; Ligand - A:THR238G1	NA	Ligand -A:PRO213; Ligand -B:ILE309; Ligand - A:VAL277
Ce-L1	-9.87	Ligand - A:LYS148; Ligand- A:GLU17	Ligand - A:LYS148; Ligand - A:GLU239	Ligand - A:VAL256; Ligand - A:VAL277; Ligand - A:MET372
Ce-L2	-9.60	Ligand - A:GLU239; Ligand - A:THR238; Ligand - A:ASP295; Ligand - A:HIS293	Ligand - A:LYS148; Ligand - A:GLU170; Ligand - A:ASP275	Ligand - A:SER240; Ligand - A:ARG151; Ligand - A:ARG241; Ligand - A:VAL277
Ce-L3	-8.96	Ligand - A:HIS293	Ligand - A:LYS148; Ligand - A:HIS293; Ligand - A:GLU170; Ligand - A:GLU239; Ligand - B:GLU315	Ligand - A:GLU239; Ligand - A:ARG151; Ligand - A:ARG241; Ligand -A:VAL277; Ligand - A:HIS293; Ligand - A:PRO213
Ce-L4	-8.48	Ligand - A:THR238; Ligand - A:VAL277; Ligand -A:ARG241; Ligand -A:HIS293; Ligand - A:ASN164; Ligand - A:GLN292; Ligand - A:GLU239; Ligand - A:ASP275	Ligand - A:ASP275	Ligand - A:ARG151; Ligand - A:ARG241; Ligand - A:ALA276; Ligand - A:VAL277

Cefepime	-5.93	Ligand - A:LYS148; Ligand - A:ARG151; Ligand - A:THR165; Ligand - A:HIS293; Ligand - A:GLU239; Ligand - A:ARG241	Ligand - A:ARG241	NA
Ceftobiprole	-7.06	Ligand - A:GLU239; Ligand - A:GLU294; Ligand - A:ASP295; Ligand - A:LYS148; Ligand - A:GLN292; Ligand - A:HIS293	NA	Ligand - A:GLU239; Ligand - A:LYS273; Ligand - A:ALA276; Ligand - A:VAL277; Ligand - A:ARG151; Ligand - A:ARG241; Ligand - A:HIS293
Benzopenicillin	-6.23	Ligand - A:ARG241; Ligand - A:HIS293	Ligand - A:HIS293	Ligand - A:HIS293; Ligand - A:LYS273; Ligand - A:ALA276
